# IER3IP1-mutations cause microcephaly by selective inhibition of ER-Golgi transport

**DOI:** 10.1007/s00018-024-05386-x

**Published:** 2024-08-08

**Authors:** Mihaela Anitei, Francesca Bruno, Christina Valkova, Therese Dau, Emilio Cirri, Iván Mestres, Federico Calegari, Christoph Kaether

**Affiliations:** 1 Leibniz Institute on Aging, Fritz-Lipmann-Institute, Beutenbergstr 11, 07745 Jena, Germany; 2 Center for Regenerative Therapies, TU-Dresden, Fetscherstraße 105, 01307 Dresden, Germany

**Keywords:** Endoplasmic reticulum, COPII, Anterograde transport, Microcephaly, Diabetes, Axon pathfinding, Cortical development

## Abstract

Mutations in the *IER3IP1* (Immediate Early Response-3 Interacting Protein 1) gene can give rise to MEDS1 (Microcephaly with Simplified Gyral Pattern, Epilepsy, and Permanent Neonatal Diabetes Syndrome-1), a severe condition leading to early childhood mortality. The small endoplasmic reticulum (ER)-membrane protein IER3IP1 plays a non-essential role in ER-Golgi transport. Here, we employed secretome and cell-surface proteomics to demonstrate that the absence of IER3IP1 results in the mistrafficking of proteins crucial for neuronal development and survival, including FGFR3, UNC5B and SEMA4D. This phenomenon correlates with the distension of ER membranes and increased lysosomal activity. Notably, the trafficking of cargo receptor ERGIC53 and KDEL-receptor 2 are compromised, with the latter leading to the anomalous secretion of ER-localized chaperones. Our investigation extended to in-utero knock-down of *Ier3ip1* in mouse embryo brains, revealing a morphological phenotype in newborn neurons. In summary, our findings provide insights into how the loss or mutation of a 10 kDa small ER-membrane protein can cause a fatal syndrome.

**Supplementary Information:**

The online version contains supplementary material available at 10.1007/s00018-024-05386-x.

## Introduction

Microcephaly is characterized by a reduction of the head circumference by more than 2 standard deviations below the age and sex-corrected average. It can be caused by reduced proliferation or cell death of neuroprogenitor cells [1], as well as by defective neuronal morphology or migration [2, 3]. Microcephaly, epilepsy, and diabetes syndrome-1 (MEDS1, OMIM #614231) is an autosomal recessive neurodevelopmental disorder characterized by microcephaly, simplified gyral pattern, severe epilepsy, and early-onset infantile diabetes mellitus caused by mutations in the* IER3IP1 *gene [4]. Patients suffering from MEDS1 present dysmorphic facial features, skeletal deformities, and die in early childhood at the age of 1.5–8 years [5, 6]. Two point mutations in *IER3IP1* causing early death between 1.5 and 5.5 years have been identified to date, p.V21G and p.L78P [4, 5, 7]. Additionally, one patient with slightly milder symptoms and death at 8 years of age has been described with a compound heterozygosity in *IER3IP1*, with one allele carrying the p.V21G variant, and the other a c.79delT deletion (p.T79∆) resulting in a frameshift mutation (p.Phe27fsSer*25) [6]. Recently, a MEDS1 patient with a third, homozygous, p.A18V variant has been identified [8]. IER3IP1 is an 82-amino acid protein with two predicted transmembrane domains, that localizes to the endoplasmic reticulum (ER) [4, 9]. In mouse, *Ier3ip1* is highly expressed during embryogenesis, especially at sites of neurogenesis (i.e., cortical ventricular and subventricular zones) [4]. Its yeast orthologue, Yos1p, together with Yip1p and Yif1p, forms a tripartite complex essential for ER-Golgi transport [10]. Interestingly, mutations in the human orthologue of *Yip1p*, *YIPF5*, cause MEDS2 (OMIM #614,231), a syndrome similar to MEDS1 [11]. The third complex subunit, *Yif1p*, has two human orthologues, *YIF1A *and* YIF1B*, and*YIF1B* mutations can also cause microcephaly and epilepsy [12], suggesting common pathomechanisms of the *YIPF5*,*YIF1B *and* IER3IP1* disease-causing variants.

At the cellular level, loss of IER3IP1 induces apoptotic cell death in pancreatic β-cells [4] and in mouse insulinoma cells [13], where it also impairs the reaction to ER stress, the unfolded protein response (UPR). A mouse model with β-cell-specific deletion of *Ier3ip1* presents, in contrast, increased ER-stress without an evident UPR activation [14]. The ER is dilated in these cells, and proinsulin folding is impaired. In human brain organoids, IER3IP1 determines size and extracellular matrix composition [15]. These studies indicate an important role for IER3IP1 in the ER, however, its function in ER to Golgi transport and its potential cargos in mammalian cells are not well understood. We here provide a detailed analysis of IER3IP1 function and show that it governs the ER-export of a subset of secretory and membrane-bound cargos. The ER-export of several factors important for neuronal migration, axon pathfinding and neuronal survival is reduced, and the localization of ERGIC53 and KDEL-receptor 2 are changed, the latter causing an abnormal secretion of ER-localized enzymes. We also provide a comparative functional study of known pathogenic variants, which indicate that *IER3IP1* p.L78P, p.T79∆, but not p.V21G, disrupt the ER-export of specific cargos. Finally, we model the loss of *IER3IP1* during mammalian brain development by in utero electroporation showing its importance for neuronal morphology.

## Methods

**Table:** 

Antibodies	Source	Application and dilution (IB, immunoblot; IF, immunofluorescence; IP, immunoprecipitation)
P62/SQSTM1 clone 2C11	H00008878-MO1, Abnova	IB 1:1000
ATF6	ab227830, Abcam	IB 1:1000
V5 Tag monoclonal antibody SV5-Pk1	R960-25, Thermo Fischer	IB 1:1:1000, IF 1:100
GFP mouse IgG_1_κ (clones 7.1 and 13.1)	1181446001, Roche, Sigma-Aldrich, Merck	IB 1:1000, IP 2 µl
FGF Receptor 3 (C51F2)	4574, Cell Signaling Technology	IB: 1:1000
ST6GAL1	AF5924, R&D Systems	IF 1:100
LC3B	NB100-2220, Novus Biologicals, Bio-Techne	IB 1:1000
IER3IP1	HPA010027, Atlas Antibodies, Sigma-Aldrich ab181247, Abcam	IB 1:500, IF 1:40 (Atlas) IB 1:1000 (Abcam)
alpha-tubulin clone DM1A	T6199, Sigma-Aldrich	IB 1:1000
beta-actin	ab8227, Abcam	IB 1:1000
Flag clone M2	F1804, Sigma-Aldrich	IB 1:1000, IF 1:200
BIP	3177, Cell Signaling Technology	IB 1:1000
recombinant anti-IRE1 (phospho S724) EPR5253	ab 124945, Abcam	IB 1:1000
IRE-1a (14C10)	3294, Cell Signaling Technology	IB 1:1000
Cas3 clone C92-605	559565, BD Biosciences	IF 1:600 (brain sections) IF 1:200 (i3N)
GFP-1020 IgY	Aves Labs, 2BScientific, UK	IF 1:400
LAMP1 human CD107a, clone H4A3	555798, BD Biosciences;	IF 1:100
calnexin	ADI-SPA-860D, Enzo Life Science	IF 1:50
Sec31A clone 32	612350, BD Biosciences	IB 1:1000, IF 1:200
Sec16A	ab70722, Abcam	IB 1:1000, IF 1:200
ERGIC53 cl B9	sc-271517, Santa Cruz Biotechnology	IF 1:200
GM130 clone 35	610,822, BD Biosciences	IF 1:200
beta tubulin III	T2200, Sigma-Aldrich	IF 1:200
MAP-2 clone HM-2	M4403, Sigma-Aldrich	IF 1:200
Sox2 monoclonal antibody (Btjce)	14–9811-82 eBioscience^™^, Thermo Fisher	IF 1:200
Tbr2/Eomes	ab23345, Abcam	IF 1:600
Ki67	ab833, Abcam	IF 1:500
Normal Rabbit IgG	2729, Cell Signaling Technology	
HRP-anti-Rabbit IgG(H + L)	W4011, Promega GmbH	IB: 1:5000
HRP-anti-Mouse IgG(H + L)	W4021, Promega GmbH	IB: 1:2000
HRP-Donkey anti-goat IgG	sc-2020, Santa Cruz Biotechnology	IB: 1:2000
Donkey anti-Mouse IgG (H + L) 555	A-31570, Invitrogen^™^, Thermo Fisher	IF: 1:500
Goat anti-Mouse IgG (H + L) 488	A-11001, Invitrogen^™^, Thermo Fisher Scientific	IF: 1:500
Goat anti-Mouse IgG (H + L) 555	A-21422, Invitrogen^™^, Thermo Fisher	IF: 1:500
Goat anti-Mouse IgG (H + L) 594	A-11005, Invitrogen^™^, Thermo Fisher	IF: 1:500
Rabbit IgG (H + L)	A-11034, Invitrogen^™^, Thermo Fisher	IF: 1:500
Goat anti-Rabbit IgG (H + L) 555	A-21428, Invitrogen^™^, Thermo Fisher	IF: 1:500
Goat anti-Rabbit IgG (H + L) 647	A-21244, Invitrogen^™^, Thermo Fisher	IF: 1:500
Goat anti-Rat IgG (H + L) 488	A-11006, Invitrogen^™^, Thermo Fisher	IF: 1:500
Goat anti-Rat IgG (H + L) 555	A-21434, Invitrogen^™^, Thermo Fisher	IF: 1:500
Donkey anti-Sheep IgG (H + L) 555	A-21436, Invitrogen^™^, Thermo Fisher	IF: 1:500
Donkey anti-Goat IgG (H + L)	A-11055, Invitrogen^™^, Thermo Fisher	IF: 1:500
Donkey anti-Chicken IgY (H + L) 488	A78948, Invitrogen^™^, Thermo Fisher	IF: 1:500

**Table:** 

Reagents	Source	
Hoechst 33,342	H3570, Invitrogen, Thermo Fisher	
Magic Red Cathepsin L	ICT-941, Biomol GmbH	
Mowiol 4–88 reagent	475904, Millipore Sigma, Merck	
brefeldin A	B7651, Sigma-Aldrich	
MG132	474790, Calbiochem, Merck	
DMSO	D8418, Sigma-Aldrich	
torin 1	#4247, Bio-Techne/Tocris	
EBSS	24010043, Thermo Fisher	
chloroquine	C6628, Sigma-Aldrich	
biotin	B4639, Sigma-Aldrich	
thapsigargin	T9033, Sigma-Aldrich	
PhosSTOP^™^ Phosphatase Inhibitor Tablets	4906845001, Roche, Merck	
cOmplete^™^ EDTA-free protease inhibitor cocktail	5056489001, Roche, Merck	
protease inhibitor cocktail	P8340, Sigma-Aldrich, Merck	
Hygromicin B	10687010, Thermo Fisher	
CHAPSO	Carl Roth, Germany	
GoTaqG2 polymerase	M7841, Promega	
NEBuilder^®^ HiFi DNA Assembly Master Mix	E2621, New England Biolabs	
EndoH	P0702, New England BioLabs	
PNGaseF	P0704, New England BioLabs	
SYTOX^™^ Blue Dead Cell Stain	S34857, Invitrogen, Thermo Fisher	
Dulbecco’s Modified Eagle Medium (1x) + Glutamax^™^	61965–026, Gibco, ThermoFisher	
Fetal Bovine Serum	F7524 Sigma-Aldrich	
1 × Penicilin-Streptomycin	P0781 Sigma-Aldrich	
Trypsin–EDTA	25300054, Gibco	
Lipofectamine^™^ 2000	11668019, Thermo Fisher	
Puromycin	A28560100, VWR	
QuickChange Site-Directed Mutagenesis kit	Agilent Technologies	
Roti^R^Histfix	P087.1, Carl Roth	
PVDF membrane	T831.1, Carl Roth	
I-Block^™^ powder	T2015, Applied Biosystems, Thermo Fisher	
Dynabeads™ Protein G	10004D, Invitrogen	
Dynabeads^™^ Protein A	10001D, Invitrogen	
Streptavidin Sepharose High Performance beads	Cytiva 17–5113-01, Merck	
EZ-Link^™^-Sulfo-NHS-LC-biotin	21335, Thermo Fischer	
Click-IT™ ManNAz Metabolic Glycoprotein Labeling Reagent	C33366, Invitrogen	
DBCO-Sulfo-Biotin	Jena Bioscience	
Concanavalin A agarose beads	C7555, Sigma-Aldrich	
Methyl-α-D-mannopyranoside	M6882, Sigma-Aldrich	
Click-iT^™^ EdU Cell Proliferation Kit for Imaging Alexa Fluor^™^ 647 dye	C10337, Invitrogen^™^	
Fluoromount^™^ Aqueous Mounting Medium	F4680, Sigma-Aldrich	
Transferrin from Human Serum Alexa Fluor^TM^647 Conjugate	T23366, Thermo Fisher	1:200

**Table:** 

Plasmids	Source	
pCMV3-IER3IP1 NM_016097.4	HG17098-UT, Sino Biological	
mCherry-C1-Sec24C	[16]	
pTT3-Unc5B-Flag	Addgene #72195 [17]	
pcDNA4 HisMax-V5-GFP-RRBP1 (p180)	Addgene #92150 [18]	
Str-STIM1-NN_ST-SBP-mCherry	Addgene #65263 [19]	
pcDNA5/FRT/TO V5	Addgene #19445 [20]	
PX458	Addgene #48139 [21]	
PX459	Addgene #48139 [21]	
pplss-mRFP-KDEL	[22]	
Sar1b-H79G	Dr. Rainer Pepperkok	
pEGFP-N1	Clontech, Takara Bio	
pSpCas9 BB-2A-GFP PX458 sgRNA_1	Genescript	
pSpCas9 BB-2A-Puro PX459 sgRNA_5	Genescript	
SF-LV-shLuciferase-mirE	Dr. Lenhard Rudolf [23]	
KDELR1_pEGFP-N1	Dr. Jorge Cancino [24]	
KDELR2_pEGFP-N1	Dr. Jorge Cancino [24]	
KDELR3_pEGFP-N1	Dr. Jorge Cancino [24]	
EGFP-ERGIC53	[25]	
pcDNA3.1 FGFR5-V5	Dr. Pavel Kejci [26]	
pCMV-myc-Sema4D	Addgene #51599 [27]	

**Table:** 

Primer name	Primer sequence	Source
U6 FWD	GAGGGCCTATTTCCCATGATTCC	Eurofins Genomics
FWD_IER3IP1_sg1_2	GAGAAATCGCTTGGACTTCG	Eurofins Genomics
REV_IER3IP1_sg1_3	CTGTTGAGCCCAAACCTGAT	Eurofins Genomics
FWD_Deletion	GAGAAATCGCTTGGACTTCG	Eurofins Genomics
REV_Deletion	CTGGCATGTCCTCTTCTGAG	Eurofins Genomics
FWD_Non-deletion	AAAACGAGTTGGGTGTGGAG	Eurofins Genomics
REV_Non-deletion	CTTCCATCAGAAGGGCAGAG	Eurofins Genomics
FWD pCMV3-hIER3IP1 V21G	GCGTCAACGCCATCGCAGGTCTGCACGAGGAGCGATTCC	Eurofins Genomics
REV pCMV3-hIER3IP1 V21G	GGAATCGCTCCTCGTGCAGACCTGCGATGGCGTTGACGC	Eurofins Genomics
FWD pCMV3-hIER3IP1 T79del	GCAGTGCTGCACGAGGAGCGATCCTCAAGAACATTGGCTGG	Eurofins Genomics
REV pCMV3-hIER3IP1 T79 del	CCAGCCAATGTTCTTGAGGATCGCTCCTCGTGCAGCACTGC	Eurofins Genomics
FWD pCMV3-hIER3IP1 L78P_2	CCATTGATAATAGTAAACTCAATTGCAATTGTGTTACCTTTATTATTTGGATGAATCTAGAGCGGCCGCCGAATTCGG	Eurofins Genomics
REV pCMV3-hIER3IP1 L78P_2	CCGAATTCGGCGGCCGCTCTAGATTCATCCAAATAATAAAGGTAACACAATTGCAATTGAGTTTACTATTATCAATGG	Eurofins Genomics
FWD_ P.T79D _mRNA _1	TTGCCTTTCTCTCCACAGGT	Eurofins Genomics
REV_ P.T79D_mRNA_1	TCTCCAAATCCACCAATTCC	Eurofins Genomics
FWD_EGFP-Ct	CAT GGT CCT GCT GGA GTT CGT G	Eurofins Genomics
FWD_Gibson_5mirE-Xhol	TTC GAC TTC TTA ACC CAA CAG AAG GCT CGA GAA GGT ATA TTG CTG TTG ACA GTG AGC G	IDT
REV_Gibson_3mirE-EcoR1	TTT AGT AAA CAA GAT AAT TGC TCG AAT TCT AGC CCC TTG AAG TCC GAG GCA GTA GGC	IDT

## Cell culture

HeLa Kyoto (RRID:CVCL_1922) and neuroblastoma SH-SY5Y (RRID:CVCL_0019) cells were grown in Dulbecco’s Modified Eagle Medium (1x) + Glutamax^™^ (61,965–026, Gibco, Thermo Fisher Scientific) with 10% Fetal Bovine Serum (F7524, Sigma-Aldrich) and 1 × Penicilin-Streptomycin (P0781, Sigma-Aldrich), and incubated at 37ºC and 5% CO_2_. Cells were detached using Trypsin–EDTA (25,300,054, Gibco) and washed with 1xPBS.

## Transfection

For transient transfection, 24 h after seeding, cells were incubated with DNA and Lipofectamine^™^ 2000 (11,668,019, Thermo Fisher), according to the manufacturer’s protocol. To obtain cell lines stably expressing IER3IP1 variants, KO1 IER3IP1 cells were plated in 6-well plates and transfected with 2.4 µg DNA and 7 µl of Lipofectamine per well. After 24 h, 200 µg/ml hygromicin B were added to each well, and 72 h later, 10 cells/ml were transferred to 10 cm plates. After 10–14 days, monoclonal cell colonies were picked and transferred to 24-well plates. Clones were analyzed for IER3IP1 expression and localization by immunoblot and immunofluorescence microscopy, and suitable clones were further grown with 200 µg/ml hygromicin B.

*siRNA transfection*: all siRNAs were from Dharmacon Reagents (HorizonDiscovery, UK).

siNon: ON-TARGETplus Non-targeting Control Pool.

siIER3IP1: On target plus Human IER3IP1 (51,124) siRNA SMART pool (L-018948–01-0020): GCUAAUGAACCUUAUUCGA, UUGGAGAAGAGCCGGGAAU, CCAUGAUGUGAGUGGUUAU, AUGAGAGUGCCAUUGAUAA.

HeLa cells were transfected with 25 nM of siRNA and DharmaFECT Transfection Reagent (HorizonDiscovery), and SHSY5Y cells with 50 nM of siRNA and Lipofectamine™ RNAiMAX (13,778,075, Thermo Fisher), as recommended.

## CRISPR-Cas9 knockout

sgRNAs were designed using CHOPCHOP [28]: sgRNA_1 CGCCATCGCAGTGCTGCACG (targeting exon 1) and sgRNA_5 ATTGAGTTTACTATTATCAA (targeting exon 3). The two sgRNAs were cloned in pSpCas9 BB-2A-GFP PX458 and pSpCas9 BB-2A-Puro PX459 sgRNA_5, respectively, by Genescript, and analyzed by sequencing with the U6 FWD primer. HeLa cells were transfected with Lipofectamine 2000, and pSpCas9 BB-2A-GFP PX458 sgRNA_1 and pSpCas9 BB-2A-Puro PX459 sgRNA_5 to knockout IER3IP1, or the control vectors PX458 and PX459 to obtain a control cell line. After 1 day, we sorted the top 5% GFP-positive cells using fluorescence activated cell sorting (FACS). Sorted cells were resuspended in medium supplemented with 1.5 µg/ml Puromycin (A2856.0100, VWR), and plated in 6-well plates (100,000 cells/well). After 24 h, cells were washed twice with 1xPBS, grown in complete medium for 1 more day, and then transferred to either 10 cm plates (100 cells/plate) or to 96 well plates (0.5 cells in 200 µl per well), and grown in complete medium for 2 weeks, until colonies formed. Clonal colonies were selected and transferred to 24-well plates and then to 6-well plates.

The presence of INDEL mutations was analyzed by sequencing (see Suppl. Fig.S1). After genomic DNA isolation, the targeted region was amplified by PCR using specific primers and GoTaq polymerase (Promega), according to the manufacturer’s protocol. The entire IER3IP1 gene was amplified with FWD_Deletion and REV_Deletion primers, and a region downstream of the sgRNA_1 targeted region with FWD_Non-deletion and REV_Non-deletion primers. The region targeted by sg_RNA_1 was amplified with the FWD_IER3IP1_sg1_2 and REV_IER3IP1_sg1_3 primers, and Sanger sequences of these amplicons were analyzed using the Inference of CRISPR Edits tool (ICE; ice.synthesgo.com). The KO2 clone was selected from a total of 10 clones as having the highest (93%) ICE KO score. The KO1 clone had a large deletion that comprised the entire IER3IP1 gene. To confirm the KO of IER3IP1 we analyzed clones by immunoblot and immunofluorescence. Both the KO1 and the KO2 clones displayed morphologies and growth rates comparable to control (Cas9-transfected) cells. Images were created using the SnapGene software (Suppl. Fig.S1).

Site-Directed Mutagenesis was done using the QuickChange Site-Directed Mutagenesis kit (Agilent Technologies, CA, USA), according to the suggested protocol. The primers used were, for pCMV3-IER3IP1 V21G: FWD pCMV3-hIER3IP1 V21G, REV pCMV3-hIER3IP1 V21G, for pCMV3-IER3IP1 L78P: FWD pCMV3-hIER3IP1 L78P_2, REV pCMV3-hIER3IP1 L78P_2, and for pCMV3-IER3IP1 T79: FWD pCMV3-hIER3IP1 T79del, REV pCMV3-hIER3IP1 T79 del.

## Immunocytochemistry and microscopy

Cells grown on No.1 glass coverslips (0111550, Paul Marienfeld GmbH, Germany) were treated as indicated, then washed with 1xPBS, fixed with Roti^R^Histfix (P087.1, Carl Roth, Germany) (20 min at room temperature), permeabilized with 0.2% Triton X-100 in PBS (5 min), and blocked with a solution containing 1 ml FCS, 1 g BSA, 0.1 g fish gelatin in 100 ml 1xPBS for 20 min. Cells were then incubated with the primary antibodies diluted in blocking solution (1 h, room temperature), washed 3 × 5 min with PBS, then labeled with secondary antibodies (20 min, room temperature), washed 3 × 5 min with PBS, and mounted on microscope slides (AA00000112E01MNZ10, Epredia, Germany) using Mowiol 4–88 with Hoechst 33,342 (1:1000). Images were acquired using an Imager Z.2 equipped with an Apotome.2 and Axiocam 702, with a Plan-Apochromat 63x/1.4 Oil DIC M27 objective (Carl Zeiss AG, Germany).

For live cell imaging, cells were plated in 8-well Chambered Coverglass w/non-removable wells No. 1 (155,411, Thermo Scientific^™^ Nunc ^™^ Lab-Tek ^™^, Thermo Fisher). Images were acquired using an Axio Observer Z1/7 equipped with an Apotome.2 and Axiocam 702 Mono, with a Plan-Apochromat 63x/1.40 Oil DIC M27 objective (Carl Zeiss AG).

For high-throughput imaging, cells grown in an Eppendorf Cell Imaging Plate, 96-well (Eppendorf AG, Germany) were either incubated with MR-F2 (1:250) from a Magic Red^™^ Cathepsin L kit and 5 µg/ml Hoechst 33,342 (60 min, 37ºC), or fixed and labeled with anti-LAMP1 and Hoechst 33,342. Cells were imaged with an ImageXpress Micro Confocal microscope equipped with a 40x, NA = 0.95 CFI Plan Apo Lambda air immersion objective, temperature, and humidity control, Air/CO2 gas mixer from OKOLAB, Italy) and MetaXpress High-Content Image Acquisition and Analysis v.6.7.2.290 (64-bit) software (Molecular Devices, CA). For LAMP1, Z-stacks were acquired and MaxProjections were analyzed.

## RUSH assay

Cells grown on coverslips were transfected with Str-STIM1-NN_ST-SBP-mCherry (ST-mCherry) [19] and Lipofectamine. After 24 h, 40 µM biotin was added into the medium, and cells were incubated for the indicated times at 37ºC, then fixed and immunostained. The mean fluorescence intensity of the mCherry signal was measured in the Golgi, GM130-positive, region and in the entire cell, using Fiji [29]. Ratios between the Golgi and the total cellular average intensities were calculated using Excel.

## Transferrin uptake

Cells were starved in DMEM with 0.5% BSA for 1 h at 37° C, then incubated with 25 µg/ml Tfn-Alexa 647 (1:200) for in 10 min at 37° C, fixed and analyzed by fluorescence microscopy.

## ER shape analysis

Cells grown in 8-well chambers were transfected with GFP-p180 and Lipofectamine 2000, and live-cell imaged after 1 day. Fiji was used to measure, for individual cells, the GFP-p180-positive area/cell (after thresholding using the Fiji “Moments” algorithm) and the total cell area, and the ratio between the two areas was calculated per cell.

To measure the number of objects per cell for the different markers analyzed, as well as the co-localization between GM130 and ST6GAL1 (object-based Pearson’s correlation coefficient), images were processed with Fiji, and object thresholding, detection and measurement per image were performed with Cell Profiler v3 or v4 *(*www.cellprofiler.org*)*[30].

## IncuCyte growth curve analysis

Cells were plated in Nunc^™^ MicroWell^™^ 96-Well, Nunclon Delta-Treated, Flat-Bottom Microplate (167,008, Thermo Fisher) (5000 cells/well) and imaged every 3 h for the indicated times, using the IncuCyte S3 Live-Cell Analysis System (Sartorius, Germany) with an IncuCyte S3 microscope (4647, Essen BioScience) and a 10 × objective. In each well, images were acquired at four distinct positions. Cell confluence (i.e., the area occupied by cells) was measured, and the average confluence per well was calculated with the IncuCyte S3 software.

## Transmission electron microscopy

Cells were fixed with Karnovsky fixative (2% paraformaldehyde, 3% glutaraldehyde in 0.1 M cacodylate buffer, pH 7.3), for 3 h at room temperature, then overnight at 4 °C. After 5 × 15 min washes with cacodylate buffer, cells were incubated (protected from light) with 2% osmium tetroxide + 1% potassium hexacyanidoferrate (II) in 0.1 M cacodylate buffer, at 4 °C for 2 h, and then washed 3 × 15 min with cacodylate buffer. Fixed cells were scrapped and centrifuged (2000 × rpm using a table centrifuge, room temperature), then embedded in 3% agar, and cut into small pieces with a razor blade. Using a tissue processor (Leica, Germany) these cuts were washed 3 × 15 min with cacodylate buffer, 3 × 15 min with distilled water, dehydrated by incubating with acetone with increasing concentrations: 30, 50, 70, 90, 95 and 3 × 100% (30 min each), and stained with 1% uranyl acetate in 50% acetone. Sample infiltration was performed with epoxy resin (Glycid ether 100, #21,045 SERVA Electrophoresis GmbH, Germany): a mixture of acetone:resin 3:1 (45 min), 1:1 (45 min), and 1:3 (45 min), then 3 × 2 h pure epoxy resin, and 1 × 2 h epoxy resin + accelerator dimethylbenzylamine. Samples were embedded in flat moulds, allowed to polymerize at 60 °C for 48 h, then trimmed with a Reichert UltraTrim (Leica). Semithin Sects. (0.5 µm) were labeled with Azure staining [31], then ultrathin Sects. (55 nm) made with an ultramicrotome “Reichert Ultracut S” (Leica) were placed onto copper slot grids coated with a Formvar/Carbon layer. Images were obtained with a transmission electron microscope JEM 1400 (JEOL, MA) with a CCD camera ‘Orius SC 1000A’ (GATAN, CA), an acceleration voltage of 80 kV, and GATAN MICROSCOPY SUITE 2.31.734.0 software.

## RNA Expression analysis

Cells were scraped and collected in PBS, then centrifuged (1500 × g, 2 min, 4ºC). Pellets were lysed, RNA was extracted using the NucleoSpin RNA, mini kit for RNA purification (740,955.50, Machery-Nagel, Germany). cDNA was synthesized using the qScript cDNA Synthesis Kit (95,047, Quanta BioSciences, MA), as indicated by the manufacturer, and analyzed by PCR using the FWD_ P.T79D _mRNA _1 and REV_ P.T79D_mRNA_1 primers.

## SDS-PAGE and immunoblotting

Cells grown to confluence in 6-well or 10 cm plates were washed with ice-cold PBS, detached with a cell scraper in lysis buffer (50 mM Tris–HCl pH 7.6, 150 mM NaCl, 2 mM EDTA, 1% NP-40 and protease inhibitors) or CHAPSO buffer (1% CHAPSO, 150 mM NaCl, 5 mM EDTA, 50 mM Tris pH 7.6 and protease inhibitors), lysed on ice for 30 min, then centrifuged (500xg, 5 min, 4 °C). Supernatants were collected and proteins were denatured by boiling at 90 °C for 5 min with sample buffer. Proteins were separated by SDS-PAGE, transferred to Immobilon-P PVDF membranes (T831.1, Carl Roth). Page Ruler™ Plus Pre-stained Protein Ladder (26,619, Thermo Fisher) was used as protein size standard. Membranes were incubated in blocking solution: 1 g I-Block^™^ powder (T2015, Applied Biosystems, Thermo Fisher), 0.1% Tween-20 (9127.2, Carl Roth) in 500 ml of 1xPBS, with primary antibodies diluted in blocking solution (1 h at room temperature or overnight at 4 °C), washed 3 × 5 min with 1xTBS-Tween, incubated with secondary antibodies (30 min, room temperature) and washed 3 × 5 min with 1xTBS-Tween.

## Immunoprecipitation

Equal numbers of cells were plated in 10 cm plates and transfected the next day with the indicated plasmids and Lipofectamine 2000. After 24 h, cells were detached, collected in CHAPSO buffer, lyzed 30 min on ice, then centrifuged 13 000 × g, 40 min at 4ºC. Supernatants were transferred to low binding Protein LoBind Tube 1.5. ml (022431081, Eppendorf), and incubated with the indicated antibodies (10 µl for anti-IER3IP1, 5 µl for the other antibodies), in the cold room with rotation, overnight. Next day, pre-washed 80 µl of Dynabeads™ Protein G (10004D, Invitrogen) were added to each tube, incubated in the cold room with rotation, for 30 min. Beads were then washed 5 × 1 ml lysis buffer, transferred to a new tube before the last wash, then resuspended in sample buffer, and analyzed by SDS-PAGE and immunoblot.

## Immunoprecipitation from culture medium

Equal numbers of cells were plated in 4 × 10 cm plates for each condition. After 24 h, medium was replaced with 5 ml of fresh medium, and cells were incubated at 37ºC for 2 more days. Media from plates corresponding to the same experimental condition were combined, and volumes were adjusted to correspond to equal numbers of cells. After addition of protease inhibitors, media were filtered through a 0.45 µm Rotilabo PVDF filter (P667.1, Carl Roth), loaded onto a Vivaspin 20, 30 kDa Diafilter (VS2022, Sartorius AG) and concentrated by centrifugation (4000 × g, 4ºC, 80 min) to a volume of 0.5 ml of retentate. Retentates were transferred to low binding tubes, 2 µl of anti-BiP were added to each tube, followed by an incubation at 4ºC, with rotation, overnight. The next day, 100 µl of prewashed Dynabeads™ Protein A (10001D, Invitrogen) were added to each tube, followed by a 30 min incubation at 4ºC, with rotation. Beads were further washed 5 × 1 ml lysis buffer and resuspended in Sample buffer. Cells were lysed in lysis buffer (50 mM Tris–HCl pH 7.6, 150 mM NaCl, 2 mM EDTA, 1% NP-40 and protease inhibitors) and analyzed by immunoblot.

## Glycosylation assays

Lysates from HeLa cells transfected with Unc5B-FLAG and Lipofectamine^™^ 2000 were treated with EndoH (P0702, New England BioLabs) or PNGaseF (P0704, New England BioLabs), at 37°C for 16 h, as indicated by the manufacturer, then analyzed by immunoblot.

Autophagy analysis [32]. Cells grown in 6-well plates were incubated with growth medium containing DMSO or 50 µg/ml chloroquine (Sigma-Aldrich, C6628), or 500 nM torin 1 (Bio-Techne/Tocris**)** in EBSS starvation medium, for 5 h, then lysed and analyzed by SDS-PAGE and immunoblot.

## ER stress analysis

Cells grown in 6-well plates were treated with 1 µM thapsigargin or an equal volume of control DMSO for the indicated times, lysed and analyzed by immunoblot.

## Surface proteome and secretome analyses

### Acetylation of beads

Streptavidin Sepharose High Performance beads (Cytiva 17–5113-01, Merck) were equilibrated in PBS and lysine-acetylated using 20 mM sulpho-NHS-acetate for 1 h at room temperature. Sulpho-NHS-acetate was quenched by adding 1 M Tris pH 7.5 and beads were extensively washed with PBS.

### Surface biotinylation

Cells were grown in 10 cm plates for 2 days, then processed on ice. Each plate was washed with 3 × 5 min × 5 ml of ice-cold PBS with Ca^2+^ and Mg^2+^, then incubated with 3 ml of 0.5 mg/ml of EZ-Link™-Sulfo-NHS-LC-biotin (21,335, Thermo Fischer) for 30 min, washed 4 × 15 min × 5 ml of 20 mM glycin in PBS, then with 5 ml PBS with Ca^2+^ and Mg^2+^, and collected in lysis buffer (50 mM Tris–HCl pH 7.6, 150 mM NaCl, 2 mM EDTA, 1% NP-40 and protease inhibitors). Lysates were incubated on ice for 30 min, centrifuged at 6000 × g, 4ºC, for 6 min. For each sample, 160 µl of acetylated beads (50% suspension) were equilibrated in lysis buffer before adding them to lysates overnight, at 4 °C, rotating at 15 rpm. For immunoblot analysis, beads were washed 1 × 1 ml Buffer 1 (50 mM Tris HCl pH 7.6, 325 mM NaCl, 2 mM EDTA, 0.2% NP-40), 5 × 1 ml Buffer 2 (50 mM Tris–HCl pH 7.6, 150 mM NaCl, 2 mM EDTA, 1% NP-40, 1% SDS) and 5 × 1 ml Buffer 3 (50 mM Tris–HCl pH 7.6, 150 mM NaCl, 2 mM EDTA), then resuspended in sample buffer with 3 mM biotin, and analyzed by immunoblot. For mass spectrometry analysis, beads were washed with 1 ml of Buffer 1, 4 ml of Buffer 2, and 5 ml of Buffer 3. Beads were finally washed 5 times with 600 µl Wash Buffer 2 (50 mM Ammonium Bicarbonate/AmBic pH 8.0).

Secretome protein enrichment with click sugars [33]. To label glycoproteins, cells (5 × 10 cm plates/condition) were incubated with medium with 62.5 µM Click-IT™ ManNAz Metabolic Glycoprotein Labeling Reagent (C33366, Invitrogen) for 48 h. Cell growth medium was supplemented with EDTA-free protease inhibitors and filtered (0.45 µm). Volumes were adjusted to correspond to equal cell numbers and loaded onto Vivaspin 20, 30 kDa Diafilter and concentrated by centrifugation (4000 × g, 4ºC, 80 min) to 0.5 ml, then washed 2 × 15 ml PBS at 4ºC. The volume of each sample was adjusted to 1 ml and incubated with 100 µM DBCO-Sulfo-Biotin (Jena Bioscience, Germany) overnight at 4 °C, then washed 3 × 15 ml PBS. Concanavalin A agarose beads (C7555, Sigma-Aldrich) were equilibrated by washing 2 × 1 ml Binding buffer (5 mM MgCl_2_, 5 mM MnCl_2_, 5 mM CaCl_2_, 500 mM NaCl in 20 mM Tris–HCL pH 7.5). 300 µl of beads were added to each sample together with 1 ml Binding buffer in low binding tubes, then incubated at 4ºC, with rotation, for 2 h. Beads were washed 3 × 1 ml Binding buffer, and proteins were eluted twice with 500 µl Elution buffer (500 mM Methyl-α-D-mannopyranoside (M6882, Sigma-Aldrich), 10 mM EDTA, 20 mM Tris–HCL pH 7.5) for 30 min at 4 °C. Combined eluates were passed through Pierce™ Spin Columns (69,705, Thermo Fisher) and then divided into two low binding tubes. 0.5 ml of 2% SDS in PBS and 300 µl of acetylated streptavidin beads were added to each tube, and samples were incubated at 4 °C with rotation at 15 rpm, overnight. Afterwards, samples were centrifuged (2000 × g, 4 °C, 5 min), and beads were resuspended in PBS, transferred to a Spin Column, washed 1 × 1 ml PBS and 3 × 1 ml Wash Buffer 1 (30 mM AmBic, 3 M Urea), and finally 5 × 600 µl Wash Buffer 2 (50 mM AmBic pH 8.0).

## Mass spectrometry analysis

### On-bead digest for pulldowns

Beads were transferred to a new tube using Wash Buffer 2, centrifuged at 2000 × g for 5 min and the supernatant discarded. Beads were resuspended in 200 μl Wash Buffer 2 and 1 µg LysC added. After an incubation overnight at 37 °C, peptides were eluted two times with 150 µl Wash Buffer 2. Elutions were further digested with 0.5 µg trypsin for 3 h at 37 °C. The day after, digests were acidified by the addition of trifluoroacidic acid (TFA) to a final concentration of 1% (v/v), then desalted with Waters Oasis^®^ HLB µElution Plate 30 µm (Waters Corporation, MA) under a soft vacuum, following the manufacturer’s instructions. Briefly, columns were conditioned with 3 × 100 µL solvent B (80% (v/v) acetonitrile; 0.05% (v/v) formic acid) and equilibrated with 3 × 100 µL solvent A (0.05% (v/v) formic acid in Milli-Q water). Samples were loaded, washed 3 times with 100 µL solvent A, and then eluted into 0.2 mL PCR tubes with solvent B. Samples were dried with a speed vacuum centrifuge and stored at − 20 °C until LC–MS analysis. Data Acquisition and Processing for DIA Samples. Prior to analysis, samples were reconstituted in MS Buffer (5% acetonitrile, 95% Milli-Q water, with 0.1% formic acid) and spiked with iRT peptides (Biognosys AG, Switzerland). Peptides were separated in trap/elute mode using the nanoAcquity MClass Ultra-High Performance Liquid Chromatography system (Waters, Waters Corporation, Milford, MA) equipped with a trapping (nanoAcquity Symmetry C18, 5 μm, 180 μm × 20 mm) and an analytical column (nanoAcquity BEH C18, 1.7 μm, 75 μm × 250 mm). Solvent A was water and 0.1% formic acid, and solvent B was acetonitrile and 0.1% formic acid. 1 µl of the sample (∼1 μg on column) were loaded with a constant flow of solvent A at 5 μl/min onto the trapping column. Trapping time was 6 min. Peptides were eluted via the analytical column with a constant flow of 0.3 μl/min. During the elution, the percentage of solvent B increased in a nonlinear fashion from 0–40% in 120 min. Total run time was 145 min, including equilibration and conditioning. The LC was coupled to an Orbitrap Exploris 480 (Thermo Fisher Scientific, Bremen, Germany) using the Proxeon nanospray source. The peptides were introduced into the mass spectrometer via a Pico-Tip Emitter 360-μm outer diameter × 20-μm inner diameter, 10-μm tip (New Objective, MA), heated at 300 °C, and a spray voltage of 2.2 kV was applied. The capillary temperature was set at 300 °C. The radio frequency ion funnel was set to 30%. For DIA data acquisition, full scan mass spectrometry (MS) spectra with mass range 350–1650 m/z were acquired in profile mode in the Orbitrap with resolution of 120,000 FWHM. The default charge state was set to 3 + . The filling time was set at maximum of 60 ms with limitation of 3 × 10^6^ ions. DIA scans were acquired with 40 mass window segments of differing widths across the MS1 mass range. Higher collisional dissociation fragmentation (stepped normalized collision energy; 25, 27.5, and 30%) was applied and MS/MS spectra were acquired with a resolution of 30,000 FWHM with a fixed first mass of 200 m/z after accumulation of 3 × 10^6^ ions or after filling time of 35 ms (whichever occurred first). Data were acquired in profile mode. For data acquisition and processing of the raw data Xcalibur 4.3 (Thermo Fisher Scientific) and Tune version 2.0 were used. Data Analysis for DIA Samples. Spectronaut (v. 16, Biognosys AG). DIA data were then uploaded and searched against this spectral library in Spectronaut. Data were searched with the following modifications: Oxidation (M), Acetyl (Protein N-term). A maximum of 2 missed cleavages for trypsin and 5 variable modifications were allowed. The identifications were filtered to satisfy FDR of 1% on peptide and protein level. Relative quantification was performed in Spectronaut for each paired comparison using the replicate samples from each condition. The data were then exported. To select significant proteins, a log2FC cutoff of 0.58 and a Q_value_ < 0.05 were defined.

i^3^ Neurons were obtained from human induced pluripotent stem cells stably expressing the doxicyclin inducible neurogenin-2 (iPSC) and pC13N-dCas9-BFP-KRAB (CRISPRi i3N iPSCs), as described [34, 35]. Shortly, iPSCs were maintained in Essential 8^™^ Flex Medium Kit (A2858501, Thermo Fisher) with Rock inhibitor (Y-27632, #1254, bio-techne/TOCRIS), then pre-differentiated for 3 days on Corning® Matrigel^®^ hESC-Qualified Matrix (CLS354277, Merck)-coated 10 cm plates in Induction Medium: DMEM/F12 (11,330,032, Gibco), Non-essential amino acids (11,140,050, Gibco), L-Glutamine (25,030,081, Gibco), N2 supplement (17,502,048, Gibco) with Rock inhibitor (day 1), and 2 mg/ml Doxycycline (D9891, Sigma-Aldrich) (days 1–3). Cells were dissociated on day 3 with StemPro™ Accutase™ Cell Dissociation Reagent (A1110501, Thermo Fisher). Cells were then transferred to plates coated with poly-L-ornithine solution (P4957, Sigma-Aldrich) diluted 1/10 in borate buffer (100 mM boric acid, 25 mM sodium tetraborate, 75 mM sodium chloride, pH 8.4), and grown in differentiation medium: Neurobasal Medium (10,888,022, Gibco), B27 (17,504,044, Gibco), 10 µg/ml BDNF (450–02, Peprotech), 10 µg/ml NT-3 (Peprotech, 450–03), 1 mg/ml Laminin (L2020, Sigma Aldrich) and 2 mg/ml Doxycycline, for the indicated times. Medium was half-changed every 3–4 days during the first week of differentiation, and once a week afterwards.

IER3IP1 KO iPSC cells were obtained using the CRISPR Cas9 system, as described for HeLa cells, above. Cells were transfected in 12 well plates, with 1 µg of DNA (pSpCas9 BB-2A-GFP PX458 and pSpCas9 BB-2A-Puro PX459 sgRNA_5) and 3 µl of Lipofectamine Stem Transfection Reagent (STEM00015, Thermo Fisher) per well. EGFP-positive cells were single-cell sorted in 96-well plates using FACS. Colonies were further selected and validated by Sanger sequencing and Western blot.

*Analysis of i*^*3*^* neuron secretome*: I^3^ neurons were differentiated for 12 days, then the medium was replaced with fresh differentiation medium (5 ml/plate), and cells were allowed to secrete for 48 h at 37° C. Collected medium was centrifuged (4 ℃, 4500 × g, 3–6 h) through Vivaspin columns until its volume was reduced to ~ 500 µl. BioMag^®^ Plus Concanavalin A beads (86,057, Polysciences, PA) were pre-washed and resuspended in 1 ml of Binding Buffer (1 × PBS, 1 mM MgCl2, 1 mM MnCl2, 1 mM CaCl2, pH 7.4). To immunoprecipitate glycoproteins, concentrated medium was incubated with 1 ml of Concanavalin A beads for 30 min at room temperature, with rotation. Beads were then washed 3 × 5 min with 0.5 ml Wash Buffer (1 × PBS, 1 mM MgCl2, 1 mM MnCl2, 1 mM CaCl2, 1% Tween^®^ 20, pH 7.4), and proteins were eluted by incubating them 2 × 250 µl of Elution Buffer (5 mM TrisHCl pH 8.0, 0.15 M NaCl, 0.05% SDS, 1 M Glucose), 30 min at room temperature. For proteomics analysis, 200 μl of concentrated medium were transferred to tubes and 800 μl of ice-cold 100% acetone were added. After overnight precipitation at −20 °C, samples were then centrifuged at 14 000 × rpm for 30 min, 4 °C. After removal of the supernatant, the precipitates were washed twice with 300 µL of a solution of ice cold 80% acetone. After addition of each wash solution, the samples were vortexed and centrifuged again for 10 min at 4 °C. The pellets were then allowed to air-dry before being alkylated with 15 mM iodoacetamide for 30 min at room temperature, in the dark. Samples were acidified with 2.5% phosphoric acid, and seven times the sample volume of S-trap binding buffer was added (100 mM TEAB, 90% methanol). Samples were bound on 96-well S-trap micro plate (Protifi, NY) and washed three times with binding buffer. Trypsin in 50 mM TEAB (pH 8.5) was added to the samples (1 µg per sample) and incubated for 1 h at 47 °C. The samples were eluted in three steps with 50 mM TEAB pH 8.5, elution buffer 1 (0.2% formic acid in water) and elution buffer 2 (50% acetonitrile and 0.2% formic acid). The eluates were dried using a speed vacuum centrifuge (Eppendorf Concentrator Plus, Eppendorf AG, Germany) and stored at −20° C.

## LC–MS Data independent analysis (DIA)

Samples were reconstituted in in MS Buffer (5% acetonitrile, 95% Milli-Q water, with 0.1% formic acid) and loaded on Evotips (Evosep, Odense, Denmark) according to the manufacturer’s instructions. In short, Evotips were first washed with Evosep buffer B (acetonitrile, 0.1% formic acid), conditioned with 100% isopropanol and equilibrated with Evosep buffer A. Afterwards, the samples were loaded on the Evotips and washed with Evosep buffer A. The loaded Evotips were topped up with buffer A and stored until the measurement. Peptides were separated using the Evosep One system (Evosep) equipped with a 15 cm × 150 μm i.d. packed with a 1.9 μm Reprosil-Pur C18 bead column (Evosep Endurance, EV-1106, PepSep, Marslev, Denmark). The samples were run with a pre-programmed proprietary Evosep gradient of 44 min (30 samples per day) using water and 0.1% formic acid and solvent B acetonitrile and 0.1% formic acid as solvents. The LC was coupled to an Orbitrap Exploris 480 using PepSep Sprayers and a Proxeon nanospray source. The peptides were introduced into the mass spectrometer via a PepSep Emitter 360-μm outer diameter × 20-μm inner diameter, heated at 300 °C, and a spray voltage of 2 kV was applied. The injection capillary temperature was set at 300 °C. The radio frequency ion funnel was set to 30%. For DIA data acquisition, full scan mass spectrometry (MS) spectra with a mass range of 350–1650 m/z were acquired in profile mode in the Orbitrap with a resolution of 120,000 FWHM. The default charge state was set to 2 + , and the filling time was set at a maximum of 20 ms with a limitation of 3 × 10^6^ ions. DIA scans were acquired with 40 mass window segments of differing widths across the MS1 mass range. Higher collisional dissociation fragmentation (normalized collision energy 30%) was applied, and MS/MS spectra were acquired with a resolution of 30,000 FWHM with a fixed first mass of 200 m/z after accumulation of 1 × 10^6^ ions or after filling time of 45 ms (whichever occurred first). Data were acquired in profile mode. For data acquisition and processing of the raw data, Xcalibur 4.4 and Tune version 4.0 were used.

## Data analysis

DIA raw data were analyzed using the directDIA pipeline in Spectronaut v.18 with BGS settings besides the following parameters: Protein LFQ method = QUANT 2.0, Proteotypicity Filter = Only protein group specific, Major Group Quantity = Median peptide quantity, Minor Group Quantity = Median precursor quantity, Data Filtering = Qvalue, Normalizing strategy = Local Normalization. The data were searched against a species specific(*Homo sapiens*,v. 160,126, 20.186 entries), and a contaminants (247 entries) Swissprot database. The data were searched with the following variable modifications: Oxidation (M) and Acetyl (Protein N-term). A maximum of 2 missed cleavages for trypsin and 5 variable modifications were allowed. The identifications were filtered to satisfy FDR of 1% on peptide and protein level. Relative quantification was performed in Spectronaut for each paired comparison using the replicate samples from each condition. The data (candidate table) and data reports (protein quantities) were then exported, and further data analyses and visualization were performed with Rstudio using in-house pipelines and scripts. To select significant proteins, a log2FC cutoff of 0.58 and a q-value < 0.05 were defined.

## In utero electroporation

### Ier3ip1 shRNA cloning

97 bp shRNA ultramers were designed for the reference sequence NM_025409.3 using SPLASH RNA [36]. The top three SPLASH shRNAs were synthesized by IDT:

*NM_025409.3_235_v2*:TGCTGTTGACAGTGAGCGACAGGAATTAAATCTCAACTAATAGTGAAGCCACAGATGTATTAGTTGAGATTTAATTCCTGGTGCCTACTGCCTCGGA;

*NM_025409.3_1373_v2*:TGCTGTTGACAGTGAGCGCAAAGTTGACTTTTCATATTAATAGTGAAGCCACAGATGTATTAATATGAAAAGTCAACTTTATGCCTACTGCCTCGGA;

*NM_025409.3_1340_v2*:TGCTGTTGACAGTGAGCGCTGGCATCTTTCTGTATAGAAATAGTGAAGCCACAGATGTATTTCTATACAGAAAGATGCCATTGCCTACTGCCTCGGA.

For each shRNA, DNA fragments generated by PCR using GoTaqG2 polymerase (Promega) and the primers FWD_Gibson_5mirE-Xhol and REV_Gibson_3mirE-EcoR1 were cloned in the SF-LV-shRNA-mirE vector under the spleen-focus forming promoter (SFFV) [23, 37], using NEBuilder^®^ HiFi DNA Assembly Master Mix (New England Biolabs, MA), and 3 clones/shRNA were sequenced using the FWD_EGFP-Ct primer (Eurofins Genomics). To test knockdown efficiency, NIH3T3 cells, grown in the same conditions as HeLa cells, were transfected with one selected clone per shRNA and lipofectamine. EGFP-positive cells were sorted after 3 days and analyzed by immunoblot and immunofluorescence microscopy. shRNA NM_025409.3_235_v2 showed the highest knockdown efficiency and was selected for further experiments. A construct expressing shLuciferase (AGGAATTATAATGCTTATCTA) was used as a negative control.

In utero electroporation was performed as described [38]. Shortly, pregnant (E13.5) C57BL/6 J wildtype (Janvier) mice were anesthetized with isoflurane, and ~ 2 µl of 2 mg/ml of plasmid and 0.01% Fast Green were microinjected in the embryonic brains (lateral ventricle), followed by electroporation (6 pulses of 30 V and 5 ms) with an electroporator ECM830 Square Wave Electroporation System (BTX, MA). Surgical wounds were sutured after uterus repositioning, and mice were transferred to a new box when awake. After one day, 1 dose of 1 mg/kg of EdU was administered intraperitoneally, and, after one more day, animals were sacrificed by cervical dislocation. Animal procedures followed TVV16/2018 and were approved by local authorities.

Dissected brains were fixed in 4% paraformaldehyde overnight, then 40 µm coronal sections were obtained using a Microm HM 650 V Vibratome (Thermo Fisher). Sections were mounted on Superfrost^TM^Plus Adhesion Microscope Slides (J1800AMNZ, Epredia, Germany), dried 30 min at room temperature and washed with PBS (15 min), followed by microwave antigen retrieval with 10 mM sodium citrate buffer (pH 6.0) and washed 3 × 10 min with PBS. Sections were incubated with blocking buffer (5% normal goat serum, 1% BSA, 0.4% Triton X-100 in PBS) for 2 h at room temperature in a humid chamber, then with primary antibodies diluted in blocking solution (overnight at 4ºC). Next day, sections were washed 3 × 15 min with PBS, incubated with secondary antibodies diluted (1:1000) in blocking solution (2 h, room temperature), washed 3 × 15 min with PBS, incubated, where indicated, with Click-iT™ EdU Cell Proliferation Kit for Imaging Alexa Fluor™ 647 dye (Invitrogen™) for 30 min, washed for 5 min with PBS, then incubated with Hoechst (1:10 000) for 30 min and washed 3 × 15 min with PBS. Sections were then stained with 0.1% Sudan Black B in 70% ethanol for 20 min, washed 3 × 5 min with PBS-Tween (0,02%), then embedded in Fluoromount^™^ Aqueous Mounting Medium (F4680, Sigma-Aldrich) and covered with #1 coverslips (H878 and 1870.2, Carl Roth). Images were acquired using an Imager Z.2 equipped with an Apotome.2 and Axiocam 702, with a Plan-Apochromat 63x/1.4 Oil DIC M27 or a 20x/0.8 M27 objective (Carl Zeiss AG). Maximal intensity projections of serial Z-stacks were analyzed using Fiji.

Brain regions were defined based on nucleus morphology and density, and by labeling with specific markers: Tbr-2 is enriched the subventricular zone (SV), Sox2 in the ventricular zone (VZ), Ki67 labels progenitors in the VZ and SVZ. EdU-labeled cells display different densities and orientations in the VZ (perpendicular to the ventricular wall) and SVZ. The long axes of cells in the cortical plate (CP) are perpendicular to the surface, and cells have a higher density compared to cells in the subjacent region of the intermediate zone (IZ), which is located between the SVZ and CP. To characterize neuronal morphologies in the cortical plate, EGFP-positive cells were classified as (1) unbranched uni/bipolar, when the leading process was not branched, (2) branched bipolar, with a branched leading process or (3) complex, with a leading process displaying a more complex arborization [39]. For multipolar cells located in the intermediate zone, for each cell, the longest detectable neurite assignable to a cell body was measured using the “Measure” function in Fiji.

**Fig. 1:**
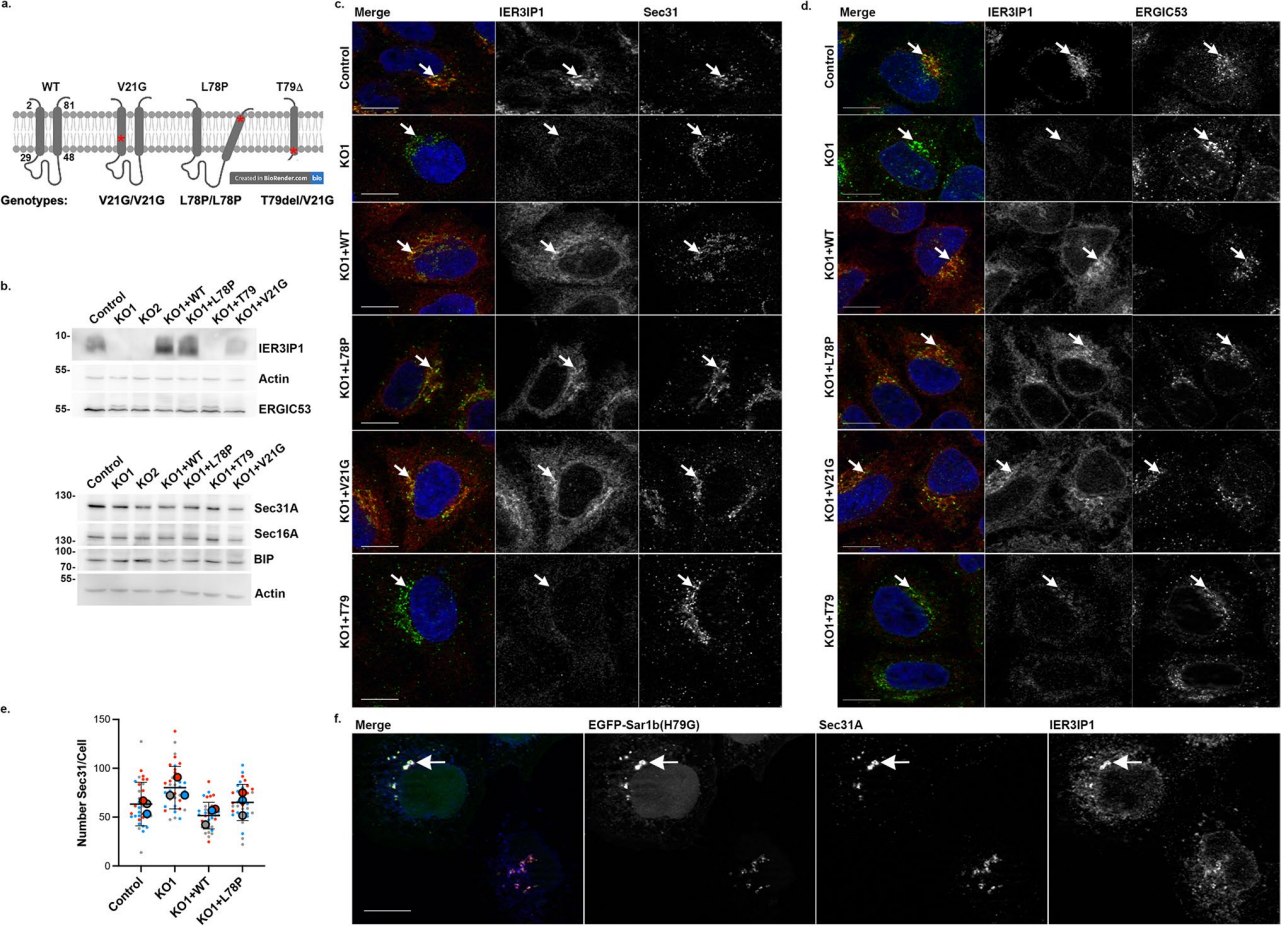
IER3IP1 is a protein of the early secretory pathway. a) Model of IER3IP1 structure. Numbers indicate first and last amino acids of the two predicted transmembrane domains (https://alphafold.ebi.ac.uk/entry/Q9Y5U9). The localization of the pathogenic mutations is indicated (red asterisks). b) *IER3IP1* KO and re-expression of *IER3IP1* cell line variants were analyzed by WB using anti-IER3IP1. Lysates were assessed for expression of the indicated proteins, with β-actin serving as loading control (n = 3 independent experiments). c, d) Indicated cells were fixed, co-labeled with anti-IER3IP1 (red) and (c) Sec31 (green) or (d) ERGIC53 (green), and Hoechst nuclear dye (blue), and analyzed by fluorescence microscopy. Arrows indicate overlap of IER3IP1 with the respective marker. e) The numbers of Sec31 objects per cell in cells processed as in (c) were analyzed using Cell Profiler. Values obtained from individual images are represented as small circles and mean values for independent experiments as filled circles (n = 3 independent experiments, mean ± SD, One-way Anova with Dunnett’s post-hoc test, n _Control_ = 506, n _KO1_ = 534, n _KO1+WT_ = 644, n _KO1+P.L78P_ = 493 cells). f) Cells were transfected with EGFP-Sar1b (H79G), fixed after 24 h, labeled with anti-IER3IP1 (blue) and Sec31A (red), and analyzed by fluorescence microscopy. Arrows indicate co-localization of the three proteins. Scale bars, 10 μm

The model of IER3IP1 transmembrane domains and the localization of the pathogenic mutations was designed using AlphaFold [40] and created with BioRender.com (Fig.1a).

Statistical analysis was done with GraphPad Prism version 10.0.0 for Windows or 9.0.2 for Mac (GraphPad Software, Boston, MA, www.graphpad.com). The specific statistical test and number of biological replicates are always indicated in the respective figure legend. Only significant p-values are shown.

## Results

### IER3IP1 is cycling between ER and early Golgi and is not essential for cell survival

To investigate the specific role of IER3IP1 in the early secretory pathway, we used CRISPR/Cas9 to knock out (KO) this protein in HeLa cells (Suppl. Fig.S1a-e). Two monoclonal cell lines (KO1 and KO2) were selected. The *IER3IP1* gene was fully deleted in the KO1 clone (Suppl. Fig.S1d, e), and indels were present in the KO2 cells (Suppl. Fig.S1b, c). IER3IP1 protein loss was confirmed by immunoblotting (Fig.1b), using a specific antibody against IER3IP1. Cell growth of both KO1 and KO2 cells, determined by Incucyte live-cell imaging for 72 h, was not significantly different compared to controls (Suppl. Fig.S1f), suggesting IER3IP1 was not essential for HeLa cell survival. Since patient-derived cells were not accessible, we re-expressed wild-type (WT), p.L78P, p.V21G or p.T79∆ mutants (schematized in Fig.1a) in KO1 cells, and clonal cell lines for each variant were selected for further analysis. Re-expressed IER3IP1 WT, p.L78P and p.V21G proteins were detectable by immunoblot (Fig.1b) and by immunofluorescence analysis (Fig.1c, d). In contrast, p.T79∆ protein was not detectable at steady state (Fig.1b), or after incubation with a proteasome (MG132) or a lysosome (chloroquine) inhibitor (Suppl. Fig.S1g), although p.T79∆ *IER3IP1* mRNA was present in these cells (Suppl. Fig.S1h). Together, these data suggest that expression of the *IER3IP1* p.T79∆ variant is equivalent to a KO of* IER3IP1*.

Endogenous IER3IP1 was enriched in the perinuclear region, where it partially overlapped with ER exit-sites (ERES) labeled by the COPII components Sec31A (Fig.1c) or mCherry-Sec24C (Suppl Fig.S1j, k). Partial colocalization was also detected with ERGIC53, a marker of the ER-Golgi intermediate compartment (Fig.1d), EGFP-ERGIC53 (Suppl Fig. S1j, k) and ER marked by mRFP-KDEL (Suppl Fig. S1i). An EGFP-tagged GTP-locked Sar1a (H79G) mutant, which arrests Sec31A on ERES and blocks ER-export [41], colocalized with Sec31A and with IER3IP1 (Fig.1f), indicating that IER3IP1 transits through COPII-coated membranes. Exogenous IER3IP1 WT, p.L78P and p.V21G were also detectable in the perinuclear region, and showed an ER-like reticular pattern, similar to the endogenous protein (Fig.1c, d). In conclusion, IER3IP1 is a component of the early secretory pathway that cycles between ER, ERGIC and the Golgi apparatus. IER3IP1 KO does not affect the expression of COPII coat subunits (Fig.1b) or COPII recruitment at the ER exit sites (Fig.1c). IER3IP1 is dispensable for HeLa cell growth or survival, and MEDS1-causing mutations do not cause obvious changes in its localization.

## IER3IP1 deletion affects ER-export of ERGIC53 and Golgi enzymes

**Fig. 2:**
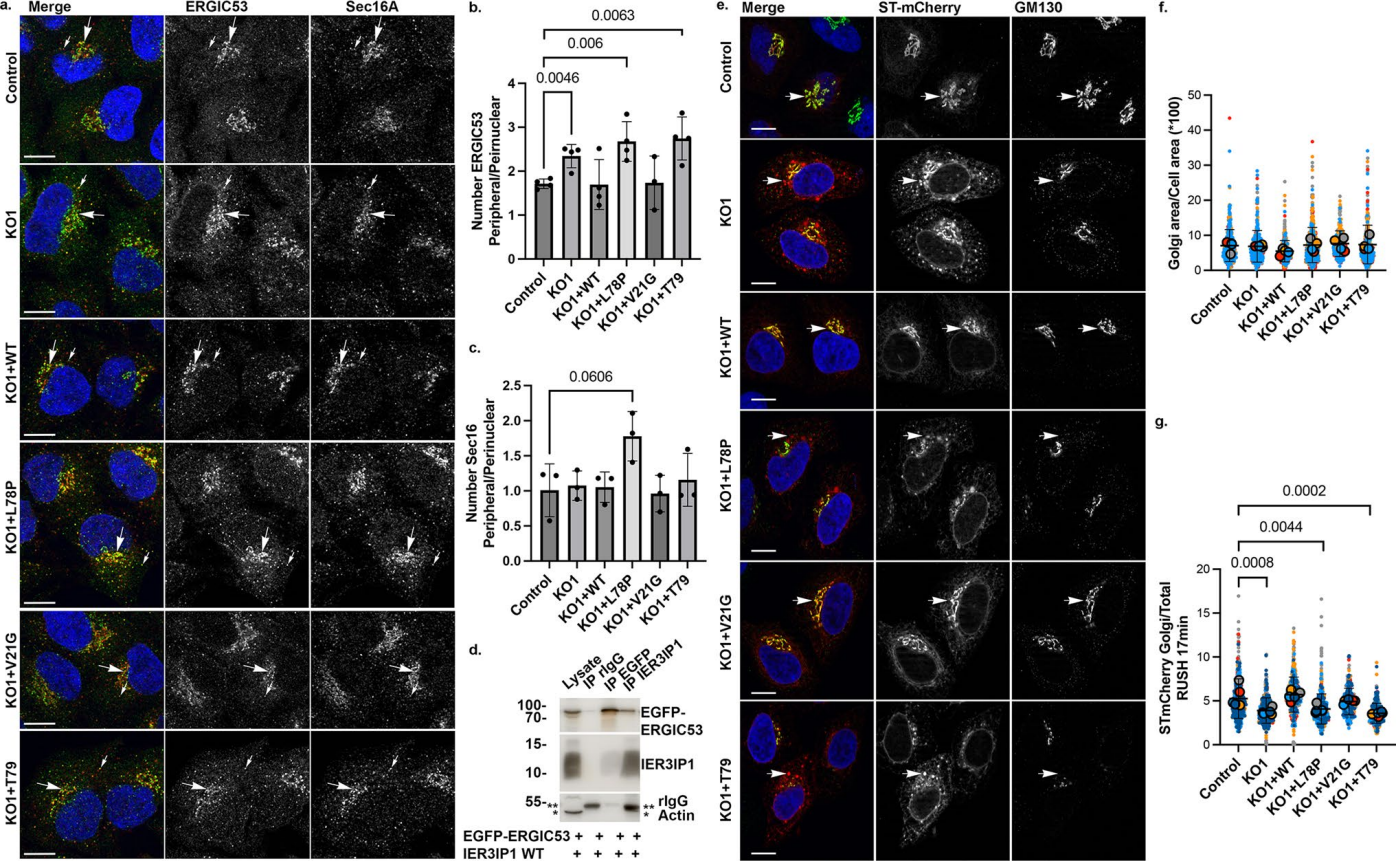
Cargo exit from the ER is delayed in*IER3IP1*KO cells and in cells expressing pathogenic mutations. a) Cells fixed and co-labeled with anti-ERGIC53 (green), anti-Sec16A (red) and Hoechst (blue) were analyzed by fluorescence microscopy. ERGIC53 (b) or Sec16A (c) objects were counted in the cell periphery (small arrows) or in the perinuclear region (large arrows) using Cell Profiler. The areas of the perinuclear regions are delineated in Suppl. Fig. S6a. Ratios of the mean number of peripheral and perinuclear objects are shown for each condition (mean ± SD, unpaired two-tailed t-test, > 222 cells/condition). b) n = 4 except for n _KO1+p.V21G_ = 3. c) n = 3. d) HeLa cells were transfected with the indicated constructs. IER3IP1 and EGFP-ERGIC53 were immunoprecipitated from the lysates using anti-IER3IP1 or anti-EGFP antibodies, and then analyzed by immunoblotting (n = 5 independent experiments). e–g) Indicated cell lines were transfected with Str-STIM-ST-SBP-mCherry (ST-mCherry). After 24 h, cells were incubated with biotin for 17 min, fixed and co-stained with anti-GM130 (Golgi marker, green) and Hoechst dye (blue), and analyzed by fluorescence microscopy. f) The areas of GM130-stained Golgi measured with Fiji in individual cells (small circles), and the color-coded mean values per experiment (large, filled circles) are shown (n = 4 independent experiments, mean ± SD, One-way Anova with Dunnett’s post-hoc test; n _Control_ = 476, n _KO1_ = 435, n _KO1+WT_ = 418, n _KO1+p.L78P_ = 550, n _KO1+p.V21G_ = 252, n _KO1+p.T79∆_ = 312 cells). g) Ratios between Golgi and total (entire cell) ST-mCherry mean fluorescence intensities, measured using Fiji, are shown for individual cells (small circles), and for independent experiments (mean values indicated as large, filled circles) (n = 5 independent experiments, mean ± SD, One-way Anova with Dunnett’s post-hoc test; n _Control_ = 515, n _KO1_ = 475, n _KO1+WT_ = 476, n _KO1+p.L78P_ = 624, n _KO1+p.V21G_ = 293, n _KO1+p.T79∆_ = 368 cells)

We further asked if *IER3IP1* deletion affected the organization of the early secretory pathway. The distribution pattern and the number of Sec31A-labelled ERES [42] in *IER3IP1* KO1 or mutant-expressing cells were similar to those in control or *IER3IP1* WT expressing cells (Fig.1c, e). In contrast, the ratio between peripheral and perinuclear ERGIC53, but not Sec16A (another ERES marker) [42]), puncta was higher in *IER3IP1* KO1 cells, a change rescued by re-expression of the WT and p.V21G, but not of the p.L78P and p.T79∆ variants (Fig.2a-c, Suppl. Figure 6a). Moreover, IER3IP1 co-immunoprecipitated with EGFP-ERGIC53 from cell lysates (Fig. 2d), suggesting that IER3IP1 binds to ERGIC53 and participates in its exit from the ER.

The steady state morphology of cis-Golgi, labeled with an antibody against GM130, was not affected in IER3IP KO or mutant-expressing cells (Fig. 2e, f). To test if IER3IP1 played a role in de novo formation of Golgi stacks, cells were incubated for 1 h with brefeldin A (BFA), a compound that leads to fusion of Golgi membranes with the ER [43] (Suppl. Fig.S2a-d). BFA washout allowed Golgi reassembly, as observed after 2 h. Compared to controls, the formation of novel Golgi stacks was significantly reduced in*IER3IP1*KO1 cells, and it was rescued by re-expression of the WT or p.V21G, but not of the p.L78P or p.T79∆ IER3IP1 variants. Moreover, after Golgi reassembly, the constitutive Golgi transmembrane protein ST6GAL1 co-localized less with GM130 in the absence of IER3IP1, suggesting ST6GAL1 was transported more slowly from the ER to Golgi than in control cells (Suppl. Fig.S2e). This delay was rescued by WT and p.V21G, but not by p.L78P or p.T79∆ IER3IP1. To confirm that IER3IP1 was important for ST6GAL1 transport, we used the retention using selective hook (RUSH) assay [19]. Cells were transfected with a plasmid (ST-mCherry) encoding for a Golgi-localized fragment of ST6GAL1 fused to streptavidin binding peptide and mCherry, and a hook composed of streptavidin fused to an ER retention signal (Str-STIM1) [19]. Upon biotin addition, the interaction between SBP and streptavidin was released, allowing the transport of ST-mCherry from the ER to the Golgi. 17 min after biotin addition, the ratio between Golgi-localized and total cellular ST-mCherry was reduced in IER3IP1 KO1 and KO2 compared to control cells, suggesting a delay in the synchronized transport of ST-mCherry from the ER to the Golgi (Fig.2e, g). This reduced transport was rescued by re-expression of WT and p.V21G, but not by p.L78P and p.T79∆. In sum, IER3IP1 is involved in ER-Golgi transport of ERGIC53 and the Golgi membrane protein ST6GAL1, and the p.L78P and p.T79∆ pathogenic variants impair this function.

## IER3IP1 controls the secretion of specific cargos.

**Fig. 3:**
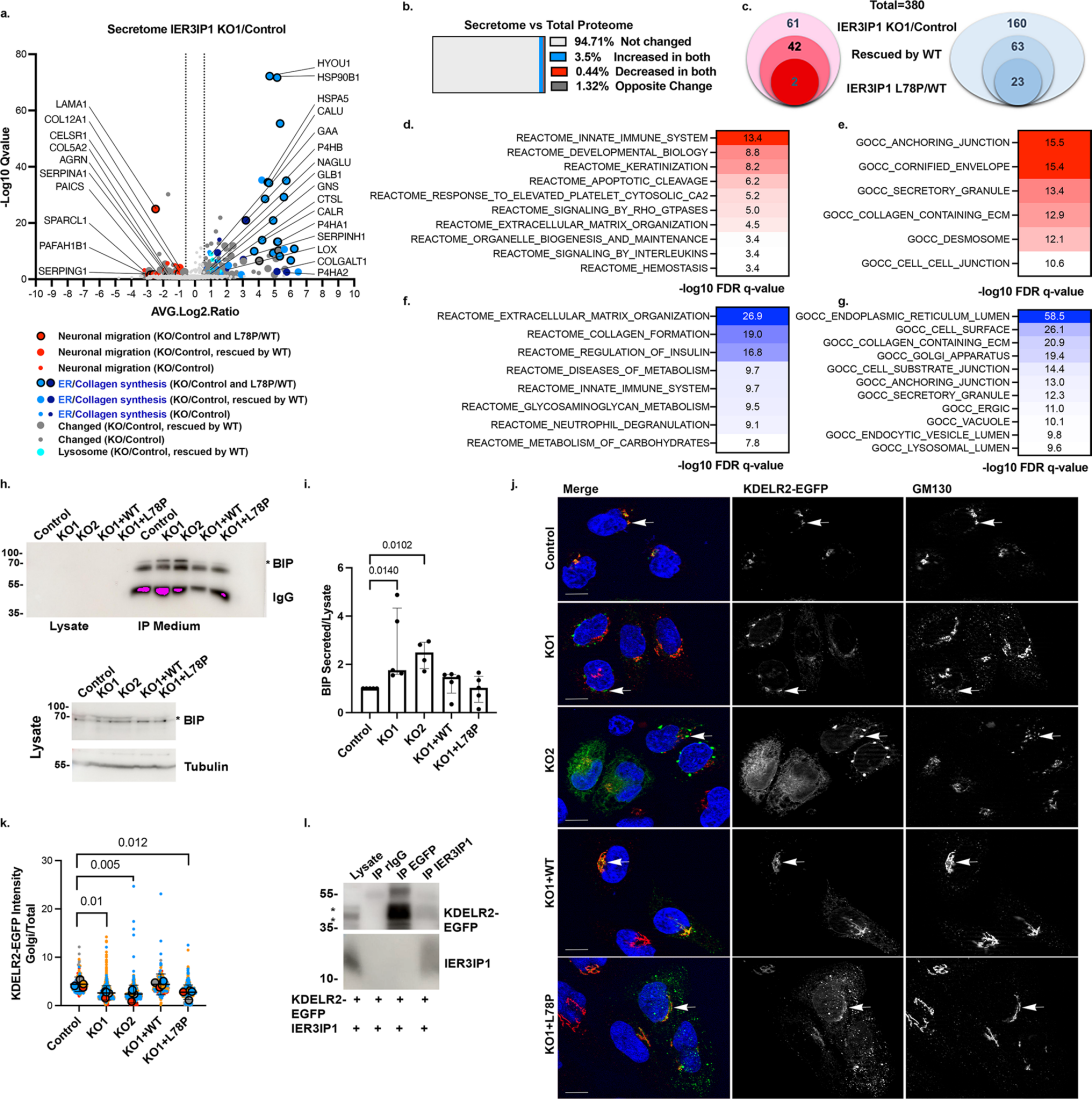
IER3IP1 KO changes the composition of the HeLa cell secretome. a) Volcano plot of proteins differentially secreted by *IER3IP1* KO1 and control HeLa cells (Suppl. Table S2). Proteins are color-coded based on their biological roles (legend). Large circles indicate proteins rescued by *IER3IP1* WT, and filled large circles proteins whose secretion is significantly different in cells expressing the *IER3IP1* p.L78P mutant *versus IER3IP1* WT (n = 3 for KO1 and WT, and n = 4 for control and p.L78P). b) Percentage of secretome hits whose expression levels did not change (grey), increased (blue) or decreased (red) in the total proteome, or exhibited non-concordant changes between their secretion and total levels (dark grey) (Suppl. Tables S2, S3). c) Number of proteins whose secretion is decreased (red) or increased (blue) in *IER3IP1* KO1 cells compared to control (outer circle), those rescued by re-expression of the WT (middle circle), and those differentially secreted in cells expressing *IER3IP1* p.L78P compared to WT expressing cells (inner circle) (Suppl Table S2). d-g) GSEA pathway enrichment analysis of proteins whose secretion was decreased (red) or increased (blue) in *IER3IP1* KO1 compared to control cells. h) BiP was immunoprecipitated from cell medium and analyzed by immunoblotting with specific antibodies. A higher amount of protein lysate was re-run on a separate gel and analyzed by immunoblotting (lower panel). * indicates the band corresponding to the predicted BIP molecular weight (78 kDa). i) Ratios between secreted and total cellular BiP levels; n = 4 independent experiments; median ± intermediate quartile range (IQR); Kruskal–Wallis with Dunn’s post-hoc test. j) Cells were transfected with KDELR2-EGFP (arrows), fixed, co-labeled with anti-GM130 (red) and Hoechst (blue), and analyzed by fluorescence microscopy. Scale bars, 10 µm. k) Ratios of KDELR2-EGFP fluorescence in the Golgi region and the entire cell were calculated using Fiji. Mean values of independent experiments (large, filled circles), and values for individual cells (small circles) are color-coded for each experiment (n = 4 independent experiments, mean ± SD; One-way Anova with Dunnett’s post-hoc test; n ≥ 195 cells/condition). l) HeLa cells were transfected with the indicated constructs, IER3IP1 and KDELR2-EGFP were immunoprecipitated from lysates using anti-IER3IP1 and anti-EGFP, respectively, and analyzed by immunoblotting with anti-EGFP and anti-IER3IP1 (n = 5 independent experiments)

To find proteins whose secretion may depend on IER3IP1, we used click-chemistry to isolate biotinylated, metabolically labeled, newly synthesized glycoproteins from concentrated cell culture medium [33] of control HeLa, *IER3IP1* KO1 cells, as well as cells re-expressing WT or p.L78P *IER3IP1* (Fig.3a, Suppl. Fig.S3a, Suppl. TableS1,S2). A total of 816 proteins were identified by MS analysis, with a high reproducibility (Suppl. Fig.S4j). Among these, 387 proteins were significantly enriched in the biotinylated over the non-biotinylated controls (Suppl. TableS1). Of these, 78.3% were glycoproteins, 80% could be GO term-annotated as either plasma membrane, cell surface, extracellular space, extracellular matrix or extracellular exosome, 36.17% as secreted, 39.27% as transmembrane proteins, and 69.25% contained an ER-signal peptide (Suppl. TableS1).

Overall, 221 proteins were differentially secreted by *IER3IP1* KO1 cells when compared to controls (Fig.3a, Suppl. Table S2), most of which (95%) were not changed in the total proteome (Fig. 3b, Suppl. Tables S2, S3). Interestingly, *IER3IP1* KO reduced the secretion of SERPINA1 (alpha-1-antitrypsin), an established ERGIC53 cargo [44], strengthening the hypothesis that IER3IP1 and ERGIC53 cooperate during membrane transport (see Fig.2). Gene set enrichment analysis (GSEA) showed that *IER3IP1* deletion reduced the secretion of proteins belonging to pathways such as innate immune system, developmental biology, or intercellular junctions (Fig.3d-e, Suppl. Table S2). Significantly, these included factors linked to neuronal migration, such as SERPING1, PAICS, CELSR1, SPARCL1, APP, LAMA1, and collagens. Proteins whose secretion was increased in the absence of IER3IP1 belonged to pathways like ECM organization (Fig. 3f, g). Interestingly, several lysosomal enzymes were abnormally secreted by KO1 cells (Fig. 3a, g; Suppl. Table S2). To check the integrity of the lysosomal compartment in these cells, we used high content confocal fluorescence microscopy (Suppl. Fig. S5). Although the total numbers of LAMP1-positive late endosome/lysosomes were similar (Suppl. Fig. S5b, d), *IER3IP1* KO1 and p.L78P cells had significantly more active lysosomes labeled by Magic Red Substrate (MR-FR_2_) than controls and *IER3IP1* KO1 re-expressing WT *IER3IP1* (Suppl. Fig.S5a, c). Assessed by immunoblotting, the amounts of the autophagosomal proteins LC3B and p62/SQSTM1 [32] were similar to controls in all tested experimental conditions (steady state, Torin1-dependent autophagy induction, chloroquine (CQ)-inhibition of lysosomal function (Suppl. Fig.S5e-g), suggesting autophagy was not affected in the absence of IER3IP1 in HeLa cells.

Interestingly, ~ 27% of all the proteins whose secretion was increased in *IER3IP1* KO1 cells were ER resident proteins (Fig.3g, Suppl. Table S2). These included ER chaperones such as BiP (termed HSPA5 in the MS data), calreticulin and calnexin, that secure the correct folding and quality control of newly synthesized glycoproteins [46], and enzymes required for collagen biosynthesis. Re-expression of *IER3IP1* WT rescued the aberrant secretion of most enzymes (Fig.3a, Suppl. Table S2), and many of them were secreted more by cells expressing the pathogenic mutant p.L78P than by *IER3IP1* WT cells (Fig.3a, Suppl. Fig.S3a; Suppl. Table S2). Immunoblot analysis of BiP immunoprecipitated from cell culture medium confirmed that cells lacking *IER3IP1* secreted significantly more BiP than control cells, and *IER3IP1* WT re-expression rescued these modifications (Fig. 3h, i). Total cellular levels of BiP were not changed (Suppl. Fig. S7c-f), suggesting abnormal secretion was not caused by an increase of the cellular stress response.

More than half of the ER proteins (55.8%) secreted more in the absence of *IER3IP1* carried ER retention motifs (Suppl. Table S4), suggesting a defect in preventing their KDEL receptor-dependent retrieval to the ER [47]. To test this hypothesis, we analyzed the localization of KDELR1-EGFP, KDELR2-EGFP and KDELR3-EGFP by fluorescence microscopy (Fig. 3j, k; Suppl. Fig.S3f-i). All three receptors are endogenously expressed in HeLa cells, with KDELR2 displaying the highest mRNA levels [48]. In control cells, all three receptors co-localized to a high degree with the ci-Golgi marker GM130, as expected [48, 49]. In contrast, the fraction of Golgi-localized KDELR2-EGFP was significantly reduced by *IER3IP1* KO, a change rescued by *IER3IP1* WT, but not by *IER3IP1* p.L78P (Fig. 3j, k). The proportion of Golgi localized KDLER1-EGFP was not changed, and that of KDELR3-EGFP was reduced only in *IER3IP1* KO1 cells, compared to controls (Suppl Fig. S3f-i). In addition, IER3IP1 immunoprecipitated KDELR2-EGFP from cell lysates, albeit not in reverse, suggesting a potential interaction (Fig.3l). Together, these data suggest IER3IP1 may play a role in the transport of KDELR2 from the ER to the cis-Golgi, ultimately ensuring the retention of various ER-resident proteins.

## IER3IP1 controls ER export of specific plasma membrane proteins.

**Fig. 4:**
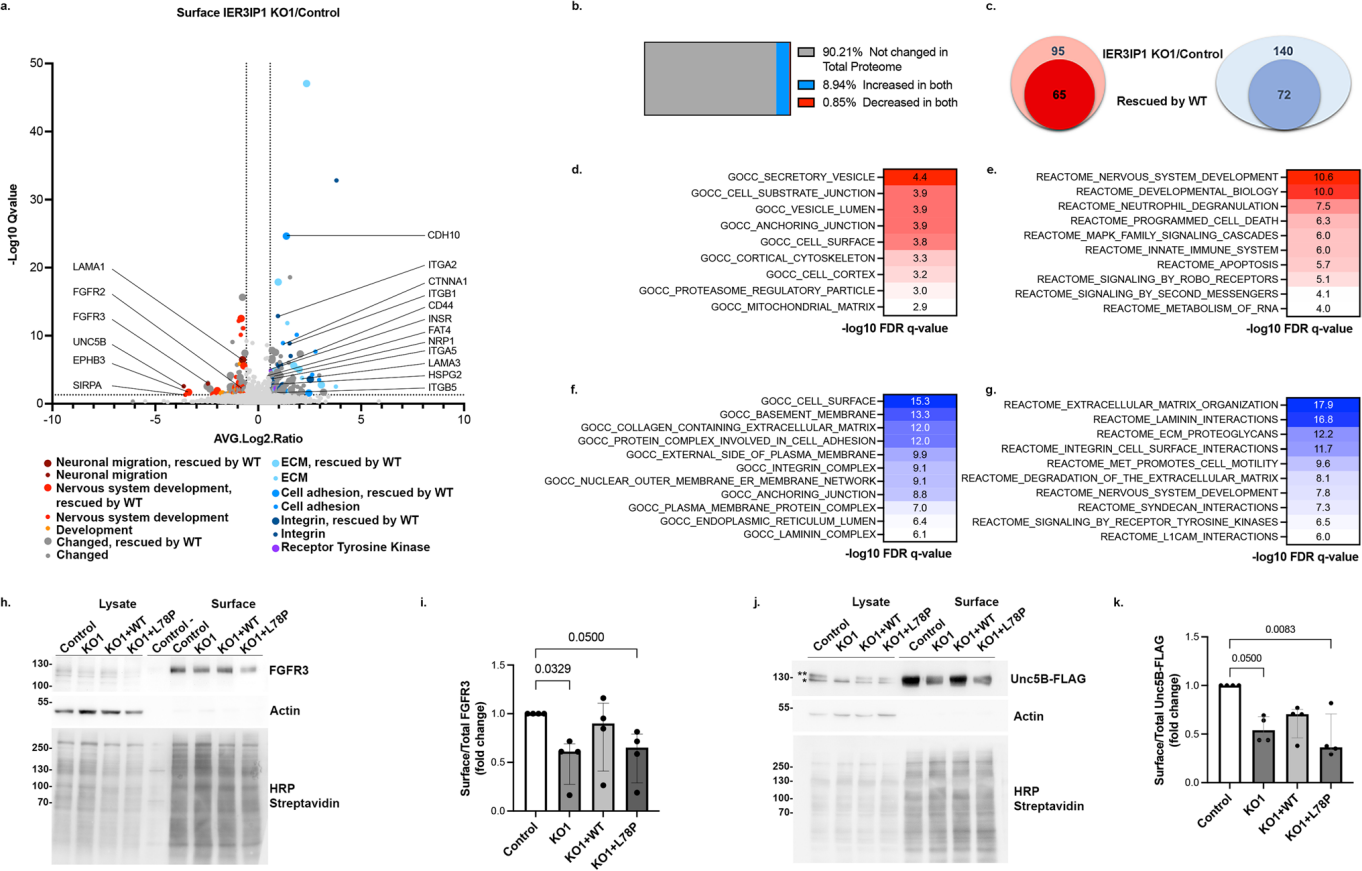
*IER3IP1* KO changes the composition of the surface proteome. a) Volcano plot of proteins differentially regulated on the surface of *IER3IP1* KO1 and control HeLa cells (Suppl. Table S6). Association with various biological pathways was color-coded (legend). Large circles indicate proteins rescued by *IER3IP1* WT (n = 3 independent experiments). b) Comparison of proteins modified on cell surface and in the total proteome (Suppl. Tables S6, S3). c) Number of proteins decreased (red) or increased (blue) on the surface of *IER3IP1* KO1 cells compared to controls (outer circle) and rescued by *IER3IP1* WT (inner circle). d-g) GSEA pathway enrichment analysis of hits either reduced (red) or increased (blue) in KO1 *vs* control (Suppl. Table S6). h–k) Immunoblot analyses of endogenous FGFR3 (h, i) and Unc5B-FLAG (j, k). Cell lines as indicated were subjected to surface biotinylation followed by lysis and streptavidin pull down. After SDS-PAGE and blotting, proteins were detected with anti-FGFR3, anti-FLAG and actin (control) antibodies. Biotinylated proteins were detected with HRP-conjugated streptavidin. **mature, *immature. N = 4 independent experiments; median ± IQR, Kruskal–Wallis with Dunn’s post-hoc test

Does IER3IP1 also control the transport of membrane bound cargos? To get more insights into the role of IER3IP1 in the transport of endogenous cargo, we isolated the surface proteomes of control, *IER3IP1* KO1, and *IER3IP1* KO1 re-expressing WT *IER3IP1* HeLa cells, utilizing cell surface biotinylation and mass spectrometry (MS) (Fig.4a, Suppl. Table S5, S6). In biotinylated control cells, 1006 proteins were significantly enriched over non-biotinylated controls, 31% of which could be GO term-annotated as components of either the plasma membrane, cell surface, extracellular space, extracellular matrix or extracellular exosome, 31% as transmembrane proteins, and 35% as glycoproteins (Suppl. Table S5). A total of 235 surface proteins were differentially expressed in *IER3IP1* KO1 cells compared to controls (Fig.4a, Suppl. Table S6). Among these, 90% were not changed in the total proteome of *IER3IP1* KO1 ve*rsus* control cells, suggesting only their transport was affected (Fig.4b, Suppl. Tables S6, S3). Re-expression of *IER3IP1* WT rescued the surface levels of most proteins (Fig.4c). Pathway enrichment analysis revealed that the reduced proteins on the surface of *IER3IP1* KO1 cells compared to controls (Fig. 4d, e, Suppl. Table S6) were linked to the immune system and to nervous system development. Various proteins implicated in neuronal function were either increased, i.e. integrins (ITGA3, ITGA5, ITGB1) and the semaphorin receptor neuropilin 1, or decreased, i.e. FGFR3 and FGFR2, the netrin 1 receptor UNC5B, the hepatocyte growth factor receptor MET, EPHB3 and laminin subunit alpha 1 (LAMA1) (Fig.4a, d-g). Among the proteins that could be associated with either apical or basolateral plasma membrane domains (http://polarprotdb.ttk.hu/), we observed an enrichment (76.4%) in basolateral proteins on the surface of *IER3IP1* KO1 cells compared to control (Suppl. Table S7). Total proteome analysis showed that cells lacking IER3IP1 upregulated membrane trafficking components, including the COPI subunits COPG1 and COPB2, the p24 protein TMED7 and small GTPases such as RAB6A and RAB11B (Suppl. Fig. S4e-i, Suppl. Table S3), maybe to compensate the absence of IER3IP1. At the same time, *IER3IP1* KO cells downregulated components of the endo-lysosomal compartment. Endocytosis however, assessed by transferrin uptake, was not affected (Suppl. Fig.S6b, c).

Subsequent validation by immunoblotting confirmed that surface levels of endogenous FGFR3 and exogenous Unc5B-FLAG cells were reduced in *IER3IP1* KO1 compared to controls (Fig. 4h-k, Suppl. Fig.S4a, b). Interestingly, these changes were partially rescued by re-expressing *IER3IP1* WT, but not the pathogenic mutant p.L78P. Moreover, mature, complex glycosylated Unc5B-FLAG, indicated by the slower migrating band and representing the surface-localized protein, was significantly reduced in KO1 cells compared to controls (Suppl. Fig.S4c, d). Consequently, the proportion of the lower, immature band (representing the ER-localized Unc5B-FLAG), relative to the total protein amount, was higher. Changes in Unc5B-FLAG glycosylation were rescued by IER3IP1 WT.

**Fig. 5:**
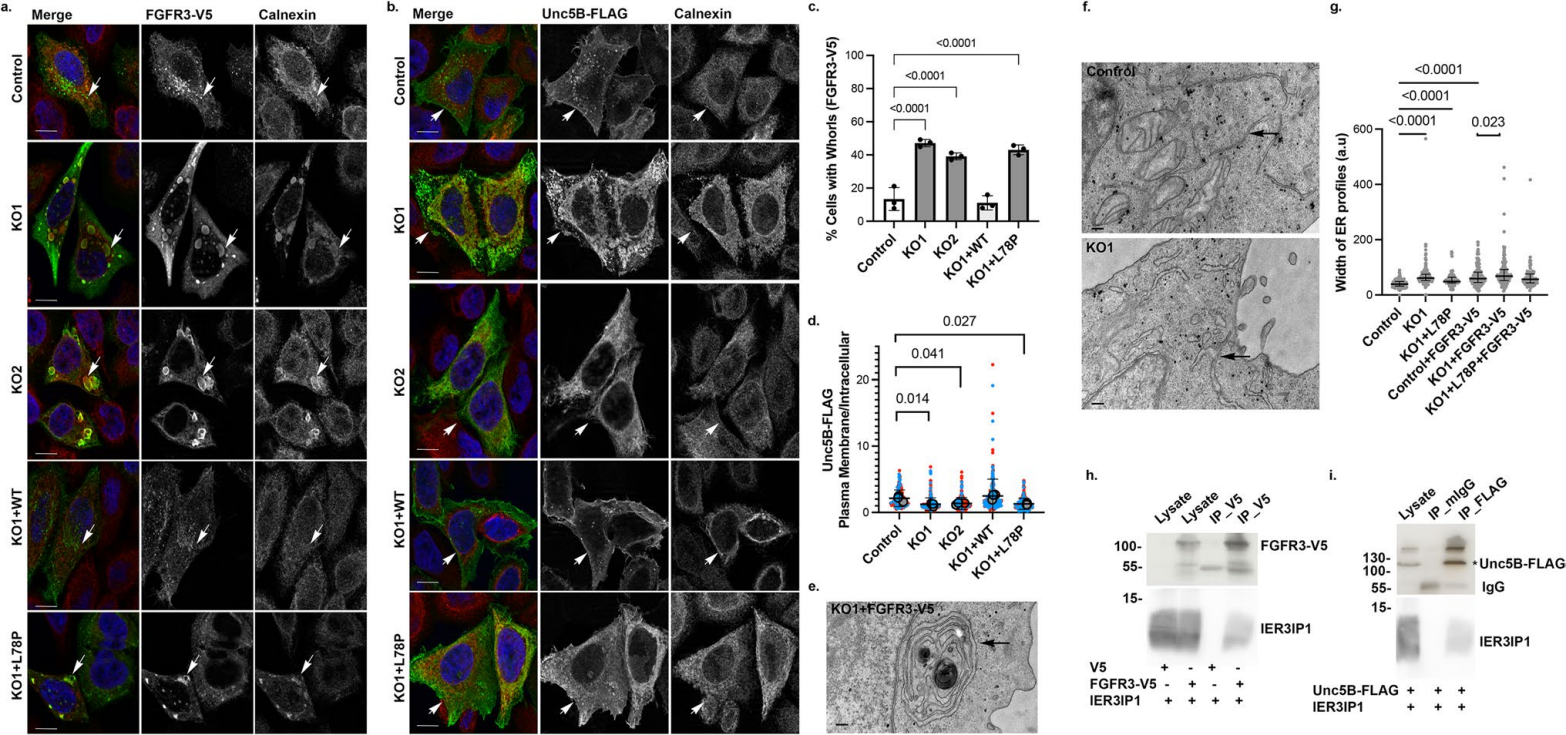
FGFR3 and Unc5B are cargos of IER3IP1. Cell lines as indicated were transfected with FGFR3-V5 (a, c, e, g, h) or Unc5B-FLAG (b, d, i), fixed and labeled with anti-V5 (a, green) or anti-FLAG (b, green), anti-calnexin (red) and Hoechst (blue). Scale bars, 10 µm. c) Percentage of cells expressing FGFR3-V5 and displaying OSER whorls (n = 3 independent experiments; mean ± SD, n ≥ 351 cells/condition). d) Surface/intracellular Unc5B-FLAG fluorescence intensity ratios are shown per cell (circles), and per experiment (filled circles) (n = 3, mean ± SD; n ≥ 173 cells/condition). Statistical significance was analyzed using One-way Anova with Dunnett’s post-hoc test. e–g) Control, *IER3IP1* KO1 and control and *IER3IP1* KO1 transiently transfected with FGFR3-V5 cells were fixed and processed for transmission electron microscopy. Arrows indicate an OSER whorl (e) or ER profiles (f). Scale bars, 200 nm. g) Width of ER profiles was analyzed with Fiji (n ≥ 10 cells/condition; ≥ 74 ER profiles/condition; median ± IQR; Kruskal–Wallis with Dunn’s post-hoc test). h, i) Transiently expressed FGFR3-V5 (h) or Unc5B-FLAG (i) were immunoprecipitated from lysates using anti-V5 and anti-FLAG antibodies, respectively, and analyzed by immunoblotting (n = 6 independent experiments). * indicates a band detected at the predicted Unc5B size

These data concur with immunofluorescence microscopy analysis showing that the receptor was retained in the ER (identified with an antibody against the ER marker calnexin) in *IER3IP1* KO1 and KO2 cells, whereas it was mostly detected on the cell surface in control and *IER3IP1* WT cells (Fig.5b, d). *IERIP1* p.L78P did not rescue the transport of Unc5B-FLAG to the plasma membrane (Fig.5b, d), although it did restore its glycosylation (Fig.4j, Suppl. Fig.S4c, d), suggesting a significant amount of receptor was transported through the Golgi apparatus in these cells. FGFR3-V5 localization was also affected in *IER3IP1* KO1, KO2 and p.L78P cells, where the receptor was detectable in ER membrane “whorls” co-labeled with the ER-marker calnexin (Fig. 5a, c). These whorls resemble OSER (organized smooth ER) observed in various cell types at steady state or upon the overexpression of specific proteins [50, 51]. FGFR3-V5 and Unc5B-FLAG pulled down co-expressed IER3IP1 from cell lysates (Fig.5h, i), suggesting that IER3IP1 interacts with both and controls their ER export.

Using transmission electron microscopy, we confirmed that ER whorls were only detectable in FGFR3-V5 expressing KO1 and p.L78P cells, but not in control cells (Fig.5a, e). Independently of FGFR3-V5 expression, ER profiles appeared dilated in *IER3IP1* KO1 and p.L78P cells *versus* controls (Fig.5f, g). Changes in ER shape were further evaluated by real-time fluorescence microscopy analysis of cells overexpressing the ER membrane protein P180 (Ribosome Binding Protein 1, RRBP1). EGFP-p180-labeled ER membranes covered a significantly higher percentage of the cell area in *IER3IP1* KO1 and p.L78P compared to controls and WT expressing cells (Suppl. Fig. S7a, b). This suggests that, in the absence of IER3IP1, increased protein accumulation in the ER causes membranes to enlarge and, in some cases, to form OSER whorls to accommodate cargo excess.

Whorl formation may represent a novel type of ER stress response that inhibits protein translation by disentangling translocons from ribosomes [52]. Immunoblot analysis of cells overexpressing FGFR3-V5, however, did not show any changes in the levels of the ER stress sensor BiP in the absence of IER3IP1 (Suppl. Fig.S7c, d). Moreover, BiP upregulation was similar in all tested cell lines after incubation with thapsigargin, an ER stress inducer (Suppl. Fig.S7e, f). The levels of phospho-IRE1 (pIRE-1) (Suppl. Fig.S7g) and ATF6 (Suppl. Fig.S7h, i), proteins involved in the UPR response, were not changed either, compared to controls, at steady state or after incubation with thapsigargin. These findings were consistent with the mass spectrometry analysis of the total proteomes, where no significant upregulation of the ER stress response was observed in cells lacking IER3IP1 (Suppl. TableS3). In summary, IER3IP1 selectively controls the surface transport of a subset of membrane proteins, including FGFR3 and UNC5B.

## IER3IP1 is involved in the secretion/surface localization of selected cargo in human i3neurons

**Fig. 6:**
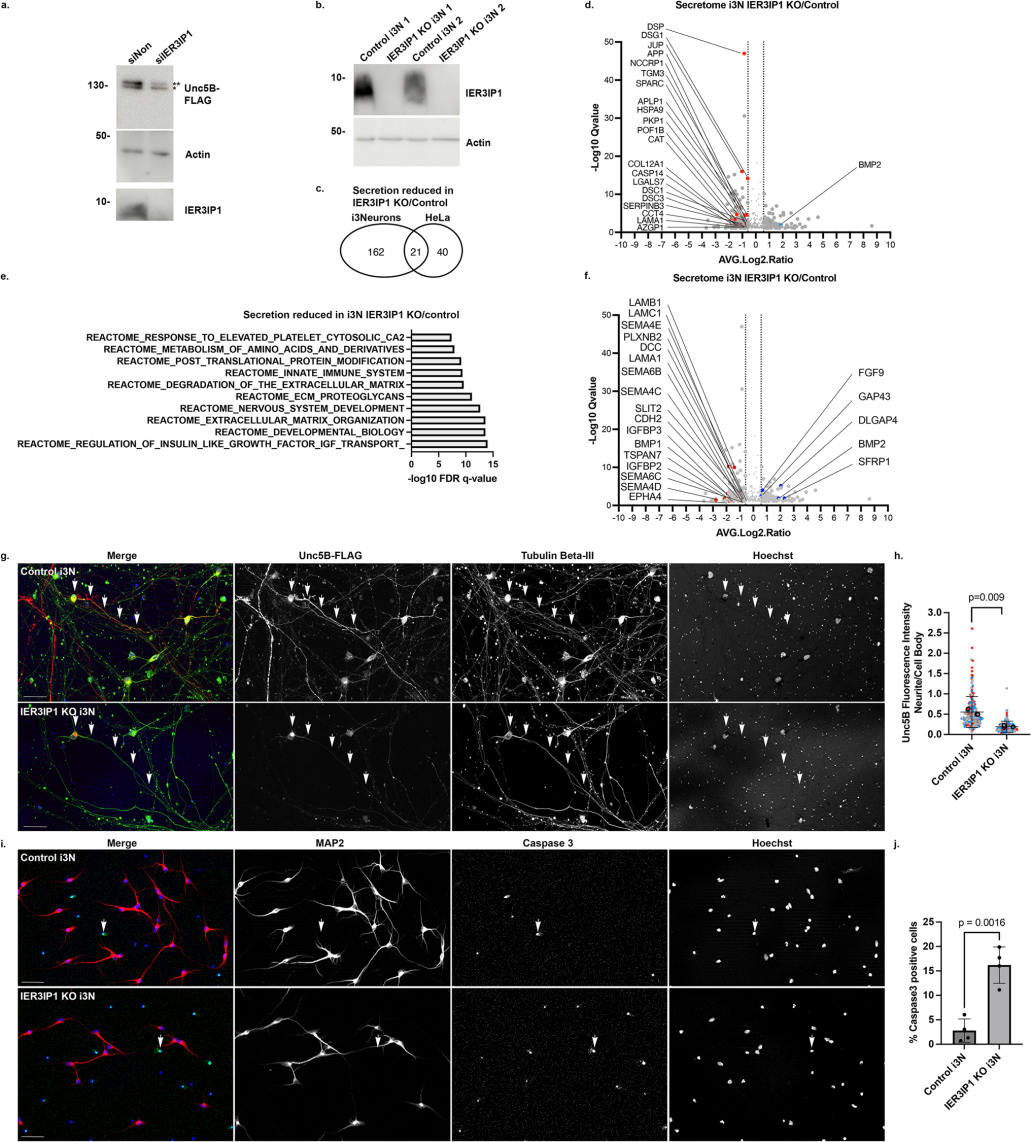
IER3IP1 controls the transport and secretion of cargos essential for neuronal function and survival in neuronal cells. a) IER3IP1 KD affects Unc5B-FLAG transport and maturation in SH-SY5Y neuroblastoma cells. Cells were incubated with control siNon or siIER3IP1 for 48 h, then transfected with Unc5B-FLAG for 24 h. Lysates were analyzed by immunoblot, and proteins were detected with anti-FLAG and anti-β-actin as loading control (n = 3 independent experiments). **mature, *immature. b-j) *IER3IP1* KO affects protein secretion and the survival of i^3^ neurons. Control and *IER3IP1* KO iPSC cells were differentiated into i^3^ neurons. b) KO efficiency was confirmed by Western blot using antibodies against IER3IP1 and β-actin. Cell lysates from two independent differentiations (1 and 2) are shown (n = 4 independent differentiations). c-f) Analysis of i^3^ neuron secretome. Secreted glycoproteins were enriched from i^3^ neuron growth medium by immunoprecipitation with Concanavalin A magnetic beads, then analyzed by mass spectrometry. The full list of identified proteins is shown in Suppl. Table S8 (n = 4 independent i^3^ neuronal differentiations). c) The Venn diagram illustrates the overlap between the proteins whose secretion was reduced by *IER3IP1* KO in i^3^ neurons (Suppl. Table S8), *versus* HeLa cells (Suppl. Table S2), compared to their respective controls. d) Volcano plot of proteins differentially secreted by *IER3IP1* KO1 *vs* control i^3^ neurons. Hits common to i^3^ neurons and HeLa cells are shown in red (decreased secretion) or blue (increased secretion), respectively. e) GSEA pathway enrichment analysis of proteins whose secretion was decreased in *IER3IP1* KO compared to control i^3^ neurons. f) Proteins with a known role in neuronal development are indicated on the Volcano plot displaying all differentially secreted proteins (red = decreased; blue = increased secretion) in *IER3IP1* KO compared to control i^3^ neurons. g) Control and *IER3IP1* KO i^3^ neurons were transfected at day12/13 with Unc5B-FLAG, fixed after 24 h, labeled with anti-FLAG (red), anti-β-tubulin-III (green) and Hoechst (blue) and analyzed by fluorescence microscopy. Arrows indicate the cell body and the longest neurite, labeled by β-tubulin-III. h) Average fluorescence intensities along the longest neurite and in the soma were measured using ImageJ, and the ratios between them were plotted (n = 3 independent differentiations, > 123 cells/condition). i) i^3^ neurons were fixed at day 10, labeled with anti-caspase 3 (green), anti-MAP2 (red) and Hoechst (blue) and analyzed by fluorescence microscopy. Arrows indicate caspase-3 positive cells. j) The percentages of caspase 3-positive cells are shown (n = 4 independent differentiations, > 143 cells/condition). Scale bars, 50 μm

Although we identified many neuronal proteins misregulated in the absence of IER3IP1, our data so far were derived from unpolarized undifferentiated cell lines. To test whether our findings are relevant in a neuronal context, we made use of SH-SY5Y cells (Fig.6a) and i^3^ neurons, human iPSC-derived glutamatergic neurons that were generated by the induction of a stably integrated neuronal transcription factor, NGN2 [34, 53] (Fig.6b-j). *IER3IP1* knock-out iPSCs were generated, validated by Western blot (Fig.6b) and, in parallel with the parental cells, differentiated into neurons for the indicated times.

Analysis of the secretome of i^3^ neurons collected between day 12 and day 14 of differentiation identified a total of 410 proteins, of which 325 were changed (183 decreased and 142 increased) in *IER3IP1* KO compared to control cells (Suppl. Table S8). One third of the proteins whose secretion was reduced in HeLa *IER3IP1* KO1 were also significantly lower in *IER3IP1* KO i^3^ neurons compared to controls (Fig. 6c). These included laminin LAMA1, collagen COL12A1, and the desmosomal adhesion molecules PKP1, DSC3, DSP, DSC1, DSG1, JUP (Fig. 6d). Desmosomes are cellular adhesions whose function in the brain is not well understood, however, neuron-specific KO of Dsp (desmoplakin) affects the proliferation of neuronal progenitors and their differentiation [54]. Other common hits included catalase/CAT which protects neurons from oxidative stress [55], AZGP1, a regulator of energy metabolism [56], amyloid precursor-like protein 1 (APLP1), the neurotrophic amyloid precursor protein (APP) [57] and CCT4 which was linked to sensory neuropathy [58].

Overall, the number of proteins whose secretion was reduced in *IER3IP1* KO i^3^ neurons compared to control was higher than in HeLa cells (Fig.6c). Among the proteins identified in i^3^ neurons, there was a significant enrichment in pathways such as regulation of the insulin like growth factor IGF transport, extracellular matrix organization and nervous system development pathways (Fig.6e). The latter included guidance molecules such as EPHA4, the netrin receptor DCC, SLIT2, PLXNB2, N-cadherin (CDH2) and multiple semaphorins (Fig. 6f). In agreement with these findings, the transport of Sema4D-myc was affected in HeLa cells in the absence of IER3IP1, with more protein detectable in OSER whorl-like structures, compared to control (Suppl. Fig.S8). In *IER3IP1* KO HeLa cells the reduction of SEMA4D secretion was relatively close to significance levels (ratio IER3IP1 KO/Control = 0.168, Q-value = 0.059, p-value = 0.052) compared to controls (Suppl. TableS2).

Unc5B-FLAG transport and maturation were delayed in the absence of IER3IP1 in HeLa cells (Fig.4,5) and in glioblastoma SH-SY5Y cells (Fig. 6a). In i^3^ neurons, Unc5B-FLAG was detectable in both the soma and neurites in control cells, but mostly restricted to cell bodies of *IER3IP1* KO cells, as reflected by the reduced ratio between Unc5B-FLAG in the neurites relatively to the cell body in KO compared to control cells (Fig. 6g, h), suggesting a transport defect also in these cells. Apoptosis was significantly higher in *IER3IP1* KO i^3^ neurons compared to control (Fig. 6i, j). Thus, IER3IP1 controls Unc5B trafficking in neurons, validating the findings in HeLa and SH-SY5Y cells, and is essential for neuron survival.

## Down-regulation of *Ier3ip1* affects neuronal morphology in the embryonic brain.

**Fig. 7:**
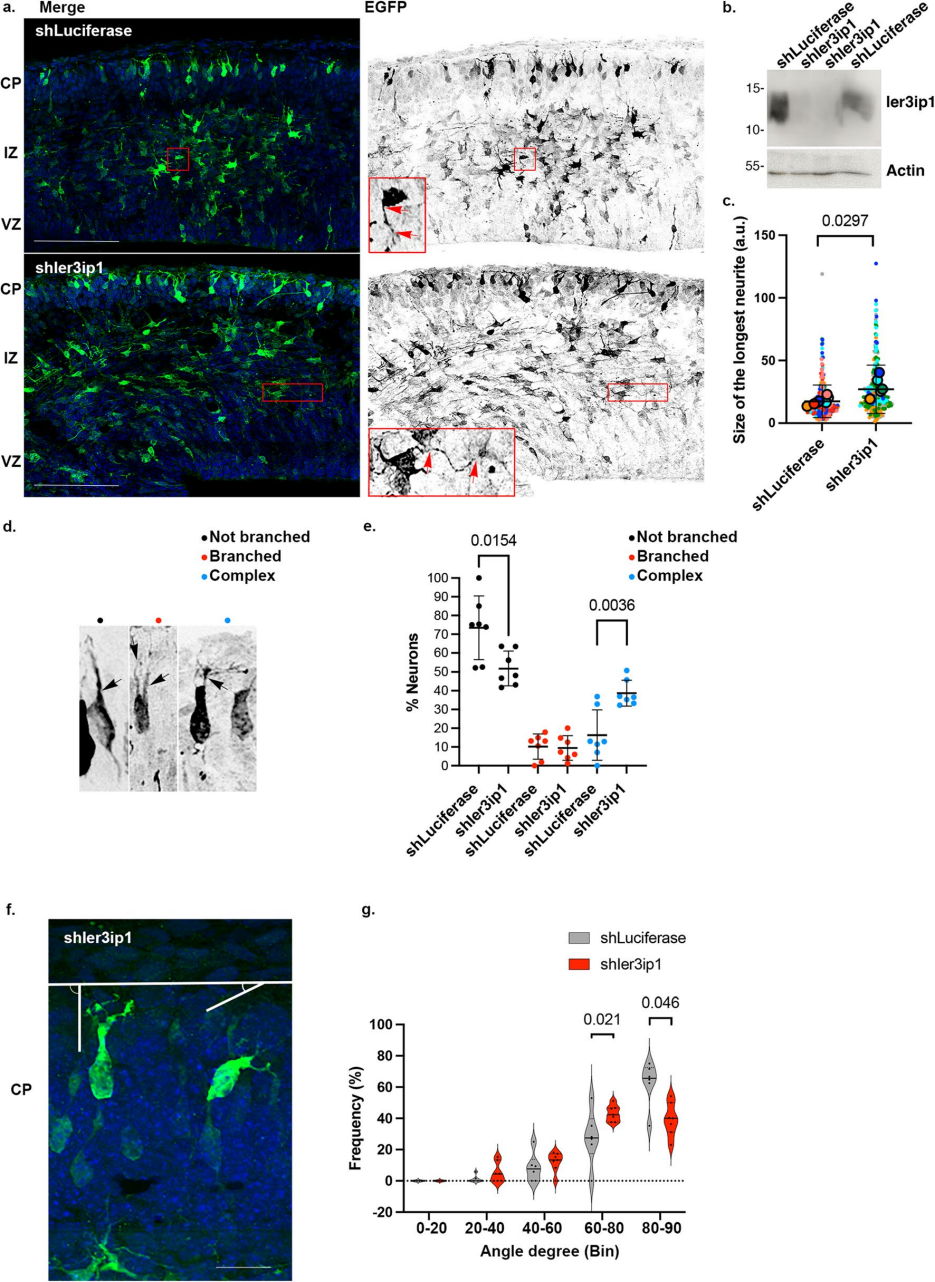
*Ier3ip1* knockdown affects neuronal morphology in vivo. Brains were electroporated with shRNAs at E13.5 and collected at E15.5. a) Coronal sections were fixed and labeled with the indicated antibodies and Hoechst nuclear dye. Right panels show inverted EGFP signal (black). Inserts show selected cells at a higher magnification. Ventricular zone (VZ), intermediate zone (IZ) and cortical plate (CP) were distinguished based on nuclear density and morphology, and co-staining with specific markers (not shown). Scale bars, 100 μm. c) The longest neurite in each analyzed cell (arrows) was measured, and color-coded values for individual cells (small circles). Mean values per brain (circles) are shown (n _shLuciferase_ = 7 brains, 302 cells; n _shIer3ip1_ = 5 brains, 280 cells; mean ± SD; two-tailed Welch’s t-test). b) NIH3T3 cells were transfected with shRNAs for 3 days, and EGFP + cells were sorted and analyzed by immunoblotting (n = 3). d, e) EGFP + neurons in the CP were classified as unbranched uni/bipolar cells (black circles), bipolar branched (red circles) or neurons displaying complex arborizations (blue circles) and quantified (n _shLuciferase_ = 7 brains, 9 sections, 338 EGFP + cells; n _shIer3ip1_ = 7 brains, 10 sections, 475 cells; mean ± SD, two-tailed Welch’s t-test). Scale bars, 100 μm. f) The angles between the leading neurites of EGFP + neurons and the CP surface were determined, and (g) the frequencies (%) of the values within the ranges of values that define the indicated bins are shown as percentages of the total number of values. n _shLuciferase_ = 7 brains, 10 sections, 185 EGFP + cells; n _shIer3ip1_ = 7 brains, 11 sections, 203 EGFP + cells; Violin plot with indicated median value (black line) and quartiles (dashed lines), two-tailed Mann––Whitney test. Scale bar, 20 μm. All images represent maximum projections of sequential Z-sections

Our data suggest that loss of IER3IP1 causes changes in secreted and surface-localized proteins involved in neuronal development and migration. To test if Ier3ip1 depletion affects neurons in vivo, the lateral cortex of E13.5 mouse embryos was used as a model region (Fig. 7). Neural progenitors were targeted by *in-utero* electroporation [59] with plasmids expressing EGFP and shRNAs against either *Ier3ip1* (efficiency shown in Fig. 7b) or luciferase as a control. Their EGFP + neuronal progeny was analyzed two days later at E15.5 (Fig. 7a). At this time point, shIer3ip1 had no major effect on neuronal migration (Suppl. Fig. S9a, b), progenitor cell cycle exit (Suppl. Fig. S9c, d), basal progenitor differentiation (Suppl. Fig. S9e, g), or neuronal survival (Suppl. Fig. S9f, h).

Upon leaving the ventricular zone/subventricular zone (VZ/SVZ), newborn neurons are multipolar and generate several extremely dynamic tangential processes [59, 60]. In the upper part of the intermediate zone (IZ), neurons become bipolar while undergoing migration towards the cortical plate (CP) [60, 61]. We found that in the IZ, neurons with *Ier3ip1* KD displayed significantly longer neurites compared to controls (Fig.7a, c), suggesting changes in the neurite extension/retraction cycle. When CP-localized neurons were classified based on their morphologies [39], the percentage of neurons with complex arborizations was higher in *Ier3ip1* KD cells than in the controls, at the expense of unbranched uni/bipolar cells (Fig.7d, e). The angles between the leading neurites relative to the orientation of the CP showed a higher variability in *Ier3ip1* KD neurons, where significantly more neurons were tilted at angles of 60°–80° compared to control, where most neurons had orientations between 80°–90° (Fig.7f, g). In conclusion, the loss of IER3IP1 affects the dynamic transition between subsequent morphological stages, likely by changing the equilibrium of secreted and surface-localized proteins (i.e. netrin receptors UNC5B and DCC, semaphorins) required for the precise coordination of intracellular and extracellular signals [62].

## Discussion

Here we combined proteomics and functional approaches to characterize changes in membrane trafficking in cells lacking *IER3IP1* or expressing *IER3IP1* mutants that are associated with MEDS1, a fatal syndrome affecting multiple systems and organs, including the brain. Among the *IER3IP1* pathogenic variants, p.T79∆ is not expressed at the protein level, p.L78P negatively affects the organization of the secretory pathway, similarly to *IER3IP1* depletion, whereas p.V21G causes no significant morphological changes, suggesting further investigations are necessary for its characterization. Although previous studies support the importance of IER3IP1 in ER function [10, 14, 15], a direct role for IER3IP1 in ER to Golgi transport has not been demonstrated in mammalian cells, and its potential cargos have remained unknown. Our data indicate that IER3IP1 is involved in the ER export of a subset of secretory and plasma membrane proteins, some of which play a role in axon pathfinding, neuronal development and survival, suggesting a potential pathomechanism for MEDS1. In vivo, the morphology of neurons lacking IER3IP1 is modified, with longer and more branched neurites. ERGIC53 and KDELR2, two important receptors in the early secretory pathway, are affected by the absence or malfunction of IER3IP1. This suggests a possible molecular mechanism for the changes in the surfaceome and secretome, with potential consequences for cellular homeostasis in MEDS1.

### How does IER3IP1 promote the ER exit of specific cargoes?

IER3IP1 controls the export of a subgroup of proteins from the ER, indicating it plays a role in their selective recruitment to ERES. The mechanism could involve IER3IP1 interactions with soluble proteins via a luminal domain, as seen for SURF4 [63] and ERGIC53 [64], or the chaperoning of transmembrane domains, as described for the classical cargo receptor cornichon/Erv14p [65]. This function may be performed as a cofactor of cargo receptors like ERGIC53. IER3IP1 could add complexity to this system by conferring specificity towards a certain subset of cargos, or by maintaining trafficking-favorable protein conformations [66]. In agreement with this hypothesis, IER3IP1 might bind ERGIC53 and might control both ERGIC53 trafficking and the secretion of SERPINA1 (alpha-1-antitrypsin), a known cargo of ERGIC53 [44]. Although ERGIC53 has not been associated with neurological defects but with bleeding disorders, its family member LMAN2L, whose cargoes are not known, is mutated in patients with intellectual disability and epilepsy [67].

The ER in cells lacking IER3IP1 displayed increased susceptibility to OSER formation [50, 51] upon overexpression of membrane proteins like KDELR2, FGFR3 and Sema4D. This may indicate a protective role for IER3IP1 in OSER formation and ER retention. For example, IER3IP1 may prevent interactions between proteins on apposed ER stacks, or between proteins and ER chaperones (i.e. calnexin) [50, 51]. The *IER3IP1* L78P mutant was not able to counteract OSER formation upon overexpression of the proteins mentioned above, suggesting the potential conformational changes resulting from this pathogenic mutation affect this process.

### ER enzymes are not efficiently retrieved to the ER in the absence of IER3IP1.

Our data suggest that the role of IER3IP1 in ER-export has implications for another important feature of the early secretory pathway, the retention of ER resident proteins. Rather strikingly, cells lacking *IER3IP1* or expressing *IER3IP1* p.L78P abnormally secrete ER resident proteins with C-terminal KDEL or KDEL-like sequences like BiP, whose localization to the ER requires KDELRs [68]. Three KDELRs (KDELR1-3) traffic between the ER and Golgi via COPII- and COPI-coated carriers [69], but their specific functions are not well understood. It has been suggested that siRNA-mediated knockdowns of both *KDELR1* and *KDELR2* are necessary to efficiently reduce ER protein retrieval [70]. The interpretation of this study is, however, complicated by the fact that none of the two receptors was fully depleted. In contrast, the knockout of *KDELR1* alone was sufficient for increasing the secretion of an ER resident protein [71]. Only KDELR2 is mislocalized in the absence of IER3IP1, suggesting decreased amounts of KDELR2 on the Golgi are sufficient to cause the abnormal secretion of ER resident enzymes. The importance of KDELR2 is supported by the finding that its mutation causes osteogenesis imperfecta and neurodevelopmental delay [72]. An alternative mechanism that could explain the abnormal chaperone secretion could be similar to the one described for p24 [73]. In this case, reduction of p24 diminishes incorporation of its specific cargoes into ERES, allowing the recruitment of unspecific cargos that are normally not secreted.

### Consequences of secretion of ER chaperones

In the case of most ER resident proteins, in normal conditions only small amounts escape the ER and need to be retrieved [74]. Their reduced retrieval and increased secretion may ultimately compromise ER quality control [75], allowing the export of malfunctioning/misfolded proteins. A mouse model expressing an abnormally secreted BiP mutant has a severe neurological phenotype including microcephaly, defects in neuronal migration, cortical layer organization, and early death [76]. SIL1, a nucleotide exchange factor for BiP, is required for maintaining cortical architecture and insulin secretion [77]. Two other ER enzymes have been directly linked to microcephaly: POFUT1 [78] and MINPP1 [79]. Several ER chaperones secreted by IER3IP1-deficient cells (P4HB, P4HA1, P3H1, FKBP10, SERPINH1, CRTAP, COLGALT1, PLOD1, PLOD2, and LOX) are essential for the synthesis of collagens controlling ECM stiffness and cell migration [80]. Furthermore, some ER chaperones have known functions in the extracellular space. Secreted calreticulin mediates fibrinogen-dependent mitogenic activity [81] or cell spreading [82], and secreted BiP controls neurite growth [83], processes that could be disturbed by their enhanced secretion, as observed here in the absence of IER3IP1.

Does the secretion of chaperones and the accumulation of selected cargo in the ER cause ER stress? Defective protein folding could result in cargo accumulation, causing the enlargement of the ER cisternae, as observed in the absence of IER3IP1. This is in agreement with observations in cells lacking ER exit regulators such as TANGO1 [84], Sec24 [85], Sec23B [86], Sec13 [87] or SAR1 [88]. The distended ER stacks observed in HeLa cells lacking IER3IP1, suggest a certain level of ER stress, but BiP protein levels are not increased. The ER stress sensors phospho-IRE1 and ATF6 are not activated, as previously observed in pancreatic β-cells [14]. IER3IP1 deficiency-related accumulation of enlarged ER cisternae is likely to activate cellular mechanisms that remove unnecessary membranes for maintaining cellular homeostasis [69]. Lysosomal activity and secretion of lysosomal enzymes were higher in the absence of IER3IP1, disturbances that may also contribute to MEDS1 pathology.

### IER3IP1 controls the composition of the cellular secretome and surfaceome, and thereby neuronal survival and morphology

Several cargos exported with the help of IER3IP1 are essential for neuronal function, and their identification may help to understand some of the clinical features described for MEDS1. Interestingly, these included an increase of basolateral proteins, which may shift apical-basal polarity during neurogenesis [89]. Neuronal morphological differentiation and migration are precisely coordinated by gradients of diffusible signaling molecules and plasma membrane receptors, surface bound cues and physical properties of the substrate (i.e., ECM stiffness) [80]. Our data in HeLa cells and i^3^ neurons suggest that not one, but several factors are changed in the absence of IER3IP1, including ECM components like collagens and laminin LAMA1, in agreement with observations in *IER3IP1* KO brain organoids [15]. LAMA1 is a major component of basal membranes, whose mutations affect neuronal development [90]. Interestingly, LAMA1 and transient axonal glycoprotein 1 (TAG-1), an adhesion molecule, are essential for the polarization of multipolar cells in the intermediate zone [91], where we have as well observed changes in neurite extension. In contrast, a subset of integrins are upregulated on the surface of *IER3IP1* KO HeLa cells. Together, these data suggest that laminin/integrin signaling, essential for progenitor proliferation and migration [92], as well as for neuronal migration [93], is disrupted in the absence of IER3IP1.

FGFR3 [94] and FGFR2 [95], essential for neuronal development, are both reduced on the surface of *IER3IP1* KO HeLa cells. Although cortical development is not affected in Fgfr3-null mice models, microcephaly has been observed in zebrafish lacking fgfr3 [96, 97]. Moreover, in humans, a potentially inactivating mutation in the catalytic domain of human FGFR3 causes developmental delay and, in some patients, microcephaly [98]. Two other examples of receptors reduced on the surface of *IER3IP1* KO HeLa cells are TGFBR3 and ACVR2B, components of the TGF beta signaling pathway, which is essential for neuronal differentiation and nervous system development [99]. Future experiments are needed to confirm if IER3IP1 is required for FGFR2, FGFR3 and/or TGF beta receptor trafficking in neurons.

IER3IP1 controls the transport of proteins implicated in axon guidance and neuronal migration (i.e. Unc5B, semaphorin 4D) not only in HeLa cells, but also in i^3^ neurons. Unc5B is a repulsive axonal guiding receptor [100–103], and its lower levels in the peripheral neuronal compartment may modify neurite dynamics, explaining why sh*Ier3ip1*-transfected neurons extend longer processes in vivo. A reduced amount of Unc5B on neuronal surface may render it less exposed/sensitive to its ligand netrin 1, leading to a dysregulation of their common role in preventing apoptosis [104, 105]. Changes in semaphorin/plexin [106–108] trafficking and signaling may also influence cell survival, in addition to other aspects of neuronal development [109]. This may, at least partially, explain the increased apoptosis of the *IER3IP1* KO i^3^ neurons.

How does deficiency of *IER3IP1* cause microcephaly? In vitro, *IER3IP1* KD in i^3^ neurons increases apoptosis. Although in vivo we could not observe increased neuronal cell death 48 h after *Ier3ip1* depletion, further experiments are necessary to characterize neuronal survival at later time points. In vivo, *Ier3ip1* depletion alters neuronal morphology in the developing cortex. Neurite morphology is essential for establishing neuronal connectivity and changes have been linked to neurodevelopmental disorders [110]. For instance, microcephaly has been associated with mutations in *RAB3GAP/RAB18* [111, 112], *ARFGEF2* [113], *ARF1* [114, 115], kinesin *KIF2A* [2], or dynein heavy chain [116], all affecting both membrane trafficking [117] and neuronal morphogenesis.

In summary, we found that IER3IP1 deletion affects the surface levels and/or the secretion of multiple molecules implicated in neuronal development, suggesting that not a single factor, but the different composition of the neuronal membrane and its environment are responsible for the observed phenotype.

### Limitations of the study

Our research underscores the mislocalization of key proteins in neurodevelopment, migration, and axon pathfinding due to IER3IP1 deficiency, influencing neuronal morphology. However, our study has certain limitations. We lack a detailed understanding of the specific impact of individual mislocalized proteins on the observed phenotype. Future studies should unravel the distinct roles of these proteins in the context of IER3IP1 deficiency. Additional research is needed to explore IER3IP1's role in different cell types in the cortex and during various developmental stages. While our study touches upon neuronal morphology, further exploration is needed to understand the effects of IER3IP1 on progenitor proliferation, neurogenesis and migration. The distinction between cell-extrinsic and cell-intrinsic roles of mislocalized secreted factors remains unclear and should be addressed in future investigations. Our study does not delve into certain aspects of MEDS1, such as juvenile diabetes implications and the specific role of malfunctioning IER3IP1.

In conclusion, while our study provides valuable insights into IER3IP1's role in neurodevelopment and microcephaly, addressing these limitations through further research will enhance our understanding of the intricate molecular mechanisms involved.

## Supplementary Information

Below is the link to the electronic supplementary material.

Supplementary file1 (PDF 5504 KB)

Supplementary file2 (XLSX 279 KB)

Supplementary file3 (XLSX 440 KB)

Supplementary file4 (XLSX 1793 KB)

Supplementary file5 (XLSX 31 KB)

Supplementary file6 (XLSX 935 KB)

Supplementary file7 (XLSX 2389 KB)

Supplementary file8 (XLSX 13 KB)

## Data Availability

The proteomic datasets generated during and/or analyzed during the current study are available in the MassIve repository, dataset ID MSV000093547 (HeLa cells) and MassIVE MSV000095101 (i^3^ neurons).

## References

[1] Gilmore EC, Walsh CA (2013) Genetic causes of microcephaly and lessons for neuronal development. Wires Dev Biol 2:461–478. 10.1002/wdev.89 10.1002/wdev.89 PMC3767923 24014418

[2] Ruiz-Reig N, Chehade G, Hakanen J, Aittaleb M, Wierda K, De Wit J, Nguyen L, Gailly P, Tissir F (2022) KIF2A deficiency causes early-onset neurodegeneration. Proc Natl Acad Sci U S A 119:e2209714119. 10.1073/pnas.2209714119 36343267 10.1073/pnas.2209714119 PMC9674219

[3] Asselin L, Rivera Alvarez J, Heide S, Bonnet CS, Tilly P, Vitet H, Weber C, Bacino CA, Baranano K, Chassevent A, Dameron A, Faivre L, Hanchard NA, Mahida S, McWalter K, Mignot C, Nava C, Rastetter A, Streff H, Thauvin-Robinet C, Weiss MM, Zapata G, Zwijnenburg PJG, Saudou F, Depienne C, Golzio C, Heron D, Godin JD (2020) Mutations in the KIF21B kinesin gene cause neurodevelopmental disorders through imbalanced canonical motor activity. Nat Commun 11:2441. 10.1038/s41467-020-16294-6 32415109 10.1038/s41467-020-16294-6 PMC7229210

[4] Poulton CJ, Schot R, Kia SK, Jones M, Verheijen FW, Venselaar H, de Wit MCY, de Graaff E, Bertoli-Avella AM, Mancini GMS (2011) Microcephaly with simplified gyration, epilepsy, and infantile diabetes linked to inappropriate apoptosis of neural progenitors. Am J Hum Genet 89:265–276. 10.1016/j.ajhg.2011.07.006 21835305 10.1016/j.ajhg.2011.07.006 PMC3155199

[5] Abdel-Salam GM, Schaffer AE, Zaki MS, Dixon-Salazar T, Mostafa IS, Afifi HH, Gleeson JG (2012) A homozygous IER3IP1 mutation causes microcephaly with simplified gyral pattern, epilepsy, and permanent neonatal diabetes syndrome (MEDS). Am J Med Genet A 158A:2788–2796. 10.1002/ajmg.a.35583 22991235 10.1002/ajmg.a.35583 PMC3477270

[6] Shalev SA, Tenenbaum-Rakover Y, Horovitz Y, Paz VP, Ye H, Carmody D, Highland HM, Boerwinkle E, Hanis CL, Muzny DM, Gibbs RA, Bell GI, Philipson LH, Greeley SA (2014) Microcephaly, epilepsy, and neonatal diabetes due to compound heterozygous mutations in IER3IP1: insights into the natural history of a rare disorder. Pediatr Diabetes 15:252–256. 10.1111/pedi.12086 24138066 10.1111/pedi.12086 PMC3994177

[7] Valenzuela I, Boronat S, Martinez-Saez E, Clemente M, Sanchez-Montanez A, Munell F, Carrascosa A, Macaya A (2017) Microcephaly with simplified gyral pattern, epilepsy and permanent neonatal diabetes syndrome (MEDS). A new patient and review of the literature. Eur J Med Genet 60:517–520. 10.1016/j.ejmg.2017.07.007 28711742 10.1016/j.ejmg.2017.07.007

[8] Sobu E, Kaya Ozcora GD, Yilmaz Gulec E, Sahinoglu B, Tahmiscioglu Bucak F (2022) A New Variant of the IER3IP1 Gene: The First Case of Microcephaly, Epilepsy, and Diabetes Syndrome 1 from Turkey. J Clin Res Pediatr Endocrinol. 10.4274/jcrpe.galenos.2022.2022-8-12 36416459 10.4274/jcrpe.galenos.2022.2022-8-12 PMC11590771

[9] Yiu WH, Poon JWM, Tsui SKW, Fung KP, Waye MMY (2004) Cloning and characterization of a novel endoplasmic reticulum localized G-patch domain protein, IER3IP1. Gene 337:37–44. 10.1016/j.gene.2004.04.033 15276200 10.1016/j.gene.2004.04.033

[10] Heidtman M, Chen CZ, Collins RN, Barlowe C (2005) Yos1p is a novel subunit of the Yip1p-Yif1p complex and is required for transport between the endoplasmic reticulum and the Golgi complex. Mol Biol Cell 16:1673–1683. 10.1091/mbc.E04-10-0873 15659647 10.1091/mbc.E04-10-0873 PMC1073651

[11] De Franco E, Lytrivi M, Ibrahim H, Montaser H, Wakeling MN, Fantuzzi F, Patel K, Demarez C, Cai Y, Igoillo-Esteve M, Cosentino C, Lithovius V, Vihinen H, Jokitalo E, Laver TW, Johnson MB, Sawatani T, Shakeri H, Pachera N, Haliloglu B, Ozbek MN, Unal E, Yildirim R, Godbole T, Yildiz M, Aydin B, Bilheu A, Suzuki I, Flanagan SE, Vanderhaeghen P, Senee V, Julier C, Marchetti P, Eizirik DL, Ellard S, Saarimaki-Vire J, Otonkoski T, Cnop M, Hattersley AT (2020) YIPF5 mutations cause neonatal diabetes and microcephaly through endoplasmic reticulum stress. J Clin Investig 130:6338–6353. 10.1172/Jci141455 33164986 10.1172/JCI141455 PMC7685733

[12] AlMuhaizea M, AlMass R, AlHargan A, AlBader A, Medico Salsench E, Howaidi J, Ihinger J, Karachunski P, Begtrup A, Segura Castell M, Bauer P, Bertoli-Avella A, Kaya IH, AlSufayan J, AlQuait L, Chedrawi A, Arold ST, Colak D, Barakat TS, Kaya N (2020) Truncating mutations in YIF1B cause a progressive encephalopathy with various degrees of mixed movement disorder, microcephaly, and epilepsy. Acta Neuropathol 139:791–794. 10.1007/s00401-020-02128-8 32006098 10.1007/s00401-020-02128-8

[13] Sun J, Ren D (2017) IER3IP1 deficiency leads to increased beta-cell death and decreased beta-cell proliferation. Oncotarget 8(34):56768–56779 28915629 10.18632/oncotarget.18179 PMC5593600

[14] Yang J, Zhen J, Feng W, Fan Z, Ding L, Yang X, Huang Y, Shu H, Xie J, Li X, Qiao J, Fan Y, Sun J, Li N, Liu T, Wang S, Zhang X, Arvan P, Liu M (2022) IER3IP1 is critical for maintaining glucose homeostasis through regulating the endoplasmic reticulum function and survival of beta cells. Proc Natl Acad Sci U S A 119:e2204443119. 10.1073/pnas.2204443119 36322741 10.1073/pnas.2204443119 PMC9659391

[15] Esk C, Lindenhofer D, Haendeler S, Wester RA, Pflug F, Schroeder B, Bagley JA, Elling U, Zuber J, von Haeseler A, Knoblich JA (2020) A human tissue screen identifies a regulator of ER secretion as a brain-size determinant. Science 370:935–941. 10.1126/science.abb5390 33122427 10.1126/science.abb5390

[16] Shomron O, Nevo-Yassaf I, Aviad T, Yaffe Y, Zahavi EE, Dukhovny A, Perlson E, Brodsky I, Yeheskel A, Pasmanik-Chor M, Mironov A, Beznoussenko GV, Mironov AA, Sklan EH, Patterson GH, Yonemura Y, Sannai M, Kaether C, Hirschberg K (2021) COPII collar defines the boundary between ER and ER exit site and does not coat cargo containers. J Cell Biol 220(6):e201907224 33852719 10.1083/jcb.201907224 PMC8054201

[17] Visser JJ, Cheng Y, Perry SC, Chastain AB, Parsa B, Masri SS, Ray TA, Kay JN, Wojtowicz WM (2015) An extracellular biochemical screen reveals that FLRTs and Unc5s mediate neuronal subtype recognition in the retina. Elife 2(4):e08149 10.7554/eLife.08149 PMC4737655 26633812

[18] Hung V, Lam SS, Udeshi ND, Svinkina T, Guzman G, Mootha VK, Carr SA, Ting AY (2017) Proteomic mapping of cytosol-facing outer mitochondrial and ER membranes in living human cells by proximity biotinylation. Elife 6:24463 10.7554/eLife.24463 PMC5404927 28441135

[19] Boncompain G, Divoux S, Gareil N, de Forges H, Lescure A, Latreche L, Mercanti V, Jollivet F, Raposo G, Perez F (2012) Synchronization of secretory protein traffic in populations of cells. Nat Methods 9:493–498. 10.1038/nmeth.1928 22406856 10.1038/nmeth.1928

[20] Hageman J, Kampinga HH (2009) Computational analysis of the human HSPH/HSPA/DNAJ family and cloning of a human HSPH/HSPA/DNAJ expression library. Cell Stress Chaperones 14:1–21. 10.1007/s12192-008-0060-2 18686016 10.1007/s12192-008-0060-2 PMC2673897

[21] Ran FA, Hsu PD, Wright J, Agarwala V, Scott DA, Zhang F (2013) Genome engineering using the CRISPR-Cas9 system. Nat Protoc 8:2281–2308. 10.1038/nprot.2013.143 24157548 10.1038/nprot.2013.143 PMC3969860

[22] Snapp EL, Sharma A, Lippincott-Schwartz J, Hegde RS (2006) Monitoring chaperone engagement of substrates in the endoplasmic reticulum of live cells. Proc Natl Acad Sci U S A 103:6536–6541. 10.1073/pnas.0510657103 16617114 10.1073/pnas.0510657103 PMC1458919

[23] Wang J, Sun Q, Morita Y, Jiang H, Gross A, Lechel A, Hildner K, Guachalla LM, Gompf A, Hartmann D, Schambach A, Wuestefeld T, Dauch D, Schrezenmeier H, Hofmann WK, Nakauchi H, Ju Z, Kestler HA, Zender L, Rudolph KL (2012) A differentiation checkpoint limits hematopoietic stem cell self-renewal in response to DNA damage. Cell 148:1001–1014. 10.1016/j.cell.2012.01.040 22385964 10.1016/j.cell.2012.01.040

[24] Tapia D., Jimenez T., Zamora C., Espinoza J., Rizzo R., Gonzalez-Cardenas A., Fuentes D., Hernandez S., Cavieres V. A., Soza A., Guzman F., Arriagada G., Yuseff M. I., Mardones G. A., Burgos P. V., Luini A., Gonzalez A., and Cancino J (2019) KDEL receptor regulates secretion by lysosome relocation- and autophagy-dependent modulation of lipid-droplet turnover. *Nat Commun* 1010.1038/s41467–019–08501-w 10.1038/s41467-019-08501-w PMC6374470 30760704

[25] Ben-Tekaya H, Miura K, Pepperkok R, Hauri HP (2005) Live imaging of bidirectional traffic from the ERGIC. J Cell Sci 118:357–367. 10.1242/jcs.01615 15632110 10.1242/jcs.01615

[26] Gudernova I, Foldynova-Trantirkova S, El Ghannamova B, Fafilek B, Varecha M, Balek L, Hruba E, Jonatova L, Jelinkova I, Kunova Bosakova M, Trantirek L, Mayer J, Krejci P (2017) One reporter for incell activity profiling of majority of protein kinase oncogenes. Elife 15(6):21536 10.7554/eLife.21536 PMC5310841 28199182

[27] Raissi AJ, Staudenmaier EK, David S, Hu LD, Paradis S (2013) Sema4D localizes to synapses and regulates GABAergic synapse development as a membrane-bound molecule in the mammalian hippocampus. Mol Cell Neurosci 57:23–32. 10.1016/j.mcn.2013.08.004 24036351 10.1016/j.mcn.2013.08.004 PMC3873869

[28] Labun K, Montague TG, Krause M, Torres Cleuren YN, Tjeldnes H, Valen E (2019) CHOPCHOP v3: expanding the CRISPR web toolbox beyond genome editing. Nucleic Acids Res 47:W171–W174. 10.1093/nar/gkz365 31106371 10.1093/nar/gkz365 PMC6602426

[29] Schindelin J, Arganda-Carreras I, Frise E, Kaynig V, Longair M, Pietzsch T, Preibisch S, Rueden C, Saalfeld S, Schmid B, Tinevez JY, White DJ, Hartenstein V, Eliceiri K, Tomancak P, Cardona A (2012) Fiji: an open-source platform for biological-image analysis. Nat Methods 9:676–682. 10.1038/nmeth.2019 22743772 10.1038/nmeth.2019 PMC3855844

[30] Stirling DR, Swain-Bowden MJ, Lucas AM, Carpenter AE, Cimini BA, Goodman A (2021) Cell profiler 4: improvements in speed, utility and usability. BMC Bioinform 22:433. 10.1186/s12859-021-04344-9 10.1186/s12859-021-04344-9 PMC8431850 34507520

[31] Richardson KC, Jarett L, Finke EH (1960) Embedding in epoxy resins for ultrathin sectioning in electron microscopy. Stain Technol 35:313–323. 10.3109/10520296009114754 13741297 10.3109/10520296009114754

[32] Mizushima N, Murphy LO (2020) Autophagy assays for biological discovery and therapeutic development. Trends Biochem Sci 45:1080–1093. 10.1016/j.tibs.2020.07.006 32839099 10.1016/j.tibs.2020.07.006

[33] Serdaroglu A, Muller SA, Schepers U, Brase S, Weichert W, Lichtenthaler SF, Kuhn PH (2017) An optimised version of the secretome protein enrichment with click sugars (SPECS) method leads to enhanced coverage of the secretome. Proteomics 17(5):1600423 10.1002/pmic.201600423 27991726

[34] Fernandopulle MS, Prestil R, Grunseich C, Wang C, Gan L, Ward ME (2018) Transcription factor-mediated differentiation of human iPSCs into neurons. Curr Protoc Cell Biol 79:e51. 10.1002/cpcb.51 29924488 10.1002/cpcb.51 PMC6993937

[35] Tian R, Gachechiladze MA, Ludwig CH, Laurie MT, Hong JY, Nathaniel D, Prabhu AV, Fernandopulle MS, Patel R, Abshari M, Ward ME, Kampmann M (2019) CRISPR Interference-based platform for multimodal genetic screens in human iPSC-derived neurons. Neuron 104(239–255):e212. 10.1016/j.neuron.2019.07.014 10.1016/j.neuron.2019.07.014 PMC6813890 31422865

[36] Pelossof R, Fairchild L, Huang CH, Widmer C, Sreedharan VT, Sinha N, Lai DY, Guan Y, Premsrirut PK, Tschaharganeh DF, Hoffmann T, Thapar V, Xiang Q, Garippa RJ, Ratsch G, Zuber J, Lowe SW, Leslie CS, Fellmann C (2017) Prediction of potent shRNAs with a sequential classification algorithm. Nat Biotechnol 35:350–353. 10.1038/nbt.3807 28263295 10.1038/nbt.3807 PMC5416823

[37] Fellmann C, Hoffmann T, Sridhar V, Hopfgartner B, Muhar M, Roth M, Lai DY, Barbosa IA, Kwon JS, Guan Y, Sinha N, Zuber J (2013) An optimized microRNA backbone for effective single-copy RNAi. Cell Rep 5:1704–1713. 10.1016/j.celrep.2013.11.020 24332856 10.1016/j.celrep.2013.11.020

[38] Mestres I., and Calegari F. (2022) 4931414P19Rik: A Chemoattractant Secreted by Neural Progenitors Modulates Microglia Activation and Neuronal Migration During Mammalian Brain Development. bioRxiv 10.1101/2022.12.22.521648 10.1242/dev.201574 PMC10163356 37070770

[39] Martinez-Martinez MA, Ciceri G, Espinos A, Fernandez V, Marin O, Borrell V (2019) Extensive branching of radially-migrating neurons in the mammalian cerebral cortex. J Comp Neurol 527:1558–1576. 10.1002/cne.24597 30520050 10.1002/cne.24597

[40] Jumper J, Evans R, Pritzel A, Green T, Figurnov M, Ronneberger O, Tunyasuvunakool K, Bates R, Zidek A, Potapenko A, Bridgland A, Meyer C, Kohl SAA, Ballard AJ, Cowie A, Romera-Paredes B, Nikolov S, Jain R, Adler J, Back T, Petersen S, Reiman D, Clancy E, Zielinski M, Steinegger M, Pacholska M, Berghammer T, Bodenstein S, Silver D, Vinyals O, Senior AW, Kavukcuoglu K, Kohli P, Hassabis D (2021) Highly accurate protein structure prediction with AlphaFold. Nature. 10.1038/s41586-021-03819-2 34293799 10.1038/s41586-021-03828-1 PMC8387240

[41] Aridor M, Bannykh SI, Rowe T, Balch WE (1995) Sequential coupling between COPII and COPI vesicle coats in endoplasmic reticulum to golgi transport. J Cell Biol 131:875–893 7490291 10.1083/jcb.131.4.875 PMC2200014

[42] Zanetti G, Pahuja KB, Studer S, Shim S, Schekman R (2011) COPII and the regulation of protein sorting in mammals. Nat Cell Biol 14:20–28. 10.1038/ncb2390 22193160 10.1038/ncb2390

[43] Lippincott-Schwartz J, Yuan LC, Bonifacino JS, Klausner RD (1989) Rapid redistribution of Golgi proteins into the ER in cells treated with brefeldin A: evidence for membrane cycling from Golgi to ER. Cell 56:801–813. 10.1016/0092-8674(89)90685-5 2647301 10.1016/0092-8674(89)90685-5 PMC7173269

[44] Nyfeler B, Reiterer V, Wendeler MW, Stefan E, Zhang B, Michnick SW, Hauri HP (2008) Identification of ERGIC-53 as an intracellular transport receptor of alpha1-antitrypsin. J Cell Biol 180:705–712. 10.1083/jcb.200709100 18283111 10.1083/jcb.200709100 PMC2265576

[45] Fregno I, Molinari M (2019) Proteasomal and lysosomal clearance of faulty secretory proteins: ER-associated degradation (ERAD) and ER-to-lysosome-associated degradation (ERLAD) pathways. Crit Rev Biochem Mol Biol 54:153–163. 10.1080/10409238.2019.1610351 31084437 10.1080/10409238.2019.1610351

[46] Anelli T, Sitia R (2008) Protein quality control in the early secretory pathway. EMBO J 27:315–327. 10.1038/sj.emboj.7601974 18216874 10.1038/sj.emboj.7601974 PMC2234347

[47] Lewis MJ, Pelham HR (1990) A human homologue of the yeast HDEL receptor. Nature 348:162–163. 10.1038/348162a0 2172835 10.1038/348162a0

[48] Raykhel I, Alanen H, Salo K, Jurvansuu J, Nguyen VD, Latva-Ranta M, Ruddock L (2007) A molecular specificity code for the three mammalian KDEL receptors. J Cell Biol 179:1193–1204. 10.1083/jcb.200705180 18086916 10.1083/jcb.200705180 PMC2140024

[49] Lewis MJ, Sweet DJ, Pelham HR (1990) The ERD2 gene determines the specificity of the luminal ER protein retention system. Cell 61:1359–1363. 10.1016/0092-8674(90)90699-f 2194671 10.1016/0092-8674(90)90699-f

[50] Korkhov VM, Milan-Lobo L, Zuber B, Farhan H, Schmid JA, Freissmuth M, Sitte HH (2008) Peptide-based interactions with calnexin target misassembled membrane proteins into endoplasmic reticulum-derived multilamellar bodies. J Mol Biol 378:337–352. 10.1016/j.jmb.2008.02.056 18367207 10.1016/j.jmb.2008.02.056 PMC4493858

[51] Snapp EL, Hegde RS, Francolini M, Lombardo F, Colombo S, Pedrazzini E, Borgese N, Lippincott-Schwartz J (2003) Formation of stacked ER cisternae by low affinity protein interactions. J Cell Biol 163:257–269. 10.1083/jcb.200306020 14581454 10.1083/jcb.200306020 PMC2173526

[52] Xu F, Du WQ, Zou Q, Wang YT, Zhang X, Xing XD, Li Y, Zhang DC, Wang HM, Zhang WH, Hu XY, Liu X, Liu XL, Zhang SJ, Yu JQ, Fang JH, Li FJ, Zhou Y, Yue TQ, Mi N, Deng HT, Zou P, Chen XW, Yang XR, Yu L (2021) COPII mitigates ER stress by promoting formation of ER whorls. Cell Res 31:141–156. 10.1038/s41422-020-00416-2 32989223 10.1038/s41422-020-00416-2 PMC8026990

[53] Wang C, Ward ME, Chen R, Liu K, Tracy TE, Chen X, Xie M, Sohn PD, Ludwig C, Meyer-Franke A, Karch CM, Ding S, Gan L (2017) Scalable production of iPSC-derived human neurons to identify tau-lowering compounds by high-content screening. Stem Cell Rep 9:1221–1233. 10.1016/j.stemcr.2017.08.019 10.1016/j.stemcr.2017.08.019 PMC5639430 28966121

[54] Simon R, Brylka H, Schwegler H, Venkataramanappa S, Andratschke J, Wiegreffe C, Liu P, Fuchs E, Jenkins NA, Copeland NG, Birchmeier C, Britsch S (2012) A dual function of Bcl11b/Ctip2 in hippocampal neurogenesis. EMBO J 31:2922–2936. 10.1038/emboj.2012.142 22588081 10.1038/emboj.2012.142 PMC3395096

[55] Singhal A, Morris VB, Labhasetwar V, Ghorpade A (2013) Nanoparticle-mediated catalase delivery protects human neurons from oxidative stress. Cell Death Dis 4:e903. 10.1038/cddis.2013.362 24201802 10.1038/cddis.2013.362 PMC3847304

[56] Qiu S, Wu Q, Wang H, Liu D, Chen C, Zhu Z, Zheng H, Yang G, Li L, Yang M (2024) AZGP1 in POMC neurons modulates energy homeostasis and metabolism through leptin-mediated STAT3 phosphorylation. Nat Commun 15:3377. 10.1038/s41467-024-47684-9 38643150 10.1038/s41467-024-47684-9 PMC11032411

[57] Weyer SW, Klevanski M, Delekate A, Voikar V, Aydin D, Hick M, Filippov M, Drost N, Schaller KL, Saar M, Vogt MA, Gass P, Samanta A, Jaschke A, Korte M, Wolfer DP, Caldwell JH, Muller UC (2011) APP and APLP2 are essential at PNS and CNS synapses for transmission, spatial learning and LTP. EMBO J 30:2266–2280. 10.1038/emboj.2011.119 21522131 10.1038/emboj.2011.119 PMC3117640

[58] Bouhouche A, Benomar A, Bouslam N, Chkili T, Yahyaoui M (2006) Mutation in the epsilon subunit of the cytosolic chaperonin-containing t-complex peptide-1 (Cct5) gene causes autosomal recessive mutilating sensory neuropathy with spastic paraplegia. J Med Genet 43:441–443. 10.1136/jmg.2005.039230 16399879 10.1136/jmg.2005.039230 PMC2564519

[59] Tabata H, Nakajima K (2003) Multipolar migration: the third mode of radial neuronal migration in the developing cerebral cortex. J Neurosci 23:9996–10001. 10.1523/JNEUROSCI.23-31-09996.2003 14602813 10.1523/JNEUROSCI.23-31-09996.2003 PMC6740853

[60] Noctor SC, Martinez-Cerdeno V, Ivic L, Kriegstein AR (2004) Cortical neurons arise in symmetric and asymmetric division zones and migrate through specific phases. Nat Neurosci 7:136–144. 10.1038/nn1172 14703572 10.1038/nn1172

[61] Kon E, Cossard A, Jossin Y (2017) Neuronal polarity in the embryonic mammalian cerebral cortex. Front Cell Neurosci 11:163. 10.3389/fncel.2017.00163 28670267 10.3389/fncel.2017.00163 PMC5472699

[62] Leshchyns’ka I, Sytnyk V (2016) Reciprocal interactions between cell adhesion molecules of the immunoglobulin superfamily and the cytoskeleton in neurons. Front Cell Dev Biol 4:9. 10.3389/fcell.2016.00009 26909348 10.3389/fcell.2016.00009 PMC4754453

[63] Belden WJ, Barlowe C (2001) Role of Erv29p in collecting soluble secretory proteins into ER-derived transport vesicles. Science 294:1528–1531. 10.1126/science.1065224 11711675 10.1126/science.1065224

[64] Appenzeller-Herzog C, Nyfeler B, Burkhard P, Santamaria I, Lopez-Otin C, Hauri HP (2005) Carbohydrate- and conformation-dependent cargo capture for ER-exit. Mol Biol Cell 16:1258–1267. 10.1091/mbc.e04-08-0708 15635097 10.1091/mbc.E04-08-0708 PMC551490

[65] Powers J, Barlowe C (1998) Transport of axl2p depends on erv14p, an ER-vesicle protein related to the Drosophila cornichon gene product. J Cell Biol 142:1209–1222. 10.1083/jcb.142.5.1209 9732282 10.1083/jcb.142.5.1209 PMC2149358

[66] Muller L, Zhu X, Lindberg I (1997) Mechanism of the facilitation of PC2 maturation by 7B2: involvement in ProPC2 transport and activation but not folding. J Cell Biol 139:625–638. 10.1083/jcb.139.3.625 9348280 10.1083/jcb.139.3.625 PMC2141705

[67] Rafiullah R, Aslamkhan M, Paramasivam N, Thiel C, Mustafa G, Wiemann S, Schlesner M, Wade RC, Rappold GA, Berkel S (2016) Homozygous missense mutation in the LMAN2L gene segregates with intellectual disability in a large consanguineous Pakistani family. J Med Genet 53:138–144. 10.1136/jmedgenet-2015-103179 26566883 10.1136/jmedgenet-2015-103179

[68] Semenza JC, Hardwick KG, Dean N, Pelham HR (1990) ERD2, a yeast gene required for the receptor-mediated retrieval of luminal ER proteins from the secretory pathway. Cell 61:1349–1357. 10.1016/0092-8674(90)90698-e 2194670 10.1016/0092-8674(90)90698-e

[69] Scales SJ, Pepperkok R, Kreis TE (1997) Visualization of ER-to-Golgi transport in living cells reveals a sequential mode of action for COPII and COPI. Cell 90:1137–1148. 10.1016/s0092-8674(00)80379-7 9323141 10.1016/s0092-8674(00)80379-7

[70] Li MY, Grandadam M, Kwok K, Lagache T, Siu YL, Zhang JS, Sayteng K, Kudelko M, Qin CF, Olivo-Marin JC, Bruzzone R, Wang PG (2015) KDEL receptors assist dengue virus exit from the endoplasmic reticulum. Cell Rep 10:1496–1507. 10.1016/j.celrep.2015.02.021 25753416 10.1016/j.celrep.2015.02.021

[71] Blum A, Khalifa S, Nordstrom K, Simon M, Schulz MH, Schmitt MJ (2019) Transcriptomics of a KDELR1 knockout cell line reveals modulated cell adhesion properties. Sci Rep 9:10611. 10.1038/s41598-019-47027-5 31337861 10.1038/s41598-019-47027-5 PMC6650600

[72] Efthymiou S, Herman I, Rahman F, Anwar N, Maroofian R, Yip J, Mitani T, Calame DG, Hunter JV, Sutton VR, Yilmaz GE (2021) Two novel bi-allelic KDELR2 missense variants cause osteogenesis imperfecta with neurodevelopmental features. Am J Med Genet Part A 185(7):2241–2249. 10.1002/ajmg.a.62221 33964184 10.1002/ajmg.a.62221 PMC8436746

[73] Gomez-Navarro N, Melero A, Li XH, Boulanger J, Kukulski W, Miller EA (2020) Cargo crowding contributes to sorting stringency in COPII vesicles. J Cell Biol. 10.1083/jcb.201806038 32406500 10.1083/jcb.201806038 PMC7300426

[74] Booth C, Koch GL (1989) Perturbation of cellular calcium induces secretion of luminal ER proteins. Cell 59:729–737. 10.1016/0092-8674(89)90019-6 2510935 10.1016/0092-8674(89)90019-6

[75] Yamamoto K, Fujii R, Toyofuku Y, Saito T, Koseki H, Hsu VW, Aoe T (2001) The KDEL receptor mediates a retrieval mechanism that contributes to quality control at the endoplasmic reticulum. EMBO J 20:3082–3091. 10.1093/emboj/20.12.3082 11406585 10.1093/emboj/20.12.3082 PMC150210

[76] Mimura N, Yuasa S, Soma M, Jin H, Kimura K, Goto S, Koseki H, Aoe T (2008) Altered quality control in the endoplasmic reticulum causes cortical dysplasia in knock-in mice expressing a mutant BiP. Mol Cell Biol 28:293–301. 10.1128/MCB.00473-07 17954555 10.1128/MCB.00473-07 PMC2223281

[77] Inaguma Y, Hamada N, Tabata H, Iwamoto I, Mizuno M, Nishimura YV, Ito H, Morishita R, Suzuki M, Ohno K, Kumagai T, Nagata K (2014) SIL1, a causative cochaperone gene of Marinesco-Sojgren syndrome, plays an essential role in establishing the architecture of the developing cerebral cortex. EMBO Mol Med 6:414–429. 10.1002/emmm.201303069 24473200 10.1002/emmm.201303069 PMC3958314

[78] Takeuchi H, Wong D, Schneider M, Freeze HH, Takeuchi M, Berardinelli SJ, Ito A, Lee H, Nelson SF, Haltiwanger RS (2018) Variant in human POFUT1 reduces enzymatic activity and likely causes a recessive microcephaly, global developmental delay with cardiac and vascular features. Glycobiology 28:276–283. 10.1093/glycob/cwy014 29452367 10.1093/glycob/cwy014 PMC6057529

[79] Ucuncu E, Rajamani K, Wilson MSC, Medina-Cano D, Altin N, David P, Barcia G, Lefort N, Banal C, Vasilache-Dangles MT, Pitelet G, Lorino E, Rabasse N, Bieth E, Zaki MS, Topcu M, Sonmez FM, Musaev D, Stanley V, Bole-Feysot C, Nitschke P, Munnich A, Bahi-Buisson N, Fossoud C, Giuliano F, Colleaux L, Burglen L, Gleeson JG, Boddaert N, Saiardi A, Cantagrel V (2020) MINPP1 prevents intracellular accumulation of the chelator inositol hexakisphosphate and is mutated in Pontocerebellar Hypoplasia. Nat Commun 11:6087. 10.1038/s41467-020-19919-y 33257696 10.1038/s41467-020-19919-y PMC7705663

[80] Long KR, Huttner WB (2019) How the extracellular matrix shapes neural development. Open Biol 9:180216. 10.1098/rsob.180216 30958121 10.1098/rsob.180216 PMC6367132

[81] Gray AJ, Park PW, Broekelmann TJ, Laurent GJ, Reeves JT, Stenmark KR, Mecham RP (1995) The mitogenic effects of the B beta chain of fibrinogen are mediated through cell surface calreticulin. J Biol Chem 270:26602–26606. 10.1074/jbc.270.44.26602 7592883 10.1074/jbc.270.44.26602

[82] White TK, Zhu Q, Tanzer ML (1995) Cell surface calreticulin is a putative mannoside lectin which triggers mouse melanoma cell spreading. J Biol Chem 270:15926–15929. 10.1074/jbc.270.27.15926 7608143 10.1074/jbc.270.27.15926

[83] Fukawa M, Shirai R, Torii T, Nakata K, Fukatsu S, Sato T, Homma K, Miyamoto Y, Yamauchi J (2023) Extracellular HSPA5 is autocrinally involved in the regulation of neuronal process elongation. Biochem Biophys Res Commun 664:50–58. 10.1016/j.bbrc.2023.04.102 37137223 10.1016/j.bbrc.2023.04.102

[84] Wilson DG, Phamluong K, Li L, Sun M, Cao TC, Liu PS, Modrusan Z, Sandoval WN, Rangell L, Carano RA, Peterson AS, Solloway MJ (2011) Global defects in collagen secretion in a Mia3/TANGO1 knockout mouse. J Cell Biol 193:935–951. 10.1083/jcb.201007162 21606205 10.1083/jcb.201007162 PMC3105544

[85] Lu CL, Ortmeier S, Brudvig J, Moretti T, Cain J, Boyadjiev SA, Weimer JM, Kim J (2022) Collagen has a unique SEC24 preference for efficient export from the endoplasmic reticulum. Traffic 23:81–93. 10.1111/tra.12826 34761479 10.1111/tra.12826 PMC8692420

[86] Tao J, Zhu M, Wang H, Afelik S, Vasievich MP, Chen XW, Zhu G, Jensen J, Ginsburg D, Zhang B (2012) SEC23B is required for the maintenance of murine professional secretory tissues. Proc Natl Acad Sci U S A 109:E2001-2009. 10.1073/pnas.1209207109 22745161 10.1073/pnas.1209207109 PMC3406820

[87] Liu Z, Yan M, Lei W, Jiang R, Dai W, Chen J, Wang C, Li L, Wu M, Nian X, Li D, Sun D, Lv X, Wang C, Xie C, Yao L, Wu C, Hu J, Xiao N, Mo W, Wang Z, Zhang L (2022) Sec13 promotes oligodendrocyte differentiation and myelin repair through autocrine pleiotrophin signaling. J Clin Investig. 10.1172/JCI155096 35143418 10.1172/JCI155096 PMC8970680

[88] Cutrona MB, Beznoussenko GV, Fusella A, Martella O, Moral P, Mironov AA (2013) Silencing of mammalian Sar1 isoforms reveals COPII-independent protein sorting and transport. Traffic 14:691–708. 10.1111/tra.12060 23433038 10.1111/tra.12060

[89] Taverna E, Gotz M, Huttner WB (2014) The cell biology of neurogenesis: toward an understanding of the development and evolution of the neocortex. Annu Rev Cell Dev Biol 30:465–502. 10.1146/annurev-cellbio-101011-155801 25000993 10.1146/annurev-cellbio-101011-155801

[90] Aldinger KA, Mosca SJ, Tétreault M, Dempsey JC, Ishak GE, Hartley T, Phelps IG, Lamont RE, O’Day DR, Basel D, Gripp KW (2014) Mutations in LAMA1 cause cerebellar dysplasia and cysts with and without retinal dystrophy. The Am J Human Genet 95(227):234. 10.1016/j.ajhg.2014.07.007 10.1016/j.ajhg.2014.07.007 PMC4129402 25105227

[91] Namba T, Kibe Y, Funahashi Y, Nakamuta S, Takano T, Ueno T, Shimada A, Kozawa S, Okamoto M, Shimoda Y, Oda K, Wada Y, Masuda T, Sakakibara A, Igarashi M, Miyata T, Faivre-Sarrailh C, Takeuchi K, Kaibuchi K (2014) Pioneering axons regulate neuronal polarization in the developing cerebral cortex. Neuron 81:814–829. 10.1016/j.neuron.2013.12.015 24559674 10.1016/j.neuron.2013.12.015

[92] Leone DP, Relvas JB, Campos LS, Hemmi S, Brakebusch C, Fassler R, Ffrench-Constant C, Suter U (2005) Regulation of neural progenitor proliferation and survival by beta1 integrins. J Cell Sci 118:2589–2599. 10.1242/jcs.02396 15928047 10.1242/jcs.02396

[93] Chen ZL, Haegeli V, Yu H, Strickland S (2009) Cortical deficiency of laminin gamma1 impairs the AKT/GSK-3beta signaling pathway and leads to defects in neurite outgrowth and neuronal migration. Dev Biol 327:158–168. 10.1016/j.ydbio.2008.12.006 19118544 10.1016/j.ydbio.2008.12.006 PMC2669444

[94] Hasegawa H, Ashigaki S, Takamatsu M, Suzuki-Migishima R, Ohbayashi N, Itoh N, Takada S, Tanabe Y (2004) Laminar patterning in the developing neocortex by temporally coordinated fibroblast growth factor signaling. J Neurosci 24:8711–8719. 10.1523/JNEUROSCI.3070-04.2004 15470137 10.1523/JNEUROSCI.3070-04.2004 PMC6729962

[95] Szczurkowska J, Pischedda F, Pinto B, Manago F, Haas CA, Summa M, Bertorelli R, Papaleo F, Schafer MK, Piccoli G, Cancedda L (2018) NEGR1 and FGFR2 cooperatively regulate cortical development and core behaviours related to autism disorders in mice. Brain 141:2772–2794. 10.1093/brain/awy190 30059965 10.1093/brain/awy190 PMC6113639

[96] Sun X, Zhang R, Chen H, Du X, Chen S, Huang J, Liu M, Xu M, Luo F, Jin M, Su N, Qi H, Yang J, Tan Q, Zhang D, Ni Z, Liang S, Zhang B, Chen D, Zhang X, Luo L, Chen L, Xie Y (2020) Fgfr3 mutation disrupts chondrogenesis and bone ossification in zebrafish model mimicking CATSHL syndrome partially via enhanced Wnt/beta-catenin signaling. Theranostics 10:7111–7130. 10.7150/thno.45286 32641982 10.7150/thno.45286 PMC7330844

[97] Dambroise E, Ktorza I, Brombin A, Abdessalem G, Edouard J, Luka M, Fiedler I, Binder O, Pelle O, Patton EE, Busse B, Menager M, Sohm F, Legeai-Mallet L (2020) Fgfr3 Is a positive regulator of osteoblast expansion and differentiation during zebrafish skull vault development. J Bone Miner Res 35:1782–1797. 10.1002/jbmr.4042 32379366 10.1002/jbmr.4042

[98] Toydemir RM, Brassington AE, Bayrak-Toydemir P, Krakowiak PA, Jorde LB, Whitby FG, Longo N, Viskochil DH, Carey JC, Bamshad MJ (2006) A novel mutation in FGFR3 causes camptodactyly, tall stature, and hearing loss (CATSHL) syndrome. Am J Hum Genet 79:935–941. 10.1086/508433 17033969 10.1086/508433 PMC1698566

[99] Meyers EA, Kessler JA (2017) TGF-beta family signaling in neural and neuronal differentiation development and function. Cold Spring Harb Perspect Biol 9(8):a022244 28130363 10.1101/cshperspect.a022244 PMC5538418

[100] Hong K, Hinck L, Nishiyama M, Poo MM, Tessier-Lavigne M, Stein E (1999) A ligand-gated association between cytoplasmic domains of UNC5 and DCC family receptors converts netrin-induced growth cone attraction to repulsion. Cell 97:927–941. 10.1016/s0092-8674(00)80804-1 10399920 10.1016/s0092-8674(00)80804-1

[101] Serafini T, Colamarino SA, Leonardo ED, Wang H, Beddington R, Skarnes WC, Tessier-Lavigne M (1996) Netrin-1 is required for commissural axon guidance in the developing vertebrate nervous system. Cell 87:1001–1014. 10.1016/s0092-8674(00)81795-x 8978605 10.1016/s0092-8674(00)81795-x

[102] Leonardo ED, Hinck L, Masu M, Keino-Masu K, Ackerman SL, Tessier-Lavigne M (1997) Vertebrate homologues of C. elegans UNC-5 are candidate netrin receptors. Nature 386:833–838. 10.1038/386833a0 9126742 10.1038/386833a0

[103] van den Berghe V, Stappers E, Vandesande B, Dimidschstein J, Kroes R, Francis A, Conidi A, Lesage F, Dries R, Cazzola S, Berx G, Kessaris N, Vanderhaeghen P, van Ijcken W, Grosveld FG, Goossens S, Haigh JJ, Fishell G, Goffinet A, Aerts S, Huylebroeck D, Seuntjens E (2013) Directed migration of cortical interneurons depends on the cell-autonomous action of Sip1. Neuron 77:70–82. 10.1016/j.neuron.2012.11.009 23312517 10.1016/j.neuron.2012.11.009

[104] Ahn EH, Kang SS, Qi Q, Liu X, Ye K (2020) Netrin1 deficiency activates MST1 via UNC5B receptor, promoting dopaminergic apoptosis in Parkinson’s disease. Proc Natl Acad Sci U S A 117:24503–24513. 10.1073/pnas.2004087117 32929029 10.1073/pnas.2004087117 PMC7533679

[105] Tang X, Jang SW, Okada M, Chan CB, Feng Y, Liu Y, Luo SW, Hong Y, Rama N, Xiong WC, Mehlen P, Ye K (2008) Netrin-1 mediates neuronal survival through PIKE-L interaction with the dependence receptor UNC5B. Nat Cell Biol 10:698–706. 10.1038/ncb1732 18469807 10.1038/ncb1732 PMC2839190

[106] Shen CY, Chang YC, Chen LH, Lin WC, Lee YH, Yeh ST, Chen HK, Fang W, Hsu CP, Lee JM, Lu TP, Hsiao PW, Lai LC, Tsai MH, Chuang EY (2018) The extracellular SEMA domain attenuates intracellular apoptotic signaling of semaphorin 6A in lung cancer cells. Oncogenesis 7:95. 10.1038/s41389-018-0105-z 30518871 10.1038/s41389-018-0105-z PMC6281666

[107] Park HJ, Kim Y, Kim MK, Kim HJ, Bae SK, Bae MK (2023) Inhibition of the Semaphorin 4D-Plexin-B1 axis prevents calcification in vascular smooth muscle cells. BMB Rep 56:160–165. 10.5483/BMBRep.2022-0165 36443004 10.5483/BMBRep.2022-0165 PMC10068346

[108] Rezaeepoor M, Rashidi G, Pourjafar M, Mohammadi C, Solgi G, Najafi R (2021) SEMA4D knockdown attenuates beta-catenin-dependent tumor progression in colorectal cancer. Biomed Res Int 2021:8507373. 10.1155/2021/8507373 34337054 10.1155/2021/8507373 PMC8321723

[109] Limoni G, Niquille M (2021) Semaphorins and Plexins in central nervous system patterning: the key to it all? Curr Opin Neurobiol 66:224–232. 10.1016/j.conb.2020.12.014 33513538 10.1016/j.conb.2020.12.014

[110] Copf T (2016) Impairments in dendrite morphogenesis as etiology for neurodevelopmental disorders and implications for therapeutic treatments. Neurosci Biobehav Rev 68:946–978. 10.1016/j.neubiorev.2016.04.008 27143622 10.1016/j.neubiorev.2016.04.008

[111] Aligianis IA, Johnson CA, Gissen P, Chen D, Hampshire D, Hoffmann K, Maina EN, Morgan NV, Tee L, Morton J, Ainsworth JR, Horn D, Rosser E, Cole TR, Stolte-Dijkstra I, Fieggen K, Clayton-Smith J, Megarbane A, Shield JP, Newbury-Ecob R, Dobyns WB, Graham JM Jr, Kjaer KW, Warburg M, Bond J, Trembath RC, Harris LW, Takai Y, Mundlos S, Tannahill D, Woods CG, Maher ER (2005) Mutations of the catalytic subunit of RAB3GAP cause Warburg Micro syndrome. Nat Genet 37:221–223. 10.1038/ng1517 15696165 10.1038/ng1517

[112] Wu Q, Sun X, Yue W, Lu T, Ruan Y, Chen T, Zhang D (2016) RAB18, a protein associated with Warburg Micro syndrome, controls neuronal migration in the developing cerebral cortex. Mol Brain 9:19. 10.1186/s13041-016-0198-2 26879639 10.1186/s13041-016-0198-2 PMC4754921

[113] Sheen VL, Ganesh VS, Topcu M, Sebire G, Bodell A, Hill RS, Grant PE, Shugart YY, Imitola J, Khoury SJ, Guerrini R, Walsh CA (2004) Mutations in ARFGEF2 implicate vesicle trafficking in neural progenitor proliferation and migration in the human cerebral cortex. Nat Genet 36:69–76. 10.1038/ng1276 14647276 10.1038/ng1276

[114] de Sainte Agathe JM, Pode-Shakked B, Naudion S, Michaud V, Arveiler B, Fergelot P, Delmas J, Keren B, Poirsier C, Alkuraya FS, Tabarki B, Bend E, Davis K, Bebin M, Thompson ML, Bryant EM, Wagner M, Hannibal I, Lenberg J, Krenn M, Wigby KM, Friedman JR, Iascone M, Cereda A, Miao T, LeGuern E, Argilli E, Sherr E, Caluseriu O, Tidwell T, Bayrak-Toydemir P, Hagedorn C, Brugger M, Vill K, Morneau-Jacob FD, Chung W, Weaver KN, Owens JW, Husami A, Chaudhari BP, Stone BS, Burns K, Li R, de Lange IM, Biehler M, Ginglinger E, Gerard B, Stottmann RW, Trimouille A (2023) ARF1-related disorder: phenotypic and molecular spectrum. J Med Genet. 10.1136/jmg-2022-108803 37185208 10.1136/jmg-2022-108803 PMC10579487

[115] Hong EH, Kim JY, Kim JH, Lim DS, Kim M, Kim JY (2018) BIG2-ARF1-RhoA-mDia1 signaling regulates dendritic golgi polarization in hippocampal neurons. Mol Neurobiol 55:7701–7716. 10.1007/s12035-018-0954-7 29455446 10.1007/s12035-018-0954-7

[116] Romero DM, Zaidi D, Cifuentes-Diaz C, Maillard C, Grannec G, Selloum M, Birling MC, Bahi-Buisson N, Francis F (2023) A human dynein heavy chain mutation impacts cortical progenitor cells causing developmental defects, reduced brain size and altered brain architecture. Neurobiol Dis 180:106085. 10.1016/j.nbd.2023.106085 36933672 10.1016/j.nbd.2023.106085

[117] Jayaraman D, Bae BI, Walsh CA (2018) The genetics of primary microcephaly. Annu Rev Genomics Hum Genet 19:177–200. 10.1146/annurev-genom-083117-021441 29799801 10.1146/annurev-genom-083117-021441

