# Correction: IER3IP1-mutations cause microcephaly by selective inhibition of ER-Golgi transport

**DOI:** 10.1007/s00018-024-05508-5

**Published:** 2024-11-27

**Authors:** Mihaela Anitei, Francesca Bruno, Christina Valkova, Therese Dau, Emilio Cirri, Iván Mestres, Federico Calegari, Christoph Kaether

**Affiliations:** 1 Leibniz Institute on Aging, Fritz-Lipmann-Institute, Beutenbergstr 11, 07745 Jena, Germany; 2 Center for Regenerative Therapies, TU-Dresden, Fetscherstraße 105, 01307 Dresden, Germany

**Cellular and Molecular Life Sciences (2024) 81:334**

10.1007/s00018-024-05386-x

In this article the affiliation details for Author Federico Calegari were incorrectly given as

‘Leibniz Institute on Aging, Fritz-Lipmann-Institute, Beutenbergstr 11, 07745, Jena, Germany’

but should have been

“Center for Regenerative Therapies, TU-Dresden, Fetscherstraße 105, 01307, Dresden, Germany”

and author name Christoph Kaether were incorrectly given as:

“Center for Regenerative Therapies, TU-Dresden, Fetscherstraße 105, 01307, Dresden, Germany”

but should have been

Leibniz Institute on Aging, Fritz-Lipmann-Institute, Beutenbergstr 11, 07745, Jena, Germany,

The original article has updated.

