# Supplementary material for: IER3IP1-mutations cause microcephaly by selective inhibition of ER-Golgi transport
Source: Cell Mol Life Sci. 2024 Aug 8;81(1):334. doi: 10.1007/s00018-024-05386-x (PMC11335259; doi:10.1007/s00018-024-05386-x)

IER3IP1-mutations cause microcephaly by selective inhibition of ER-Golgi transport

Cellular and Molecular Life Sciences

Mihaela Anitei, Francesca Bruno[#] Christina Valkova, Therese Dau, Emilio Cirri, Iván Mestres Lascano, Federico Calegari and Christoph Kaether

Leibniz Institute on Aging, Fritz-Lipmann-Institute, Beutenbergstr. 11, 07745 Jena, Germany, christoph.kaether@leibniz-fli.de

Supplementary Figures and Tables

Supplementary Fig. S1. Characterization of CRISPR/Cas9-generated *IER3IP1* KO and mutant re-expressing HeLa cell lines. a) Two guide RNAs (gRNA_1, gRNA_5) and the primers used to amplify the gRNA_1 cutting site for further characterization are shown. Models indicate b, c) heterozygous indels in the KO2 clone, the large deletion in the KO1 clone, and d, e) the localization of the "Deletion" and "Non-deletion" primers (red = base pairs targeted by gRNA_1). f) Incucyte growth curve analyses of the indicated cell lines (mean±SD, n = 4 independent experiments). g) Control and p.T79Δ mutant-re-expressing cells were treated with 0.66 μM MG132, 33.33 μg/ml chloroquine (CQ) or control DMSO for 24 h, then lysed and analyzed by WB with the indicated antibodies (n = 3 independent experiments). h) mRNA was isolated, and cDNAs were synthesized and analyzed by PCR. i) Control HeLa cells were transfected with mRFP-KDEL, fixed, and labeled with anti-IER3IP1 (green) and Hoechst (blue) (n = 3 independent experiments). Arrows indicate ER membranes co-labeled by IER3IP1. j) Control HeLa cells were co-transfected with mCherry-Sec24C and EGFP-ERGIC53, fixed and stained with anti-IER3IP1 (blue), and analyzed by fluorescence microscopy. Arrows indicate membranes co-labeled by the three proteins. k) Model of IER3IP1 localization relative of ER exit sites, as observed in (j). i, j) Scale bars, 10 μm.

Supplementary Fig. S2. *IER3IP1* KO, *IER3IP1* p.L78P and p.T79Δ mutants delay Golgi assembly. a-c) Cells were treated with DMSO or 5 μg/ml BFA for 1 h, then either fixed, or washed in fresh medium for 2 h following BFA treatment and fixed.

Cells were then stained with anti-ST6GAL1 (green), anti-GM130 (red) and Hoechst (blue) and analyzed by fluorescence microscopy. Scale bars, 10 μm. d) The fraction of cells in which the Golgi apparatus re-assembled after the 2 h wash is shown. Each small circle represents a different image and filled circles the mean values of independent experiments, shown in different colors (n = 3 independent experiments, $n_{Control}$ = 593, n_{KO1} = 1004, n_{KO1+WT} = 906, $n_{KO1+p.L78P}$ = 2390, $n_{KO1+p.V21G}$ = 744, $n_{KO1+p.T79\Delta}$ = 1036 cells). e) Co-localization between GM130 and ST6GAL1 was evaluated by measuring the object-based Pearson's correlation coefficient using Cell Profiler. Data are shown for each object (small circles), and mean values for independent experiments (color-coded filled circles) (n = 3 independent experiments, $n_{Control}$ = 845 cells, 6211 objects, n_{KO1} = 682 cells, 9981 objects, n_{KO1+WT} = 682 cells, 4362 objects, $n_{KO1+p.L78P}$ = 888 cells, 13896 objects, $n_{KO1+p.V21G}$ = 485 cells, 4735 objects, $n_{KO1+p.T79\Delta}$ = 535 cells, 11127 objects). d, e) mean $\pm$ SD, One-way Anova with Dunnett's post-hoc test.

Supplementary Fig. S3. Analyses of the *IER3IP1* p.L78P cell secretome, and of KDELR1_EGFP and KDELR3-EGFP localization. a) Volcano plot of proteins secreted by cell lines expressing *IER3IP1* p.L78P or WT (Suppl. Table S2). Proteins whose secretion was modified in p.L78P *vs.* WT cells are shown in red (increased), or blue (decreased). Gene names are shown for some of the proteins mentioned in the text *(*n_{WT} = 3, $n_{p.L78P}$ = 4). b-e) GSEA pathway enrichment analysis (reactome, GO cellular component/GOCC) of proteins whose secretion was either decreased (red) or increased (blue) in *IER3IP1* p.L78P- compared to *IER3IP1* WT expressing cells. f-i) Localization of KDELR1-EGFP and KDELR3-EGFP. Cells were transfected with (f) KDELR1-EGFP or (g) KDELR3-EGFP, fixed, co-labeled with anti-GM130 (red) and Hoechst (blue) and analyzed by fluorescence microscopy. Scale bars, 10 μm. h, i) Ratios of KDELR1-EGFP (h) or KDELR3-EGFP (i) fluorescence in the Golgi region and the entire cell were calculated using Fiji. Color-coded mean values of independent experiments (large, filled circles,) and values calculated for individual cells (small circles) are shown ($n_{KDELR1-EGFP}$ = 4 and $n_{KDELR1-EGFP}$ = 6 independent experiments; mean $\pm$ SD, One-way Anova with Dunnett's post-hoc test; KDELR1-EGFP: $n_{Control}$ =

216, n $_{KO1}$ = 238, n $_{KO2}$ = 166, n $_{KO1+WT}$ = 135, n $_{KO1+p.L78P}$ = 156 cells; KDELR3-EGFP: n $_{Control}$ = 434, n $_{KO1}$ = 575, n $_{KO2}$ = 289, n $_{KO1+WT}$ = 172, n $_{KO1+p.L78P}$ = 294 cells).

Supplementary Fig. S4. Additional data of surface and total proteome analysis. a, b) Quantification of total protein levels of endogenous FGFR3 (a) and Unc5B-FLAG (b), as shown in Fig. 4h and Fig. 4j, respectively (n = 6 independent experiments; median and IQR values, Kruskal-Wallis with Dunn's post-hoc test). c, d) Analysis of Unc5B-FLAG glycosylation. c) Indicated cell lines were transfected with Unc5B-FLAG, lysates were collected after 24 h, treated with EndoH or PNGaseF enzymes overnight, and immunoblotted with anti-FLAG and anti-beta-actin as loading control. * shows the mature, upper band of Unc5B-FLAG that is resistant to EndoH but not to PNGAseF, ** shows the immature, lower band, detected at a lower molecular weight following EndoH deglycosylation. d) Quantification of mature/total Unc5B-FLAG (n = 3 independent experiments, mean ± SD, One-way Anova with Dunnett's post-hoc test).e) Volcano plot of proteins identified in the whole cell lysates of control and *IER3IP1* KO1 HeLa cells (see Suppl. Table S3). Proteins significantly different in KO1 *versus* control cells are color-coded as shown in the legend: early/late endosome (red) and cell growth (yellow), transport vesicle (blue) and cell junction (green)-associated. Large circles indicate modifications rescued by the re-expression of the WT protein (n = 4 independent experiments). f-i) Pathway enrichment analysis of hits (f, g) decreased (red) or (h, i) increased (blue) in the cell lysates of *IER3IP1* KO1 compared to control cells. j, k) Quality control of MS analyses; mean ± SD of Pearson correlations of log2 PG.Quantities measured for the surface proteome (f) and secretome (g).

Supplementary Fig. S5. *IER3IP1* KO1 and re-expressed *IER3IP1* p.L78P increase the number of active lysosomes. a-d) Cells were (a) incubated with Magic Red substrate MR-FR_2 and Hoechst 33342 dye for 60 min and directly imaged, or (b) fixed and labeled with anti-LAMP1 and Hoechst 33342, then analyzed by high-content confocal fluorescence microscopy. Max projections of Z-stacks are shown for (b). Scale bars, 10 μm. c, d) The number of objects per image was calculated using Cell Profiler and normalized to the number of nuclei. Color-coded small circles represent

mean values per well, and filled, large circles depict the means of each independent experiment (n = 4, mean ± SD; One-way Anova with Dunnett's post-hoc test. c) $n_{Control}$ = 34 363 cells, 19 images; n_{KO1} = 29 720 cells, 20 images; n_{KO1+WT} = 31 535 cells, 20 images; $n_{KO1+p.L78P}$ = 28 188 cells, 19 images; d) $n_{Control}$ = 37 847 cells, 20 images; n_{KO1} = 39 965 cells, 23 images; n_{KO1+WT} = 39 277 cells, 23 images; $n_{KO1+p.L78P}$ = 49 706 cells, 23 images. e-g) Cells were treated with 500 nM Torin 1 and EBSS starvation medium, or 50 μg/ml chloroquine (CQ) for 5h, then lysed and analyzed by immunoblotting with anti-p62, anti-LC3B and anti-beta-actin as loading control. f, g) The protein amounts of LC3II (f) and p62 (g) normalized to beta-actin are shown as fold change over the respective non-treated cell line. (f) n = 3, (g) n = 4, (f, g) mean ± SD, two-tailed Mann-Whitney test.

Supplementary Fig. S6. Transferrin endocytosis is not changed in the absence of functional IER3IP1. a) The areas of the perinuclear regions used for quantification of cells from Fig. 2a are delineated. b) The indicated HeLa cell lines were starved for 1h, then incubated with Tfn-Alexa 647 for 10 min, fixed and analyzed by fluorescence microscopy. c) The numbers of Tfn objects were counted using Cell Profiler. Measurements from individual cells (small circles), mean values per experiment (large, filled circles) are indicated, with biological replicates color-coded (n = 3 independent experiments, > 126 cells/condition, mean ± SD, Kruskal-Wallis test). Scale bars, 10 μm.

Supplementary Fig. S7. The absence of functional IER3IP1 increased the area of ER sheets but did not induce significant ER-stress. a) Cells were transfected with EGFP-p180 as a marker for ER sheets and analyzed after 24 h using live fluorescence microscopy. Images shown as 3D Max Projections of Z-sections (0.26 μm per Z-section) were analyzed using Fiji. Scale bars, 10 μm. b) The ratios between the area covered by EGFP-p180 and the total cell area are shown. Each circle represents a cell, color-coded mean values for each independent are shown as filled circles (n = 3 independent experiments, mean ± SD, One-way Anova with Dunnett's post-hoc test; $n_{Control}$ = 97, n_{KO1} = 129, n_{KO1+WT} = 104, $n_{KO1+p.L78P}$ = 131 cells). c-f) Cells

overexpressing FGFR5-V5 or control V5 (c, d), or treated with 1 μM thapsigargin or control DMSO for 24 h (e, f) were analyzed by immunoblotting (n = 3 independent experiments, mean ± SD, two-tailed Welch's t-test). g-i) Cells treated with thapsigargin or control DMSO for 4 h were analyzed by immunoblotting with the indicated antibodies. (g) n = 3, (h-i) n = 5 independent experiments, mean ± SD, One-way Anova with Dunnett's post-hoc test, and two-tailed Welch's t-test.

Supplementary Fig. S8. IER3IP1 KO affects the localization of SEMA4D-myc in HeLa cells. a) HeLa cells were transfected with Sema4D-myc for 24 h, then fixed and analyzed by fluorescence microscopy. Arrows indicate areas labeled by either of the two proteins. Scale bars, 10 μm. b) Quantification of the percentage of cells with Sema4D-myc positive whorl-like structures (n = 3, mean± SD, One-way Anova with Dunnett's post-hoc test, n > 87 cells/condition).

Supplementary Fig. S9. Ier3ip1 KD has no major effects on neuronal migration and differentiation in embryonic mouse brains. Embryonic mouse brains were electroporated with shRNAs at E13.5, injected with EdU at E14.5 and collected at E15.5. Coronal sections were fixed and labeled with the indicated antibodies and Hoechst nuclear dye. Where indicated, specific regions (VZ, SVZ, IZ and CP) were distinguished based on nuclear density and morphology, and co-staining with specific markers as indicated. a) shIer3ip1 does not have major effects on neuronal migration. EGFP+ cell distribution between the indicated regions. The area between the VZ and the CP was divided into 4 equal regions (I1-I4). b) Quantification of the percentages of EGFP+ cells in each of the indicated regions (shLuciferase n = 8 brains, 11 sections, 1393 EGFP+ cells; shIer3ip1 n = 6 brains, 8 sections, 1155 EGFP+ cells, mean ± SD, two tailed Welch's t-test). c) shIer3ip1 does not affect neuronal progenitor cycle exit. Sections were labeled with anti-Ki67 (red) and EdU (blue). VZ and SVZ were defined based on Ki67+ and EdU+ cell morphology and density. The area between SVZ and CP (IZ) was divided into 3 equal sections (IZ1-IZ3). d) The percentages of Ki67-EdU+EGFP+ of EdU+EGFP+ cells were calculated (shLuciferase n = 4 brains, 4 sections, 242 EGFP+ cells; shIer3ip1 n = 4 brains, 4 sections, 217 EGFP+ cells,

median ± IQR, two-tailed Mann-Whitney test). e) shIer3ip1 does not change basal progenitor differentiation. Sections were labeled with anti-Tbr2 (red) and Hoechst (blue) and g) the percentages of Tbr+EGFP+ of EGFP+ cells were quantified (shLuciferase n = 3 brains, 4 sections, 135 EGFP+ cells; shIer3ip1 n = 3 brains, 6 sections, 316 EGFP+ cells, median ± IQR, two-tailed Mann-Whitney test). f) Sections were labeled with anti-Cas3 (red) and Hoechst (blue), and i) the percentages of EGFP+ cells additionally positive for Cas3 were calculated (shLuciferase n = 2 brains, 4 sections, 302 EGFP+ cells; shIer3ip1 n = 3 brains, 4 sections, 445 EGFP+ cells, median ± IQR, two-tailed Mann-Whitney test). All images represent maximum projections of sequential Z-sections. Scale bars, 100 µm.

Supplementary Fig. S10. Full size Western blots from Fig. 2f, Fig. 3i, h, and Fig. 5h, i.

Supplementary Tables

Each Table contains a Title page

Supplementary Table S1. Specific enrichment of biotinylated secreted proteins.

Sheet 1. List of secreted proteins enriched in biotinylated vs non-biotinylated control cells. Proteins significantly modified are highlighted in blue, and those unchanged are not highlighted.

Sheet 2. List of N-Glycosylated proteins identified with the keyword “Glycoproteins” (www.uniprot.org) among the enriched biotinylated proteins.

Sheet 3. List of proteins identified among the enriched biotinylated proteins with the keywords “plasma membrane”, “cell surface”, “extracellular space”, “extracellular matrix”, “extracellular exosome (www.uniprot.org) and confirmed using https://compartments.jensenlab.org/Search.

Sheet 4. List of proteins identified among the enriched biotinylated proteins with the keyword “Secreted” (www.uniprot.org).

Sheet 5. List of proteins identified among the enriched biotinylated proteins with the keyword "Transmembrane" (www.uniprot.org).

Sheet 6. List of proteins identified among the enriched biotinylated proteins with the keyword "Signal peptide" (www.uniprot.org).

Supplementary Table S2. MS analysis of differentially secreted proteins in *IER3IP1* KO1 *versus* control cells.

Sheet 1. List of proteins whose secretion is modified in *IER3IP1* KO1 *vs.* control cells (Fig. 4a). Ratios between *IER3IP1* KO1 and control cells are shown (increased, blue; reduced, pink; unchanged, not highlighted).

Sheet 2. List of proteins whose secretion is modified in *IER3IP1* KO1 *vs.* control cells, and rescued by re-expression of *IER3IP1* WT (i.e., expression is not significantly changed in *IER3IP1* WT *vs.* control cells) (Fig. 4a, c). Proteins partially rescued are highlighted in light orange.

Sheet 3. List of proteins whose secretion is modified in *IER3IP1* WT *vs.* control cells. Ratios between *IER3IP1* WT and control cells are shown in blue (increased) pink (decreased) or were not highlighted (unchanged).

Sheet 4. List of proteins whose secretion is modified in *IER3IP1* p.L78P *vs.* WT cells (Suppl. Fig. S5a). Proteins are highlighted in blue (increased), pink (decreased) or not highlighted (unchanged). Proteins that are differentially secreted in *IER3IP1* KO1/Control and *IER3IP1* p.L78P/*IER3IP1* WT are shown in bold.

Sheet 5. GSEA Reactome analysis of proteins whose secretion is decreased in *IER3IP1* KO1 *vs.* control cells (Fig. 4a, d).

Sheet 6. GSEA GO Cell Component analysis of proteins whose secretion is decreased in *IER3IP1* KO1 *vs.* control cells (Fig. 4a, e).

Sheet 7. GSEA Reactome analysis of proteins whose secretion is increased in *IER3IP1* KO1 *vs.* control cells (Fig. 4a, f).

Sheet 8. GSEA GO Cell Component analysis of proteins whose secretion is increased in *IER3IP1* KO1 *vs.* control cells (Fig. 4a, g).

Sheet 9. GSEA Reactome analysis of proteins whose secretion is decreased in *IER3IP1* p.L78P *vs.* WT cells (Suppl. Fig. S5a, b).

Sheet 10. GSEA GO Cell Component analysis of proteins whose secretion is decreased in *IER3IP1* p.L78P *vs.* WT cells (Suppl. Fig. S5a, c).

Sheet 11. GSEA Reactome analysis of proteins whose secretion is increased in *IER3IP1* p.L78P *vs.* WT (Suppl. Fig. S5a, d).

Sheet 12. GSEA GO Cell Component analysis of proteins whose secretion is increased in *IER3IP1* p.L78P *vs.* WT cells (Suppl. Fig. S5a, e).

Supplementary Table S3. Total proteome analysis in *IER3IP1* KO1 *versus* control cells.

Sheet 1. List of proteins whose total cellular expression is modified in *IER3IP1* KO1 *vs.* control cells (Suppl. Fig. S1j). Ratios between *IER3IP1* KO1 and control cells are shown (increased, blue; reduced, pink; unchanged, not highlighted).

Sheet 2. GSEA GO Cell Component analysis of proteins whose total levels are decreased in *IER3IP1* KO1 *vs.* control cells (Suppl. Fig. S1j, k).

Sheet 3. GSEA GO Biological Process analysis of proteins whose total levels are decreased in *IER3IP1* KO1 *vs.* control cells (Suppl. Fig. S1j, l).

Sheet 4. GSEA GO Cell Component analysis of proteins whose total levels are increased in *IER3IP1* KO1 *vs.* control cells (Suppl. Fig. S1j, m).

Sheet 5. GSEA Reactome analysis of proteins whose total levels are increased in *IER3IP1* KO1 *vs.* control cells (Suppl. Fig. S1j, n).

Supplementary Table S4. ER retention motifs. List of proteins identified with the motifs “Prevents secretion from ER” or “Non-canonical ER retention motif” (

www.uniprot.org) among the proteins whose secretion is higher in *IER3IP1* KO1 cells compared to control.

Supplementary Table S5. Specific enrichment of biotinylated surface proteins among surface proteins.

Sheet 1. List of surface proteins enriched in biotinylated vs non-biotinylated control cells (highlighted in grey). Proteins unchanged between the two conditions not highlighted.

Sheet 2. List of surface proteins enriched in biotinylated control cells identified with the keyword "Glycoproteins" (www.uniprot.org).

Sheet 3. List of surface proteins enriched in biotinylated control cells identified with the keyword "Transmembrane" (www.uniprot.org).

Sheet 4. List of surface proteins enriched in biotinylated control cells identified with the keywords "plasma membrane", "cell surface", "extracellular space", "extracellular matrix", "extracellular exosome (www.uniprot.org) and confirmed using https://compartments.jensenlab.org/Search.

Sheet 5. List of surface proteins enriched in biotinylated control cells identified with the keyword "Signal peptide" (www.uniprot.org).

Supplementary Table S6. MS analysis of proteins differentially expressed on cell surface in *IER3IP1* KO1 *versus* control cells.

Sheet 1. List of proteins whose surface expression is modified in *IER3IP1* KO1 *vs.* control cells (volcano plot shown in Fig. 3a). Proteins whose surface levels are increased (blue), reduced (pink) or are not changed (no highlighting) between the two conditions are shown.

Sheet 2. List of proteins whose surface expression is modified in *IER3IP1* KO1 *vs.* control cells, rescued by re-expression of *IER3IP1* WT (i.e., expression is not significantly changed in *IER3IP1* WT *vs.* control cells) (Fig. 3a, c).

Sheet 3. List of proteins whose surface expression is modified in *IER3IP1* WT *vs.* control cells. Ratios between *IER3IP1* WT and control cells are shown (increased, blue; reduced, pink; unchanged, no highlighting).

Sheet 4. GSEA GO Cell Component analysis of proteins whose surface levels are decreased in *IER3IP1* KO1 *vs.* control cells (Fig. 3d).

Sheet 5. GSEA GO Cell Component analysis of proteins whose surface levels are increased in *IER3IP1* KO1 *vs.* control cells (Fig. 3f).

Sheet 6. GSEA Reactome analysis of proteins whose surface levels are decreased in *IER3IP1* KO1 *vs.* control cells (Fig. 3e).

Sheet 7. GSEA Reactome analysis of proteins whose surface levels are increased in *IER3IP1* KO1 *vs.* control cells (Fig. 3g).

Supplementary Table S7. Baso-lateral and apical proteins whose surface expression or secretion was modified in *IER3IP1* KO1 cells compared to controls. Polarized protein distribution was analyzed with http://polarprotdb.ttk.hu/search [1].

Supplementary Table S8. MS analysis of differentially secreted proteins in IER3IP1 KO versus control i3N neurons.

Sheet 1 List of proteins whose secretion is modified in *IER3IP1* KO1 *vs.* control i3N cells (Fig. 6d, f). Ratios between *IER3IP1* KO and control cells are shown.

Sheet 2 GSEA Reactome analysis of proteins whose secretion is decreased in *IER3IP1* KO *vs.* control cells (Fig. 6e).

Sheet 3 Uniprot ID mapping of of proteins whose secretion is decreased in *IER3IP1* KO *vs.* control cells.

1. Zeke, A., et al., *PolarProtDb: A Database of Transmembrane and Secreted Proteins showing Apical-Basal Polarity.* J Mol Biol, 2021. **433**(11): p. 166705.

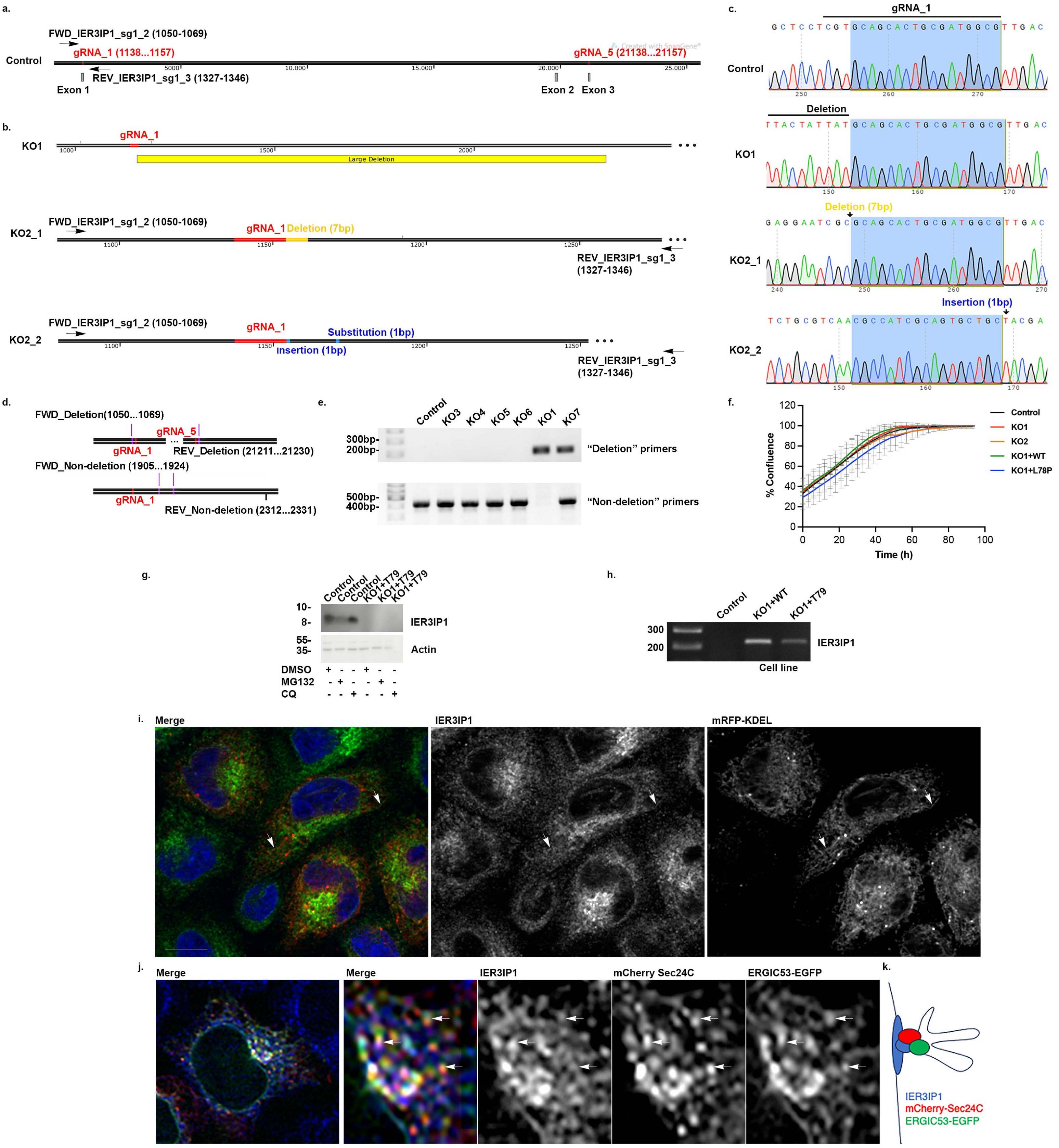

a.
FWD_IER3IP1_sg1_2 (1050-1069)
gRNA_1 (1138...1157)
gRNA_5 (21138...21157)
Control
5000
10.000
15.000
20.000
25.000
REV_IER3IP1_sg1_3 (1327-1346)
Exon 1
Exon 2
Exon 3
b.
KO1
gRNA_1
1000
1500
2000
Large Deletion
KO2_1
FWD_IER3IP1_sg1_2 (1050-1069)
gRNA_1
Deletion (7bp)
1100
1150
1200
1250
REV_IER3IP1_sg1_3 (1327-1346)
KO2_2
FWD_IER3IP1_sg1_2 (1050-1069)
gRNA_1
Substitution (1bp)
Insertion (1bp)
REV_IER3IP1_sg1_3 (1327-1346)
c.
gRNA_1
Control
Deletion
KO1
Deletion (7bp)
KO2_1
Insertion (1bp)
KO2_2
d.
FWD_Deletion(1050...1069)
gRNA_5
gRNA_1
REV_Deletion (21211...21230)
FWD_Non-deletion (1905...1924)
gRNA_1
REV_Non-deletion (2312...2331)
e.
Control
KO3
KO4
KO5
KO6
KO1
KO7
300bp-
200bp-
"Deletion" primers
500bp-
400bp-
"Non-deletion" primers
f.
% Confluence
Time (h)
Control
KO1
KO2
KO1+WT
KO1+L78P
g.
Control
Control
Control
KO1+T79
KO1+T79
KO1+T79
10-
8-
IER3IP1
55-
35-
Actin
DMSO
MG132
CQ
h.
Control
KO1+WT
KO1+T79
300
200
IER3IP1
Cell line
i.
Merge
IER3IP1
mRFP-KDEL
j.
Merge
Merge
IER3IP1
mCherry Sec24C
ERGIC53-EGFP
k.
IER3IP1
mCherry-Sec24C
ERGIC53-EGFP

Suppl. Fig. S1

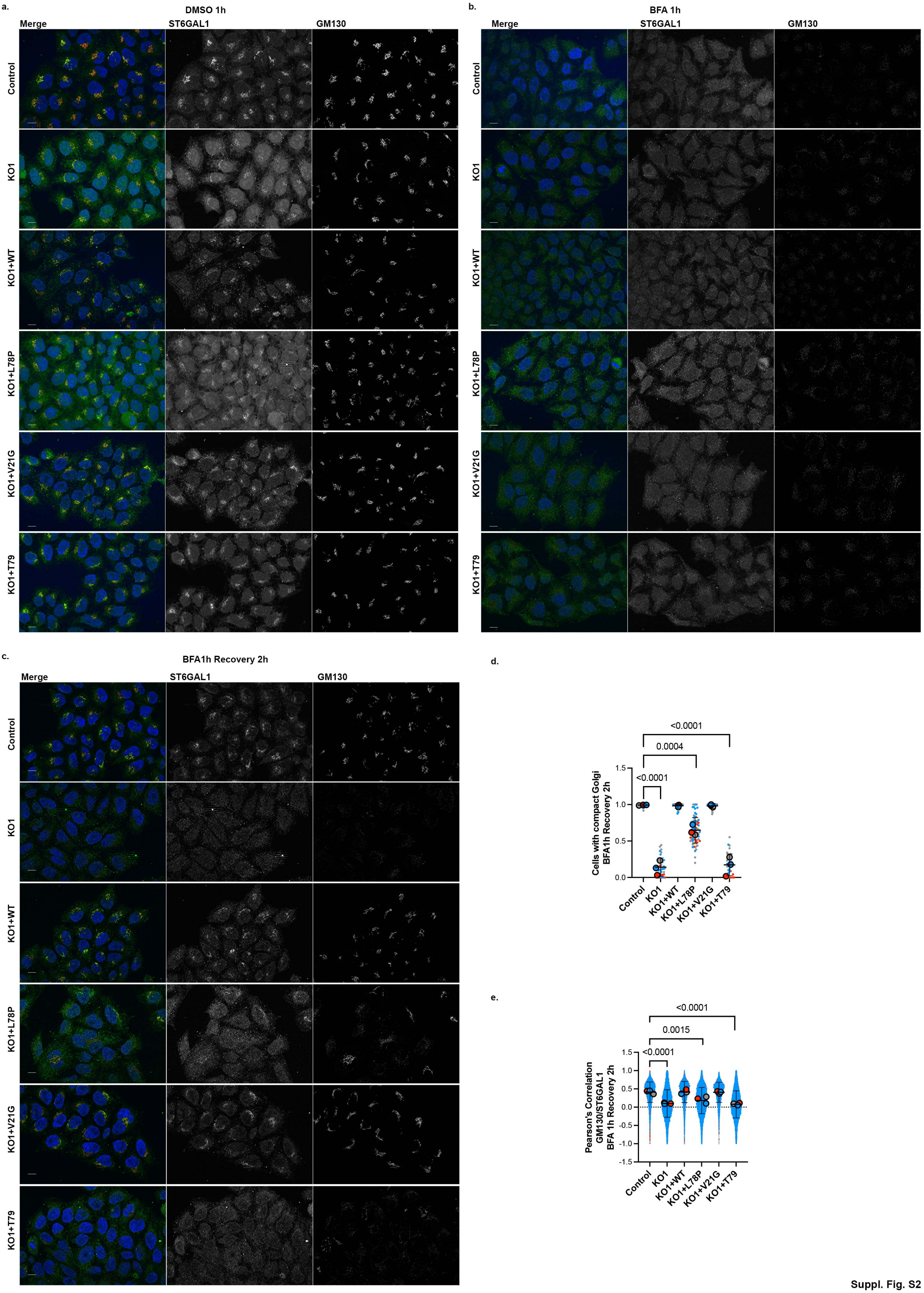
a.
DMSO 1h
Merge
ST6GAL1
GM130
Control
KO1
KO1+WT
KO1+L78P
KO1+V21G
KO1+T79
b.
BFA 1h
Merge
ST6GAL1
GM130
Control
KO1
KO1+WT
KO1+L78P
KO1+V21G
KO1+T79
c.
BFA1h Recovery 2h
Merge
ST6GAL1
GM130
Control
KO1
KO1+WT
KO1+L78P
KO1+V21G
KO1+T79
d.
Cells with compact Golgi
BFA1h Recovery 2h
<0.0001
0.0004
<0.0001
1.5
1.0
0.5
0.0
Control
KO1
KO1+WT
KO1+L78P
KO1+V21G
KO1+T79
e.
Pearson's Correlation
GM130/ST6GAL1
BFA 1h Recovery 2h
<0.0001
0.0015
<0.0001
1.5
1.0
0.5
0.0
-0.5
-1.0
-1.5
Control
KO1
KO1+WT
KO1+L78P
KO1+V21G
KO1+T79

Suppl. Fig. S2

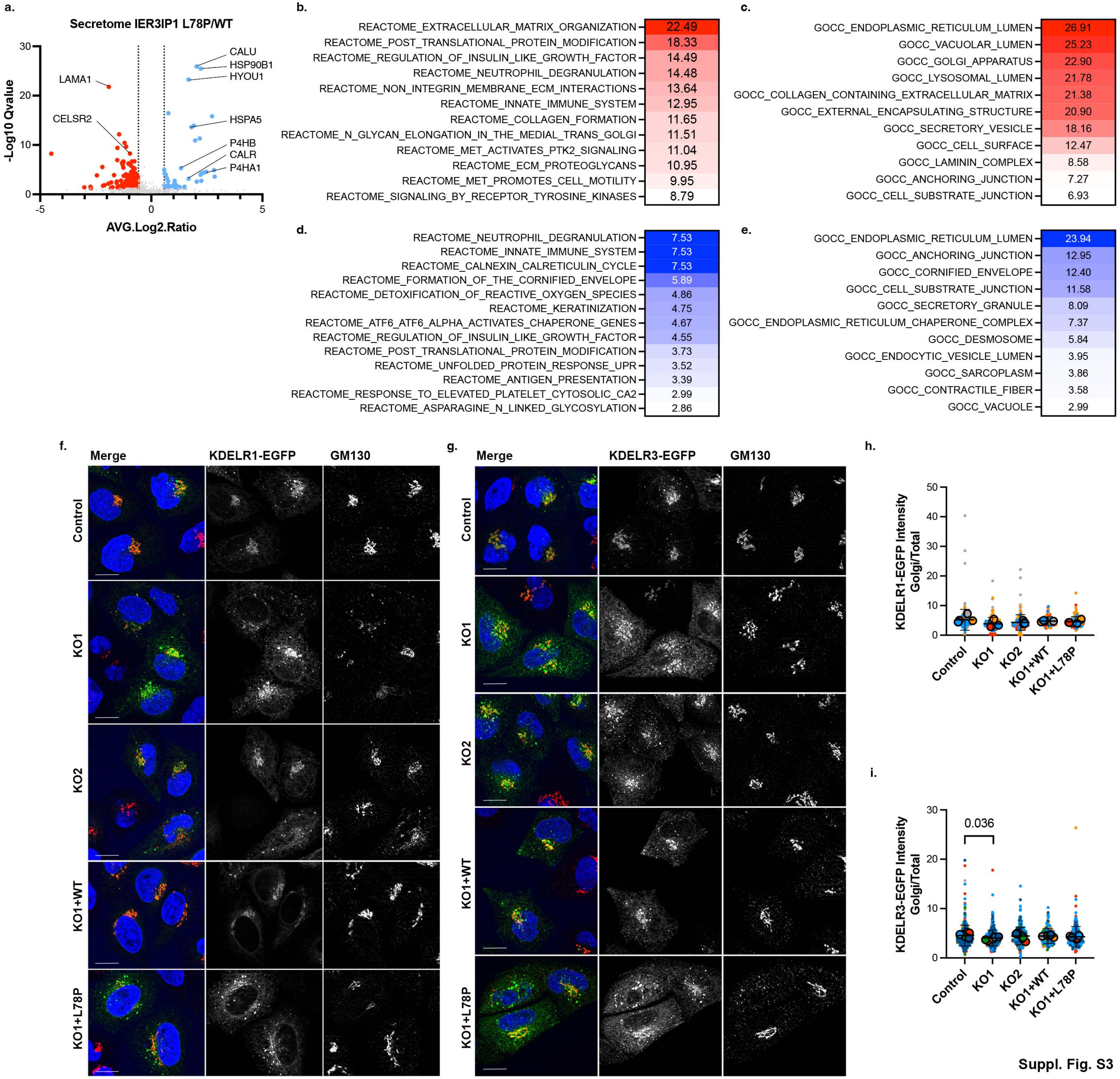
a.
Secretome IER3IP1 L78P/WT
-Log10 Qvalue
AVG.Log2.Ratio
LAMA1
CELSR2
CALU
HSP90B1
HYOU1
HSPA5
P4HB
CALR
P4HA1
b.
REACTOME_EXTRACELLULAR_MATRIX_ORGANIZATION 22.49
REACTOME_POST_TRANSLATIONAL_PROTEIN_MODIFICATION 18.33
REACTOME_REGULATION_OF_INSULIN_LIKE_GROWTH_FACTOR 14.49
REACTOME_NEUTROPHIL_DEGRANULATION 14.48
REACTOME_NON_INTEGRIN_MEMBRANE_ECM_INTERACTIONS 13.64
REACTOME_INNATE_IMMUNE_SYSTEM 12.95
REACTOME_COLLAGEN_FORMATION 11.65
REACTOME_N_GLYCAN_ELONGATION_IN_THE_MEDIAL_TRANS_GOLGI 11.51
REACTOME_MET_ACTIVATES_PTK2_SIGNALING 11.04
REACTOME_ECM_PROTEOGLYCANS 10.95
REACTOME_MET_PROMOTES_CELL_MOTILITY 9.95
REACTOME_SIGNALING_BY_RECEPTOR_TYROSINE_KINASES 8.79
c.
GOCC_ENDOPLASMIC_RETICULUM_LUMEN 26.91
GOCC_VACUOLAR_LUMEN 25.23
GOCC_GOLGI_APPARATUS 22.90
GOCC_LYSOSOMAL_LUMEN 21.78
GOCC_COLLAGEN_CONTAINING_EXTRACELLULAR_MATRIX 21.38
GOCC_EXTERNAL_ENCAPSULATING_STRUCTURE 20.90
GOCC_SECRETORY_VESICLE 18.16
GOCC_CELL_SURFACE 12.47
GOCC_LAMININ_COMPLEX 8.58
GOCC_ANCHORING_JUNCTION 7.27
GOCC_CELL_SUBSTRATE_JUNCTION 6.93
d.
REACTOME_NEUTROPHIL_DEGRANULATION 7.53
REACTOME_INNATE_IMMUNE_SYSTEM 7.53
REACTOME_CALNEXIN_CALRETICULIN_CYCLE 7.53
REACTOME_FORMATION_OF_THE_CORNIFIED_ENVELOPE 5.89
REACTOME_DETOXIFICATION_OF_REACTIVE_OXYGEN_SPECIES 4.86
REACTOME_KERATINIZATION 4.75
REACTOME_ATF6_ATF6_ALPHA_ACTIVATES_CHAPERONE_GENES 4.67
REACTOME_REGULATION_OF_INSULIN_LIKE_GROWTH_FACTOR 4.55
REACTOME_POST_TRANSLATIONAL_PROTEIN_MODIFICATION 3.73
REACTOME_UNFOLDED_PROTEIN_RESPONSE_UPR 3.52
REACTOME_ANTIGEN_PRESENTATION 3.39
REACTOME_RESPONSE_TO_ELEVATED_PLATELET_CYTOSOLIC_CA2 2.99
REACTOME_ASPARAGINE_N_LINKED_GLYCOSYLATION 2.86
e.
GOCC_ENDOPLASMIC_RETICULUM_LUMEN 23.94
GOCC_ANCHORING_JUNCTION 12.95
GOCC_CORNIFIED_ENVELOPE 12.40
GOCC_CELL_SUBSTRATE_JUNCTION 11.58
GOCC_SECRETORY_GRANULE 8.09
GOCC_ENDOPLASMIC_RETICULUM_CHAPERONE_COMPLEX 7.37
GOCC_DESMOSOME 5.84
GOCC_ENDOCYTIC_VESICLE_LUMEN 3.95
GOCC_SARCOPLASM 3.86
GOCC_CONTRACTILE_FIBER 3.58
GOCC_VACUOLE 2.99
f.
Merge
KDELR1-EGFP
GM130
Control
KO1
KO2
KO1+WT
KO1+L78P
g.
Merge
KDELR3-EGFP
GM130
h.
KDELR1-EGFP Intensity Golgi/Total
i.
KDELR3-EGFP Intensity Golgi/Total
0.036

Suppl. Fig. S3

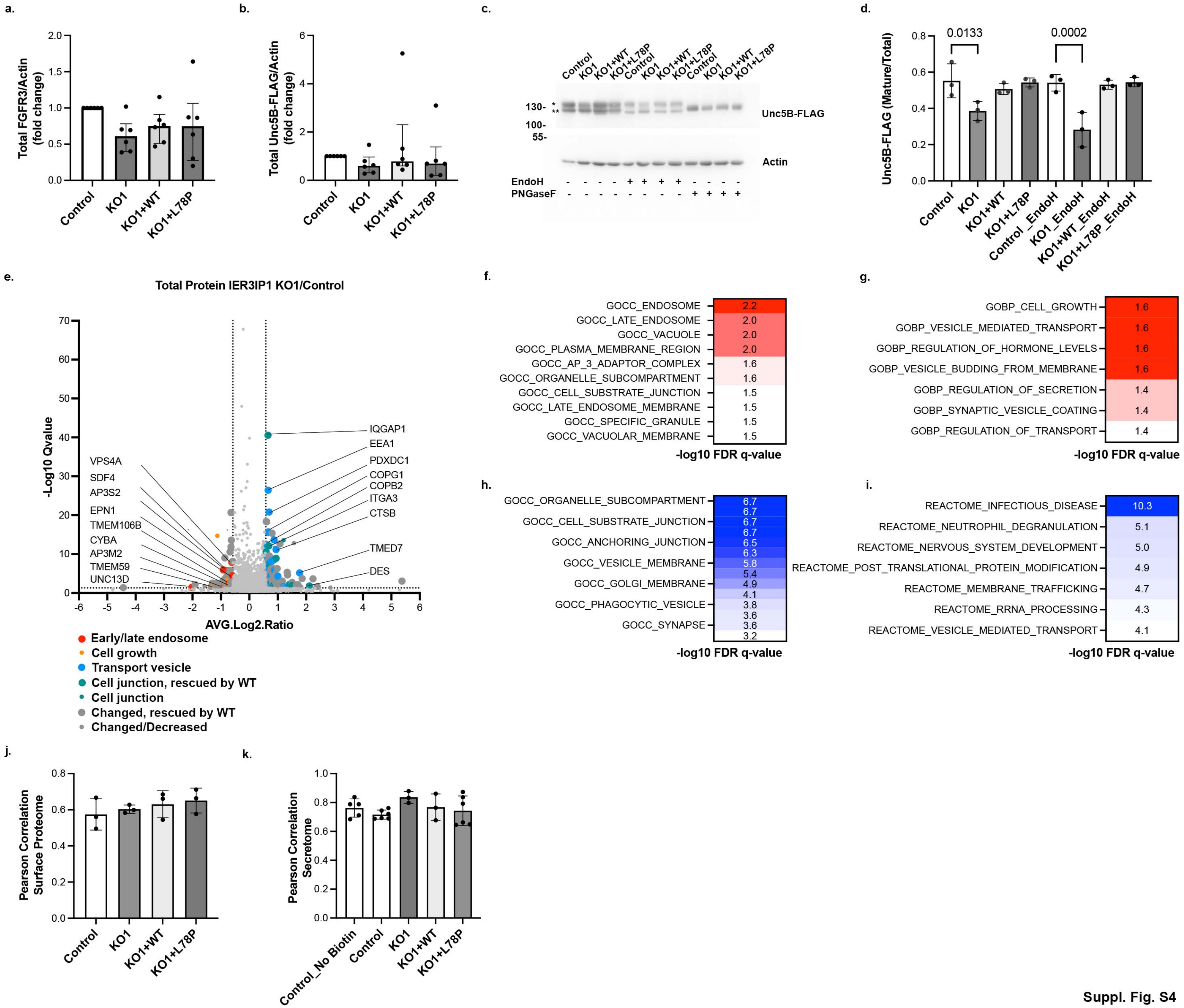

a.
Total FGFR3/Actin (fold change)
Control
KO1
KO1+WT
KO1+L78P
b.
Total Unc5B-FLAG/Actin (fold change)
c.
Control KO1 KO1+WT KO1+L78P Control KO1 KO1+WT KO1+L78P Control KO1 KO1+WT KO1+L78P
130-
100-
55-
*
**
Unc5B-FLAG
Actin
EndoH - - - - + + + + - - - -
PNGaseF - - - - - - - - + + + +
d.
Unc5B-FLAG (Mature/Total)
0.0133
0.0002
Control_EndoH
KO1_EndoH
KO1+WT_EndoH
KO1+L78P_EndoH
e.
Total Protein IER3IP1 KO1/Control
-Log10 Qvalue
AVG.Log2.Ratio
VPS4A
SDF4
AP3S2
EPN1
TMEM106B
CYBA
AP3M2
TMEM59
UNC13D
IQGAP1
EEA1
PDXDC1
COPG1
COPB2
ITGA3
CTSB
TMED7
DES
Early/late endosome
Cell growth
Transport vesicle
Cell junction, rescued by WT
Cell junction
Changed, rescued by WT
Changed/Decreased
f.
GOCC_ENDOSOME 2.2
GOCC_LATE_ENDOSOME 2.0
GOCC_VACUOLE 2.0
GOCC_PLASMA_MEMBRANE_REGION 2.0
GOCC_AP_3_ADAPTOR_COMPLEX 1.6
GOCC_ORGANELLE_SUBCOMPARTMENT 1.6
GOCC_CELL_SUBSTRATE_JUNCTION 1.5
GOCC_LATE_ENDOSOME_MEMBRANE 1.5
GOCC_SPECIFIC_GRANULE 1.5
GOCC_VACUOLAR_MEMBRANE 1.5
-log10 FDR q-value
g.
GOBP_CELL_GROWTH 1.6
GOBP_VESICLE_MEDIATED_TRANSPORT 1.6
GOBP_REGULATION_OF_HORMONE_LEVELS 1.6
GOBP_VESICLE_BUDDING_FROM_MEMBRANE 1.6
GOBP_REGULATION_OF_SECRETION 1.4
GOBP_SYNAPTIC_VESICLE_COATING 1.4
GOBP_REGULATION_OF_TRANSPORT 1.4
-log10 FDR q-value
h.
GOCC_ORGANELLE_SUBCOMPARTMENT
GOCC_CELL_SUBSTRATE_JUNCTION
GOCC_ANCHORING_JUNCTION
GOCC_VESICLE_MEMBRANE
GOCC_GOLGI_MEMBRANE
GOCC_PHAGOCYTIC_VESICLE
GOCC_SYNAPSE
6.7 6.7 6.7 6.7 6.5 6.3 5.8 5.4 4.9 4.1 3.8 3.6 3.6 3.2
-log10 FDR q-value
i.
REACTOME_INFECTIOUS_DISEASE 10.3
REACTOME_NEUTROPHIL_DEGRANULATION 5.1
REACTOME_NERVOUS_SYSTEM_DEVELOPMENT 5.0
REACTOME_POST_TRANSLATIONAL_PROTEIN_MODIFICATION 4.9
REACTOME_MEMBRANE_TRAFFICKING 4.7
REACTOME_RRNA_PROCESSING 4.3
REACTOME_VESICLE_MEDIATED_TRANSPORT 4.1
-log10 FDR q-value
j.
Pearson Correlation Surface Proteome
k.
Pearson Correlation Secretome
Control_No Biotin

Suppl. Fig. S4

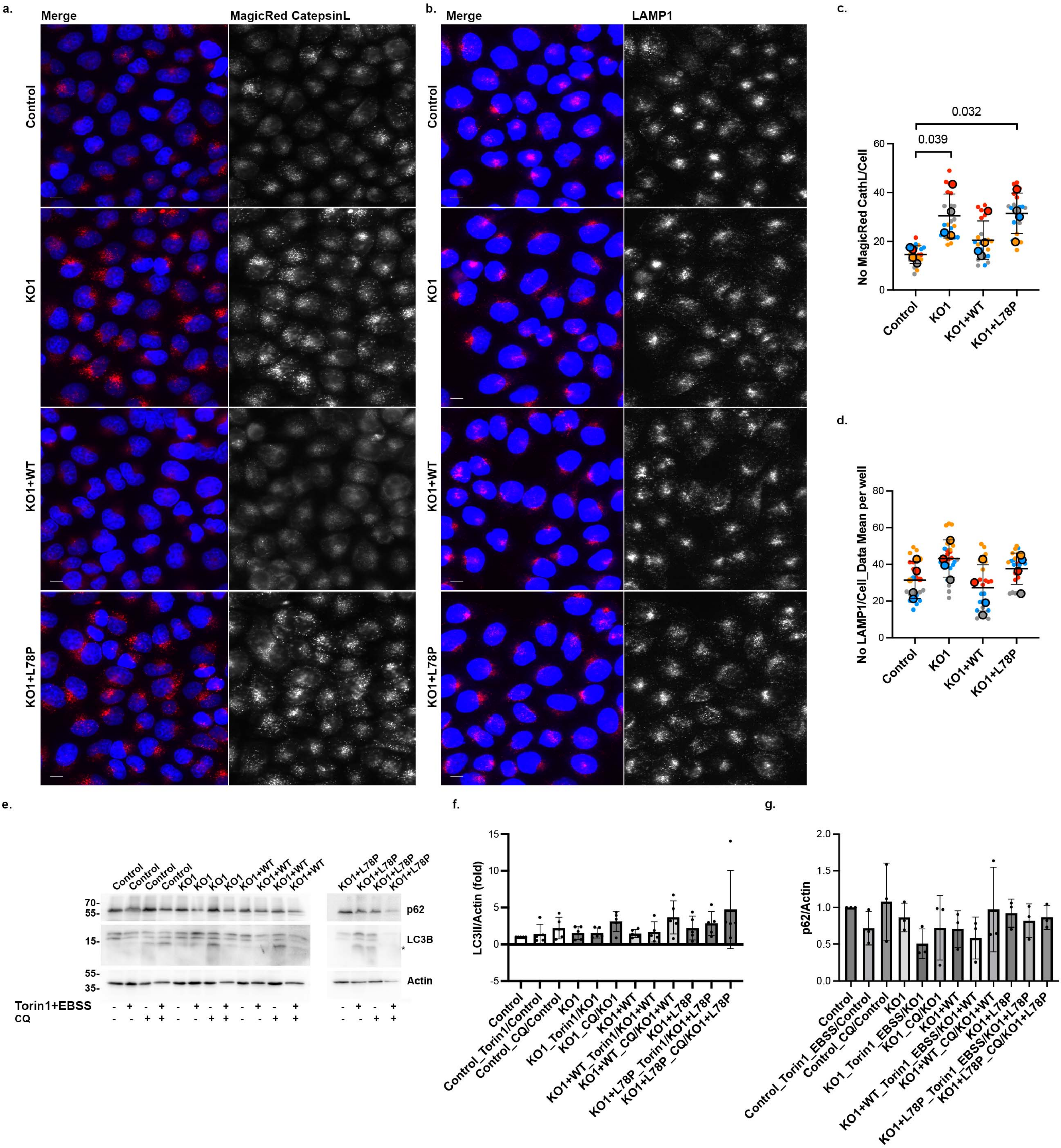
a.
Merge
MagicRed CatepsinL
Control
KO1
KO1+WT
KO1+L78P
b.
Merge
LAMP1
c.
0.032
0.039
No MagicRed CathL/Cell
Control
KO1
KO1+WT
KO1+L78P
d.
No LAMP1/Cell_Data Mean per well
e.
Control
KO1
KO1+WT
KO1+L78P
p62
LC3B
Actin
Torin1+EBSS
CQ
f.
LC3II/Actin (fold)
Control_Torin1/Control
Control_CQ/Control
KO1_Torin1/KO1
KO1_CQ/KO1
KO1+WT_Torin1/KO1+WT
KO1+WT_CQ/KO1+WT
KO1+L78P_Torin1/KO1+L78P
KO1+L78P_CQ/KO1+L78P
g.
p62/Actin
Control_Torin1_EBSS/Control
Control_CQ/Control
KO1_Torin1_EBSS/KO1
KO1_CQ/KO1
KO1+WT_Torin1_EBSS/KO1+WT
KO1+WT_CQ/KO1+WT
KO1+L78P_Torin1_EBSS/KO1+L78P
KO1+L78P_CQ/KO1+L78P

Suppl . Fig. S5

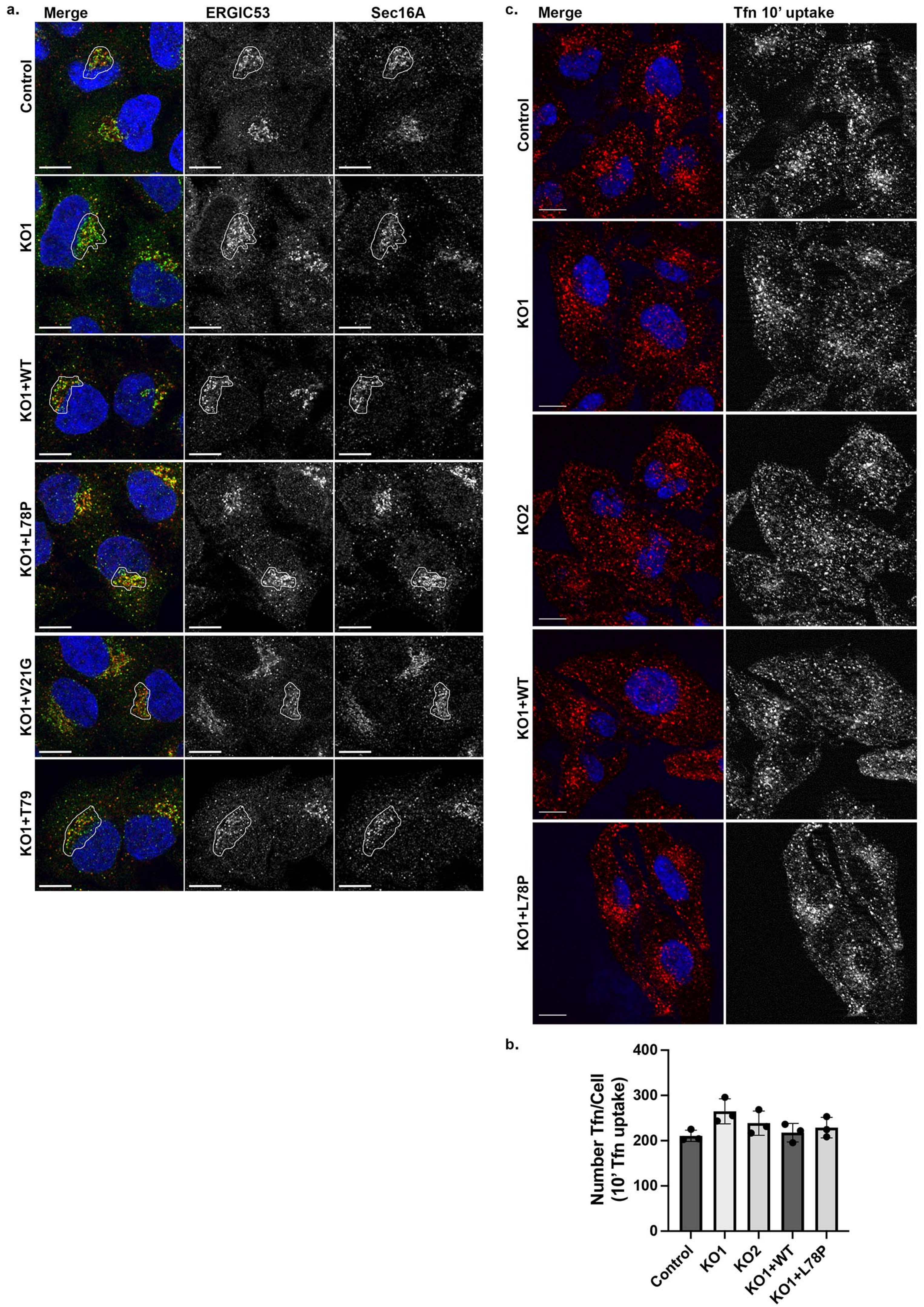

Suppl. Figure S6

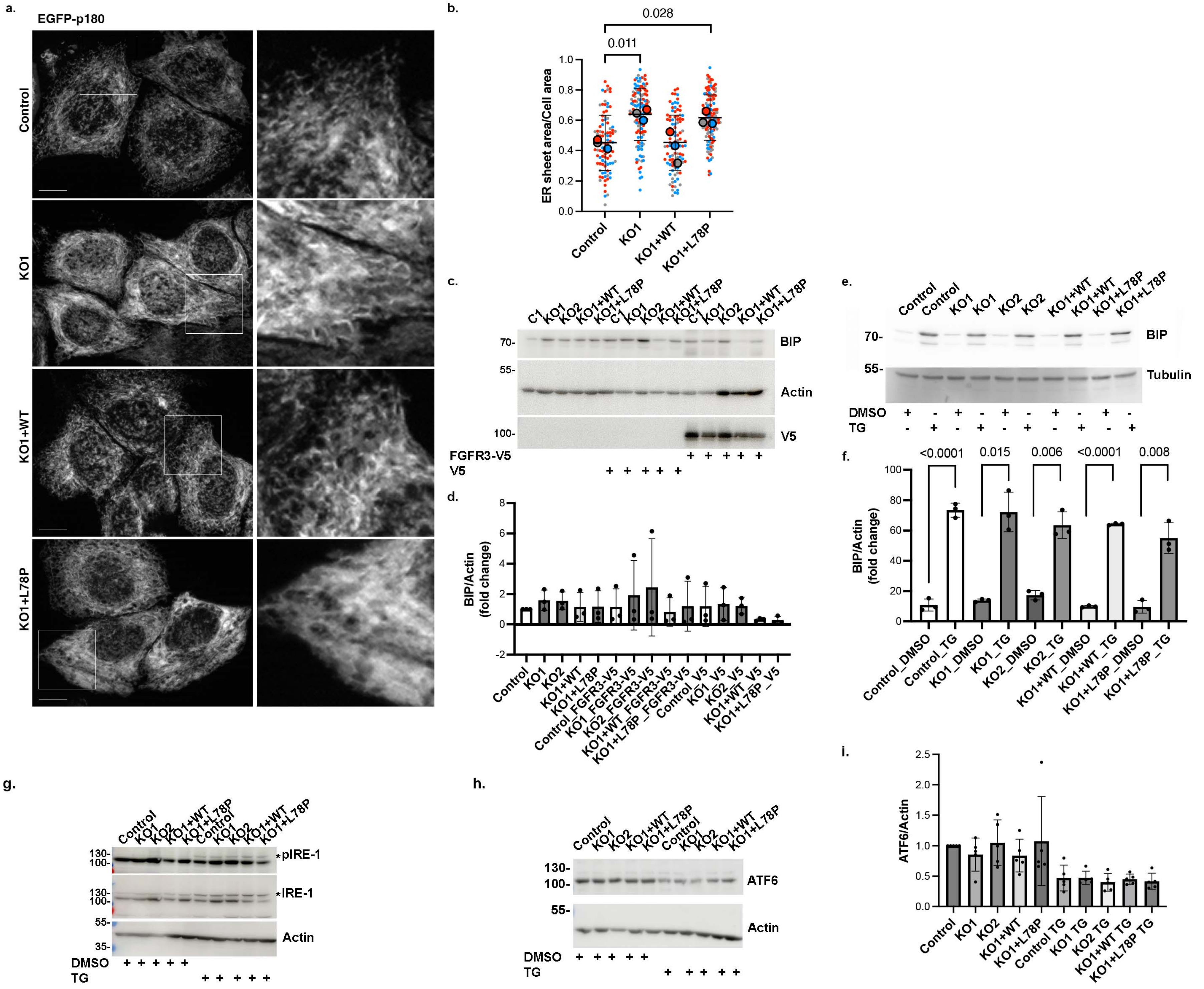

a.
EGFP-p180
Control
KO1
KO1+WT
KO1+L78P
b.
0.028
0.011
ER sheet area/Cell area
1.0
0.8
0.6
0.4
0.2
0.0
Control
KO1
KO1+WT
KO1+L78P
c.
C1 KO1 KO2 KO1+WT KO1+L78P C1 KO1 KO2 KO1+WT KO1+L78P C1 KO1 KO2 KO1+WT KO1+L78P
70-
BIP
55-
Actin
100-
V5
FGFR3-V5 + + + + +
V5 + + + + +
d.
BIP/Actin (fold change)
8
6
4
2
0
-2
Control
KO1
KO2
KO1+WT
KO1+L78P
Control_FGFR3-V5
KO1_FGFR3-V5
KO2_FGFR3-V5
KO1+WT_FGFR3-V5
KO1+L78P_FGFR3-V5
Control_V5
KO1_V5
KO2_V5
KO1+WT_V5
KO1+L78P_V5
e.
Control Control KO1 KO1 KO2 KO2 KO1+WT KO1+WT KO1+L78P KO1+L78P
70-
BIP
55-
Tubulin
DMSO + - + - + - + - + -
TG - + - + - + - + - +
f.
<0.0001
0.015
0.006
<0.0001
0.008
BIP/Actin (fold change)
100
80
60
40
20
0
Control_DMSO
Control_TG
KO1_DMSO
KO1_TG
KO2_DMSO
KO2_TG
KO1+WT_DMSO
KO1+WT_TG
KO1+L78P_DMSO
KO1+L78P_TG
g.
Control KO1 KO2 KO1+WT KO1+L78P Control KO1 KO2 KO1+WT KO1+L78P
130-
100-
*pIRE-1
130-
100-
*IRE-1
55-
35-
Actin
DMSO + + + + +
TG + + + + +
h.
Control KO1 KO2 KO1+WT KO1+L78P Control KO1 KO2 KO1+WT KO1+L78P
130-
100-
ATF6
55-
Actin
DMSO + + + + +
TG + + + + +
i.
ATF6/Actin
2.5
2.0
1.5
1.0
0.5
0.0
Control
KO1
KO2
KO1+WT
KO1+L78P
Control TG
KO1 TG
KO2 TG
KO1+WT TG
KO1+L78P TG

Suppl Fig. S7

a.

b.

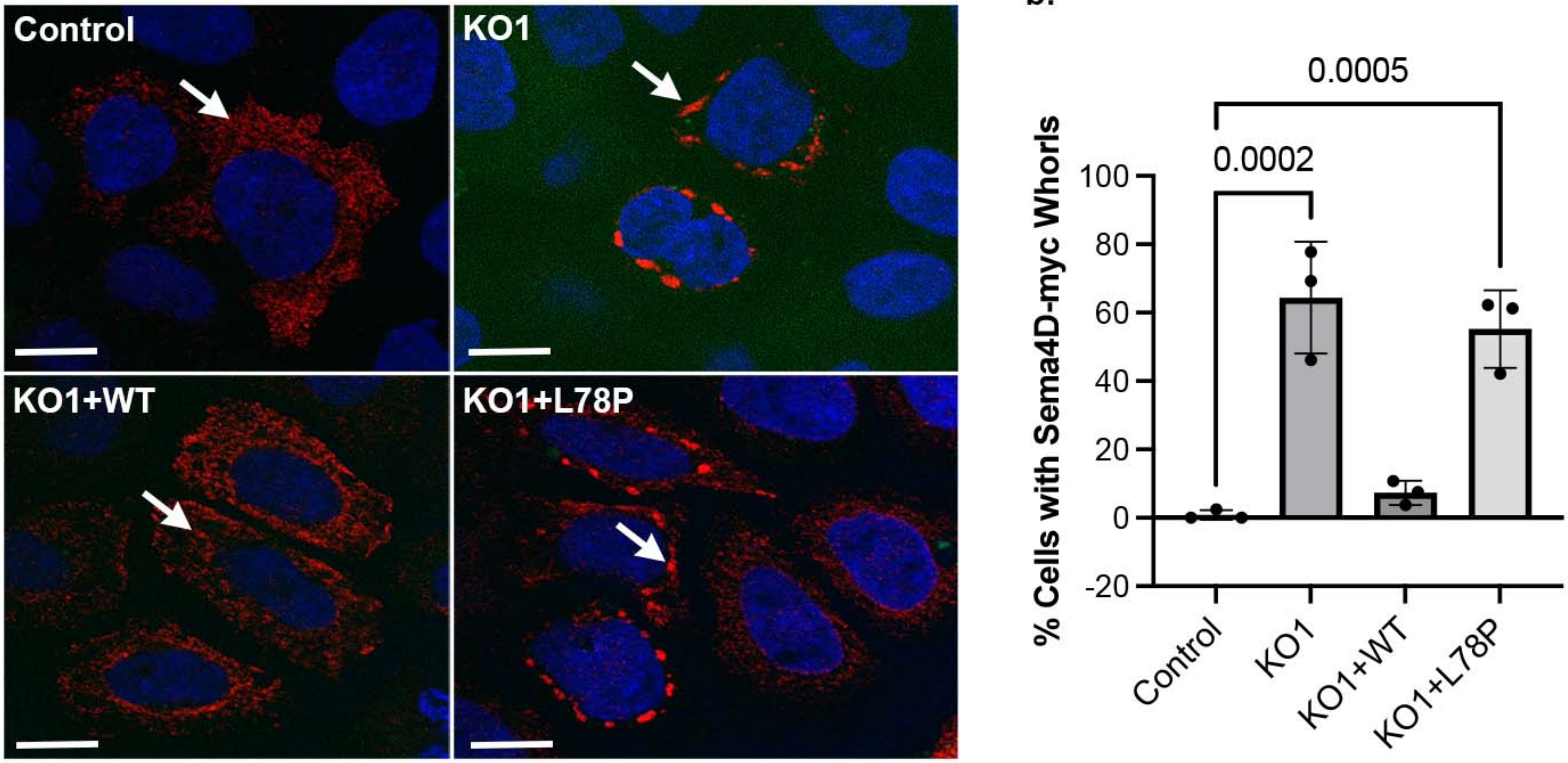

Suppl. Fig. S8

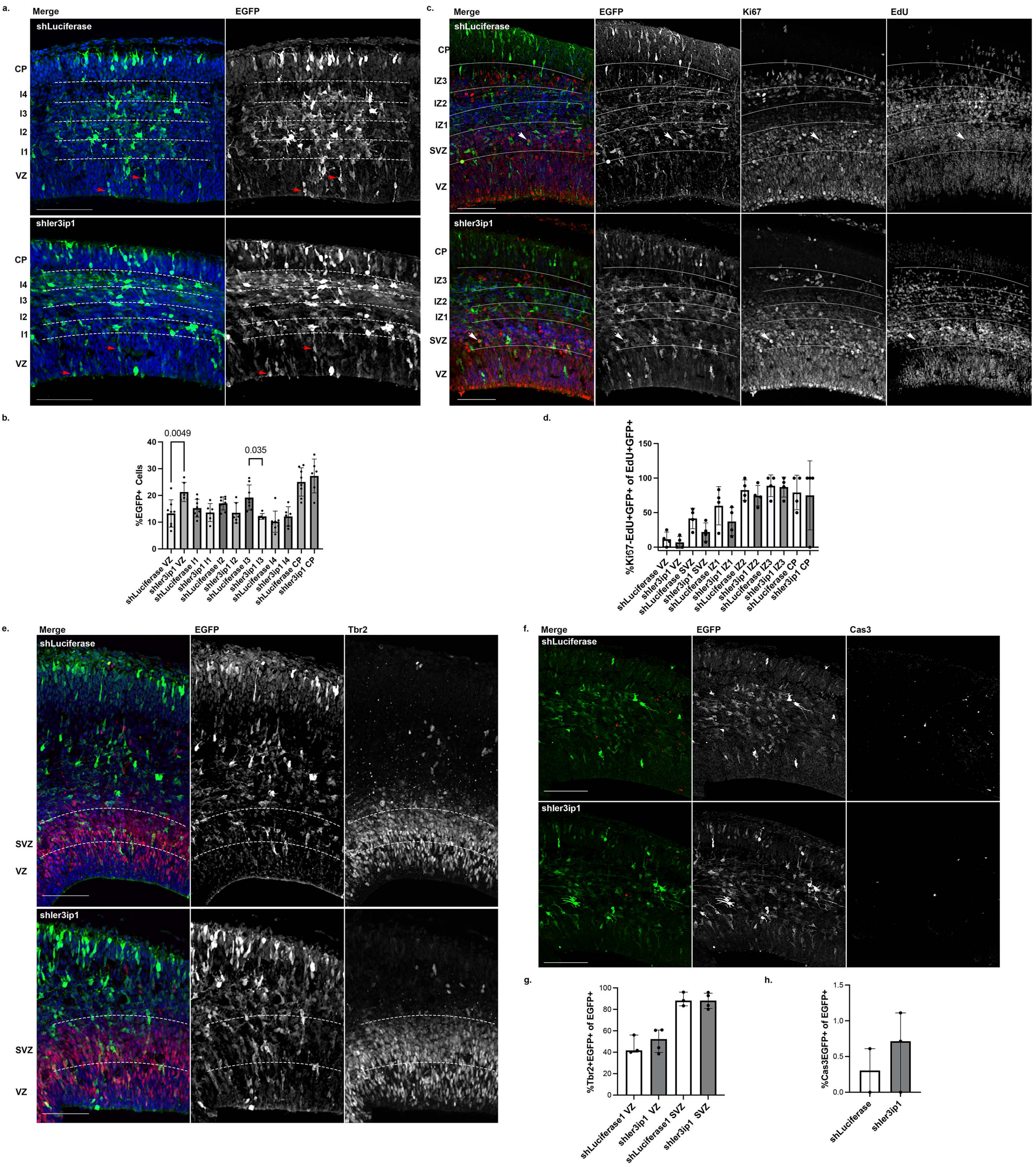
a.
Merge
EGFP
shLuciferase
shIer3ip1
CP
I4
I3
I2
I1
VZ
b.
0.0049
0.035
%EGFP+ Cells
shLuciferase VZ
shIer3ip1 VZ
shLuciferase I1
shIer3ip1 I1
shLuciferase I2
shIer3ip1 I2
shLuciferase I3
shIer3ip1 I3
shLuciferase I4
shIer3ip1 I4
shLuciferase CP
shIer3ip1 CP
c.
Merge
EGFP
Ki67
EdU
shLuciferase
shIer3ip1
CP
IZ3
IZ2
IZ1
SVZ
VZ
d.
%Ki67-EdU+GFP+ of EdU+GFP+
shLuciferase VZ
shIer3ip1 VZ
shLuciferase SVZ
shIer3ip1 SVZ
shLuciferase IZ1
shIer3ip1 IZ1
shLuciferase IZ2
shIer3ip1 IZ2
shLuciferase IZ3
shIer3ip1 IZ3
shLuciferase CP
shIer3ip1 CP
e.
Merge
EGFP
Tbr2
shLuciferase
shIer3ip1
SVZ
VZ
f.
Merge
EGFP
Cas3
shLuciferase
shIer3ip1
g.
%Tbr2+EGFP+ of EGFP+
shLuciferase1 VZ
shIer3ip1 VZ
shLuciferase1 SVZ
shIer3ip1 SVZ
h.
%Cas3EGFP+ of EGFP+
shLuciferase
shIer3ip1

Suppl. Fig. S9

Fig. 2f

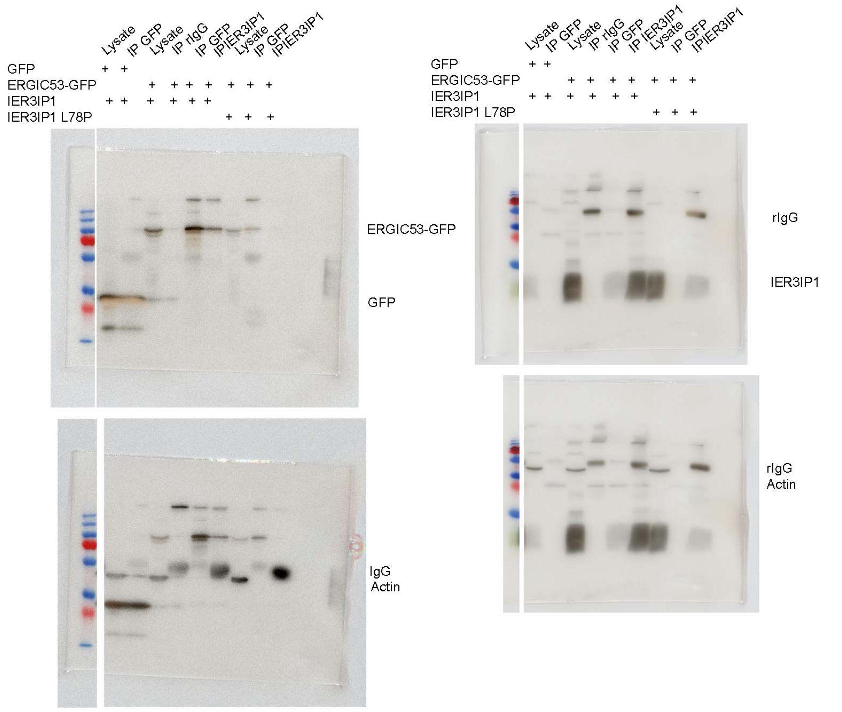

Fig. 3h

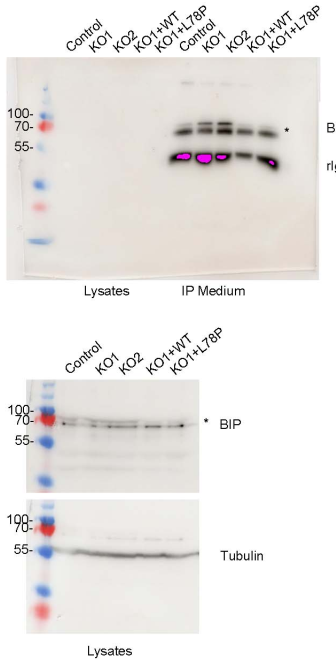

Fig. 3i

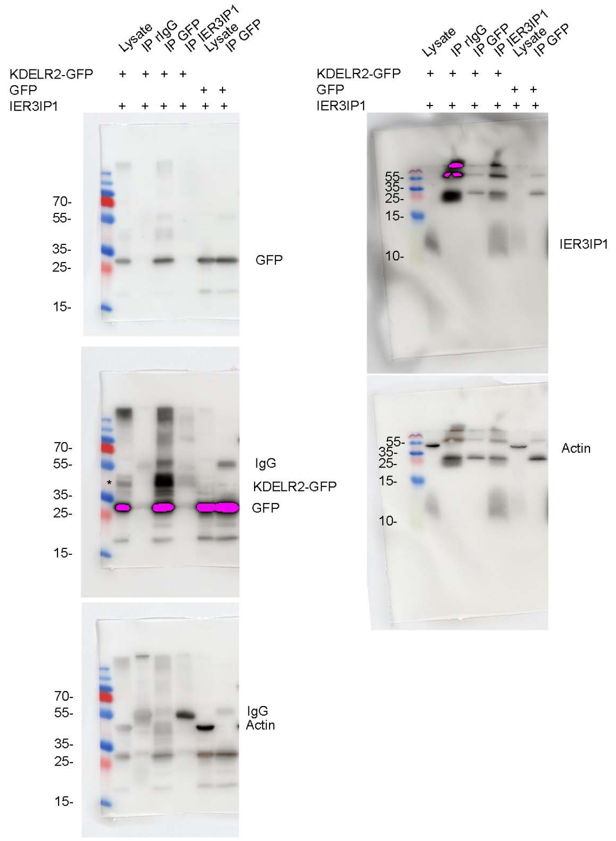

Fig. 5h, i

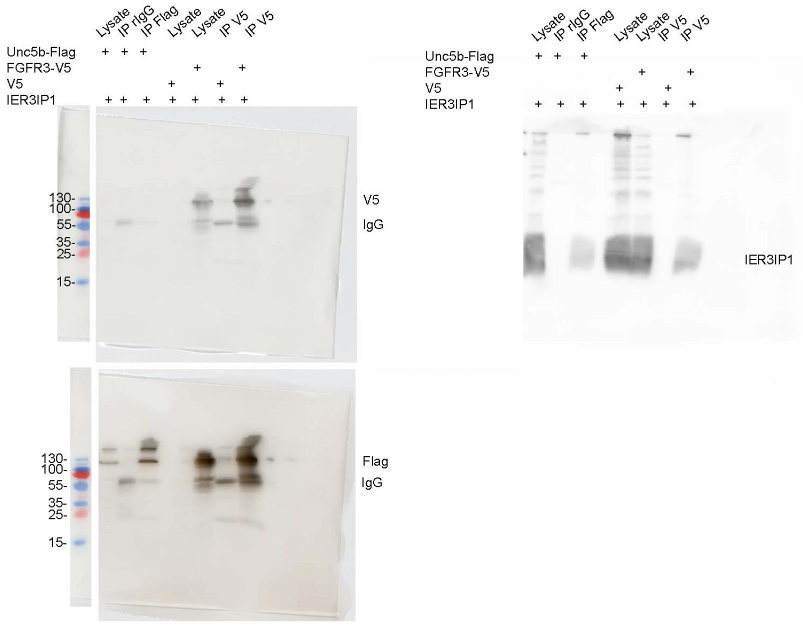

Supplement: Supplementary file 1 — Supplementary file1 (PDF 5504 KB) [file 18_2024_5386_MOESM1_ESM.pdf]
